# Synthesis and Biological Evaluation of Analogs of Didehydroepiandrosterone as Potential New Anticancer Agents

**DOI:** 10.3390/molecules25133052

**Published:** 2020-07-03

**Authors:** Eirik J. Solum, Sandra Liekens, Trond Vidar Hansen

**Affiliations:** 1 Faculty of Health Sciences, Nord University, 7801 Namsos, Norway; 2 Laboratory of Virology and Chemotherapy, Rega Institute for Medical Research, Department of Microbiology and Immunology, KU Leuven, Herestraat 49, Postbus 1043, B-3000 Leuven, Belgium; sandra.liekens@kuleuven.be; 3 School of Pharmacy, Department of Pharmaceutical Chemistry, University of Oslo, PO Box 1068 Blindern, N-0316 Oslo, Norway; t.v.hansen@farmasi.uio.no

**Keywords:** anti-cancer, anti-leukemia, CDK8 inhibition, steroids, cortistatin A

## Abstract

The synthesis, cytotoxicity and inhibition of CDK8 by thirteen analogs of cortistatin A are reported. These efforts revealed that the analogs with either a 6- or 7-isoquinoline or 5-indole side chain in the 17-position are the most promising anti-proliferative agents. These compounds showed potent cytotoxic effects in CEM, HeLa and HMEC-1 cells. All three compounds exhibited IC_50_ values < 10µM. The most interesting **10l** analog exhibited an IC_50_ value of 0.59 µM towards the human dermal microvascular endothelial cell line (HMEC-1), significantly lower than the reference standard 2-methoxyestradiol. At a concentration at 50 nM the most potent **10h** compound reduced the activity of CDK8 to 35%.

## 1. Introduction

**Figure 1:**
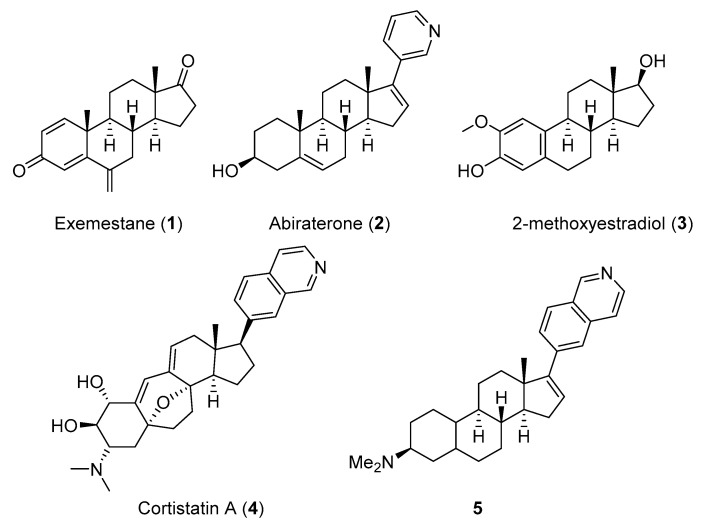
Chemical structures of biological active steroidal compounds.

Steroids are a class of biologically active molecules, important for an array of different physiological effects. Their affinities for various types of nuclear receptors, as well as their safe pharmacological profile have facilitated their widespread application in drug discovery and development [1]. Over recent years, an extensive focus on chemical modification of the steroidal structure has been seen. Such modifications of the steroidal nucleus have yielded several important anticancer molecules and lead compounds. Exemestane (**1**), [2] abiraterone (**2**) [3,4] and 2-methoxyestradiol (2-ME) (**3**) [5,6] are some of the successful examples that have emerged from steroidal pharmacophores, see Figure 1.

A class of compound, the cortistatins, exemplified by cortistatins A (**4**), see Figure 1, are examples of natural occurring steroid-like structures that have attracted great interest within anti-cancer research [7,8]. In 2006, cortistatin A (**4**) was the first compound of this group of steroidal-like alkaloids to be isolated from the marine sponge *Corticium simplex* [9]. The compound was later synthesized, and thoroughly evaluated for its biological effects [10,11,12,13]. Cortistatin A (**4**) exhibits potent anti-angiogenetic effects, proved by the inhibition of the proliferation of human umbilical vein endothelial cells (HUVECs) in the low nano-molar range [14]. Moreover, cortistatin A (**4**) is reported to have anti-leukemic activity in vitro and in vivo, and disproportionately induces upregulation of SE-associated genes in CA-sensitive AML cell lines [12]. In addition, several analogs of **5** with interesting anti-cancer effects have been prepared [6,7,8,15,16,17]. Structure-activity-relationship (SAR)-studies have revealed the presence of an isoquinoline group to be crucial for the activity and that the two hydroxyl-groups on the A ring are removable. Along these lines, one of the most interesting analogs reported so far is the amino steroid **5** reported by Corey and co-workers [7].

Herein we present our aim to investigate the biological effects of introducing a variety of nitrogen containing heterocycle systems, inspired by the above mentioned compounds, at the 17-position of didehydroepiandrosterone (DHEA) (**6**).

## 2. Results 

### 2.1. Chemistry

**Scheme 1:**
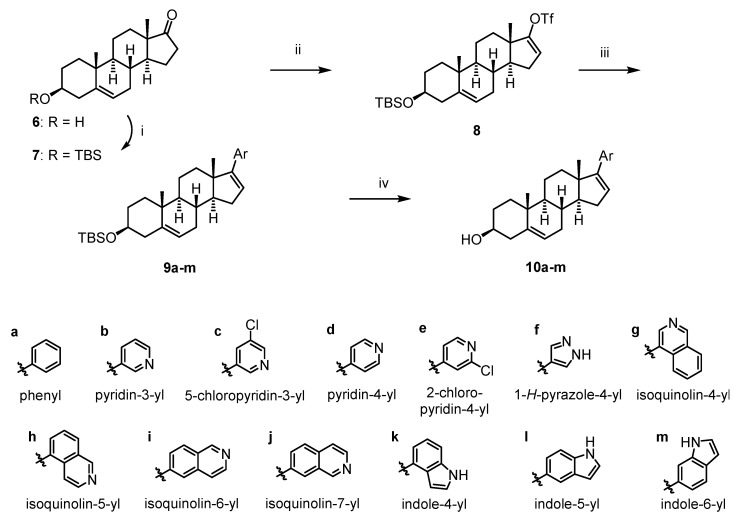
Synthesis of compounds **10a–10m**. Reagents and conditions: (i) TBSCl, imidazole, DMF rt., 5h, 96% (ii) KHMDS, PhN(SO_2_CF_3_)_2_, THF, −78 °C, 4 h, 89%; (iii) ArB(OH)_2_, Cs_2_CO_3_, Pd(Ph_3_P)_4_, THF:H_2_O (1:1) 60 °C, 5 h, 53–88%; (iv) TBAF, THF, rt., 41–89%.

The analogs **10a–10m** were prepared as depicted in Scheme 1. First, DHEA (**6**) was converted to the TBS-protected ketone **7**. Then the enol-triflate **8** was made from the ketone **7** in a reaction using *N*-phenyl-bis(trifluoromethanesulfonimide) in the presence of KHMDS at −78 °C. This sequence afforded compound **8** in 85% yield from dehydroepiandrosterone (**6**). Then compound **8** was employed in a Suzuki Miyaura reaction with different commercially available boronic acids. In the presence of Pd(PPh_3_)_4_ and Cs_2_CO_3_ in THF/H_2_O (1:1), the TBS protected compounds **9a–9m** were obtained (Scheme 1). Of note, the Suzuki Miyaura reaction with the chlorinated analogs (**9c** and **9e**) proved troublesome, due to the observation of polymerization of the chlorinated boronic acid. The polymeric material was hard to remove during work up, but by slowly adding a solution of the boronic acid in THF to the stirred reaction mixture, we managed to reduce the amount of polymeric material and isolate the desired compounds in decent yield (see Supporting Information). The desired analogs, **10a–10m**, were obtained after removal of the TBS-group under standard conditions using TBAF in THF.

### 2.2. Biological Evaluation

The prepared steroidal analogues **10a–10m** were evaluated, together with 2-ME (**3**), for their antiproliferative effects in two different cancer cell lines, human T-cell leukemia (CEM) and human cervix carcinoma (HeLa) as well as the human dermal microvascular endothelial cell-line HMEC-1. The data are expressed as IC_50_ (50% inhibitory concentration), which is defined as the compound concentration that reduces cell proliferation by 50%, and are shown in Table 1. The reference compound 2-ME (**3**) inhibited the growth of all cell lines tested in the low micromolar range (IC_50_ between 0.4 and 1.6 μM). Among the prepared analogues, the most potent compound proved to be the indole-5-yl analogue of DHEA (**10l**), with IC_50_ values of 1.5 ± 0.2 and 2.9 ± 1.0 μM towards the CEM and HeLa cell lines, respectively. Furthermore, the same compound showed potent inhibition of endothelial cell growth with an IC_50_ value of 0.59 ± 0.07 µM. The corresponding value of 2-ME (**3**) was 1.3 ± 0.5, which proved that compound **10l** was significantly more potent compared towards the HMEC-1 cell line. Unfortunately, among the other compounds the cytotoxic properties were either poor compared to the 2-ME (**3**) or not present at all. 

Inspired by the potent inhibitory activity of cortistatin A (**4**) towards CDK8, we decided to submit seven of the compounds to a CDK8 inhibition assay. The compounds were tested for their ability to inhibit probe binding to protein kinase CDK8 in vitro at 50 nM concentration. In this assay, the ability of a test compound to compete with an immobilized, active site directed ligand is quantitatively measured and reported as percent of DMSO control (POC), with lower numbers indicating higher binding affinity. The results are displayed in Table 1. Unfortunately, from the results obtained in this assay no correlation between the cytotoxicity of the compounds and their ability to inhibit CDK8 can be seen. The only compound with some probe-binding inhibition properties of CDK8 was compound **10h**. The compound has a isoquionline-5-yl side chain attached to the 5-membered D-ring of the steroid core structure. 

## 3. Materials and Methods

### 3.1. Chemistry

#### 3.1.1. General Methods

All reagents and solvents were used as purchased without further purification unless stated otherwise. Melting points are uncorrected. Analytical TLC was performed using silica gel 60 F254 aluminum plates (Merck). Flash column chromatography was performed on silica gel 60 (40–63 mm) produced by Merck. NMR spectra were recorded on a Bruker Avance DPX-300 MHz or DPX-400 MHz spectrometer for ^1^H-NMR, and 75 MHz or 101 MHz for ^13^C-NMR. Coupling constants (*J*) are reported in Hertz, and chemical shifts are reported in parts per million relative to CDCl_3_ (7.26 ppm for ^1^H and 77.0 ppm for ^13^C). Mass spectra were recorded at 70 eV with Fison’s VG Pro spectrometer. High-resolution mass spectra were performed with a VG Prospecmass spectrometer and with a Micromass Q-TOF-2™. Protocols for the preparation, physical and spectral data of the intermediates **7**, **8** and **9a–9m** are presented in the supplementary materials.

#### 3.1.2. A General Procedure for the Deprotection 

The TBS protected steroids **9a–9m** (0.15–0.2 mmol, 1 equiv.) were placed in a dry round-bottomed flask under an argon atmosphere, and dissolved in dry THF (3–4 mL). *Tert*-butylammoniumfluoride (1 M in THF, 1.1 equiv.) was added dropwise. The reaction mixture was stirred at room temperature (16–18 h). Upon completion, the reaction the mixture was poured into saturated aqueous NaHCO_3_ (10 mL), and extracted with ethyl acetate (4 × 5 mL). The combined organic extracts were dried (MgSO_4_) and the solvent evaporated *in vacuo*. The residues were purified by chromatography (silica gel, 20–50% ethyl acetate in hexane) to give the pure products. The reactions performed were followed by TLC, using cerium ammonium molybdate (CAM) stain to visualize the spots. 

*(8R,9S,10R,13S,14S)-10,13-Dimethyl-17-phenyl-2,3,4,7,8,9,10,11,12,13,14,15-dodecahydro-1H-cyclopenta[a]phenanthren-3-ol* (**10a**). Colorless solid (61 mg, 87%). Purified by column chromatography on silica gel using 20% ethyl acetate in heptane as eluent. R_f_ = 0.43 (20% ethyl acetate in heptane). ^1^H NMR (400 MHz, CDCl_3_) δ 7.37–7.32 (m, 2H), 7.29–7.15 (m, 3H), 6.00–5.68 (m, 1H), 5.50–5.22 (m, 1H), 3.64–3.36 (m, 1H), 2.37–2.14 (m, 3H), 2.12–1.95 (m, 3H), 1.88–1.38 (m, 9H), 1.12–0.99 (m, 8H), 0.89–0.82 (m, 1H). ^13^C NMR (101 MHz, CDCl_3_) δ 154.95, 141.27, 137.48, 128.22, 127.32, 126.83, 121.61, 71.90, 57.84, 50.61, 47.37, 42.49, 37.35, 36.87, 35.57, 31.81, 31.76, 31.73, 30.66, 21.10, 19.49, 16.78. HRMS (EI): Exact mass calculated for C_25_H_32_NO [M + H]^+^: 348.2453, found 348.2449. 

*(3S,8R,9S,10R,13S,14S)-10,13-Dimethyl-17-(pyridin-3-yl)-2,3,4,7,8,9,10,11,12,13,14,15-dodecahydro-1H-cyclopenta[a]phenanthren-3-ol* (**10b**). Colourless solid (62 mg, 89%). Purified by column chromatography on silica gel using 50% ethyl acetate in heptane as eluent. R_f_ = 0.21 (50% ethyl acetate in heptane). ^1^H NMR (400 MHz, CDCl_3_) δ 8.61 (d, J = 2.5 Hz, 1H), 8.45 (dd, J = 4.8, 1.7 Hz, 1H), 7.64 (dt, J = 7.9, 1.9 Hz, 1H), 7.25–7.17 (m, 1H), 5.99 (dd, J = 3.3, 1.8 Hz, 1H), 5.52–5.27 (m, 1H), 3.60–3.48 (m, 1H), 2.38–2.20 (m, 3H), 2.15–2.00 (m, 3H), 1.90–1.41 (m, 9H), 1.15–1.01 (m, 8H). ^13^C NMR (101 MHz, CDCl_3_) δ 151.81, 147.95, 147.85, 141.34, 133.90, 133.17, 129.42, 123.18, 121.42, 71.79, 57.71, 50.53, 47.50, 42.47, 37.34, 36.85, 35.42, 31.96, 31.79, 31.67, 30.61, 21.03, 19.48, 16.72. HRMS (EI): Exact mass calculated for C_24_H_31_NO [M + H]^+^: 349.2406, found 349.2411.

*(3S,8R,9S,10R,13S,14S)-17-(5-Chloropyridin-3-yl)-10,13-dimethyl-2,3,4,7,8,9,10,11,12,13,14,15-dodecahydro-1H-cyclopenta[a]phenanthren-3-ol* (**10c**). Colorless solid (56 mg, 73%). Purified by column chromatography on silica gel using 50% ethyl acetate in heptane as eluent. R_f_ = 0.48 (50% ethyl acetate in heptane). ^1^H NMR (400 MHz, CDCl_3_) δ 8.49 (d, J = 1.9 Hz, 1H), 8.42 (d, J = 2.3 Hz, 1H), 7.64 (t, J = 2.1 Hz, 1H), 6.05 (dd, J = 3.3, 1.8 Hz, 1H), 5.39 (d, J = 5.2 Hz, 1H), 3.76–3.45 (m, 1H), 2.38–2.21 (m, 3H), 2.13–1.99 (m, 3H), 1.89–1.81 (m, 2H), 1.81–1.42 (m, 8H), 1.15–1.08 (m, 1H), 1.07 (s, 3H), 1.04 (s, 3H). ^13^C NMR (101 MHz, CDCl_3_) δ 150.39, 146.43, 145.50, 141.15, 134.21, 133.40, 131.63, 130.95, 121.25, 71.67, 57.53, 50.30, 47.39, 42.28, 37.16, 36.69, 35.17, 31.88, 31.62, 31.47, 30.41, 20.84, 19.34, 16.60. HRMS (EI): Exact mass calculated for C_24_H_30_ClNO [M + H]^+^: 383.2016, found 383.2014.

*(3S,8R,9S,10R,13S,14S)-10,13-dimethyl-17-(pyridin-4-yl)-2,3,4,7,8,9,10,11,12,13,14,15-dodecahydro-1H-cyclopenta[a]phenanthren-3-ol* (**10d**). Colorless solid (62 mg, 88%). Purified by column chromatography on silica gel using 50% ethyl acetate in heptane as eluent. R_f_ = 0.17 (50% ethyl acetate in heptane).^1^H NMR (400 MHz, CDCl_3_) δ 8.50 (d, J = 5.0 Hz, 2H), 7.26 (dd, J = 4.6, 1.6 Hz, 2H), 6.18 (dd, J = 3.3, 1.8 Hz, 1H), 5.42–5.36 (m, 1H), 3.61–3.47 (m, 1H), 2.38–2.20 (m, 3H), 2.17–2.00 (m, 3H), 1.90–1.40 (m, 9H), 1.17–1.00 (m, 8H). ^13^C NMR (101 MHz, CDCl_3_) δ 152.61, 149.57, 144.94, 141.34, 131.83, 121.39, 121.36, 71.82, 57.71, 50.50, 47.31, 42.46, 37.33, 36.85, 35.31, 32.00, 31.79, 31.64, 30.54, 21.01, 19.49, 16.77. HRMS (EI): Exact mass calculated for C_24_H_31_NO [M + H]^+^: 349.2406, found 349.2405.

*(3S,8R,9S,10R,13S,14S)-17-(2-Chloropyridin-4-yl)-10,13-dimethyl-2,3,4,7,8,9,10,11,12,13,14,15-dodecahydro-1H-cyclopenta[a]phenanthren-3-ol* (**10e**). Colorless solid (50 mg, 65%). Purified by column chromatography on silica gel using 50% ethyl acetate in heptane as eluent. R_f_ = 0.58 (50% ethyl acetate in heptane). ^1^H NMR (400 MHz, CDCl_3_) δ 8.27 (d, J = 5.2 Hz, 1H), 7.29 (d, J = 1.4 Hz, 1H), 7.18 (dd, J = 5.2, 1.5 Hz, 1H), 6.22 (dd, J = 3.3, 1.9 Hz, 1H), 5.39 (dt, J = 5.2, 2.0 Hz, 1H), 3.54 (tt, J = 11.3, 4.6 Hz, 1H), 2.39–2.20 (m, 3H), 2.14–2.00 (m, 3H), 1.90–1.81 (m, 2H), 1.80–1.39 (m, 8H), 1.17–1.01 (m, 7H). ^13^C NMR (101 MHz, CDCl_3_) δ 151.65, 151.47, 149.31, 147.79, 141.16, 133.17, 121.33, 121.20, 119.92, 71.66, 57.51, 50.27, 47.20, 42.27, 37.15, 36.68, 35.04, 31.91, 31.61, 31.44, 30.34, 20.81, 19.33, 16.62. HRMS (EI): Exact mass calculated for C_24_H_30_ClNO [M + H]^+^: 383.2016, found 383.2012.

*(3S,8R,9S,10R,13S,14S)-10,13-Dimethyl-17-(1H-pyrazol-4-yl)-2,3,4,7,8,9,10,11,12,13,14,15-dodecahydro-1H-cyclopenta[a]phenanthren-3-ol* (**10f**). Colorless solid (28 mg, 41%). Purified by column chromatography on silica gel using 50% ethyl acetate in heptane as eluent. R_f_ = 0.16 (50% ethyl acetate in heptane). ^1^H NMR (400 MHz, DMSO) δ 12.69 (s, 1H), 7.66 (s, 2H), 5.76 (dd, J = 3.1, 1.7 Hz, 1H), 5.50–5.10 (m, 1H), 4.59 (d, J = 4.5 Hz, 1H), 3.28–3.14 (m, 1H), 2.23–2.05 (m, 4H), 2.05–1.88 (m, 2H), 1.83–1.29 (m, 9H), 1.06–0.94 (m, 5H), 0.90 (s, 3H). ^13^C NMR (101 MHz, DMSO) δ 145.95, 141.57, 121.66, 120.26, 116.06, 69.98, 56.61, 50.08, 46.33, 42.25, 40.15, 36.83, 36.27, 34.72, 31.41, 31.03, 30.90, 29.96, 20.53, 19.06, 15.92. HRMS (EI): Exact mass calculated for C_22_H_30_N_2_O [M + H]^+^: 338.2358, found 338.2367.

*(3S,8R,9S,10R,13S,14S)-17-(Isoquinolin-4-yl)-10,13-dimethyl-2,3,4,7,8,9,10,11,12,13,14,15-dodecahydro-1H-cyclopenta[a]phenanthren-3-ol* (**10g**). Colorless solid (64 mg, 80%). Purified by column chromatography on silica gel using 50% ethyl acetate in heptane as eluent. R_f_ = 0.27 (50% ethyl acetate in heptane). ^1^H NMR (400 MHz, CDCl_3_) δ 9.16 (d, J = 0.9 Hz, 1H), 8.33 (s, 1H), 8.03 (dd, J = 8.5, 1.1 Hz, 1H), 7.97 (dt, J = 8.0, 1.1 Hz, 1H), 7.73–7.63 (m, 1H), 7.65–7.55 (m, 1H), 5.87 (dd, J = 3.1, 1.6 Hz, 1H), 5.50–5.34 (m, 1H), 3.68–3.42 (m, 1H), 2.48–2.37 (m, 1H), 2.40–2.18 (m, 3H), 2.19–2.09 (m, 1H), 1.90–1.71 (m, 5H), 1.63–1.43 (m, 5H), 1.17–1.03 (m, 5H), 1.01 (s, 3H). ^13^C NMR (101 MHz, CDCl_3_) δ 151.16, 149.65, 141.48, 141.38, 135.81, 132.05, 130.31, 129.54, 128.58, 127.87, 127.18, 125.69, 121.49, 71.87, 57.65, 50.78, 49.75, 42.48, 37.37, 36.94, 35.24, 32.62, 31.86, 31.81, 31.07, 21.01, 19.50, 16.42. HRMS (EI): Exact mass calculated for C_28_H_33_NO [M + H]^+^: 399.2562, found 399.2561.

*(3S,8R,9S,10R,13S,14S)-17-(Isoquinolin-5-yl)-10,13-dimethyl-2,3,4,7,8,9,10,11,12,13,14,15-dodecahydro-1H-cyclopenta[a]phenanthren-3-ol* (**10h**). Colorless solid (62 mg, 77%). Purified by column chromatography on silica gel using 50% ethyl acetate in heptane as eluent. R_f_ = 0.29 (50% ethyl acetate in heptane). ^1^H NMR (400 MHz, CDCl_3_) δ 9.21 (s, 1H), 8.47 (d, J = 5.9 Hz, 1H), 7.90–7.81 (m, 2H), 7.59–7.53 (m, 1H), 7.48 (dd, J = 7.2, 1.3 Hz, 1H), 5.79 (dd, J = 3.1, 1.6 Hz, 1H), 5.55–5.24 (m, 1H), 3.77–3.36 (m, 1H), 2.45–2.07 (m, 5H), 1.89–1.68 (m, 5H), 1.62–1.43 (m, 5H), 1.16–1.03 (m, 5H), 0.99 (s, 3H). ^13^C NMR (101 MHz, CDCl_3_) δ 152.66, 151.24, 142.88, 141.39, 135.54, 135.15, 130.95, 129.61, 129.11, 126.63, 126.44, 121.40, 119.35, 71.72, 57.64, 50.74, 49.62, 42.46, 37.34, 36.90, 35.24, 32.47, 31.82, 31.76, 30.99, 20.96, 19.47, 16.46. HRMS (EI): Exact mass calculated for C_28_H_33_NO [M + H]^+^: 399.2562, found 399.2561.

*(3S,8R,9S,10R,13S,14S)-17-(Isoquinolin-6-yl)-10,13-dimethyl-2,3,4,7,8,9,10,11,12,13,14,15-dodecahydro-1H-cyclopenta[a]phenanthren-3-ol* (**10i**). Colorless solid (52 mg, 87%). Purified by column chromatography on silica gel using 50% ethyl acetate in heptane as eluent. R_f_ = 0.24 (50% ethyl acetate in heptane). ^1^H NMR (400 MHz, CDCl_3_) δ 9.19 (s, 1H), 8.49 (d, J = 5.8 Hz, 1H), 7.88 (dd, J = 8.4, 0.9 Hz, 1H), 7.80–7.74 (m, 1H), 7.66 (dd, J = 8.6, 1.6 Hz, 1H), 7.64–7.57 (m, 1H), 6.16 (dd, J = 3.3, 1.8 Hz, 1H), 5.44–5.37 (m, 1H), 3.62–3.48 (m, 1H), 2.38–2.24 (m, 3H), 2.21 (dt, J = 12.2, 3.6 Hz, 1H), 2.17–2.04 (m, 2H), 1.92–1.45 (m, 9H), 1.15 (s, 5H), 1.09 (s, 3H). ^13^C NMR (101 MHz, CDCl_3_) δ 154.26, 152.07, 143.22, 141.32, 139.49, 136.13, 130.53, 127.72, 127.42, 127.30, 123.05, 121.50, 120.77, 71.86, 57.88, 50.55, 47.61, 42.48, 37.35, 36.87, 35.62, 32.05, 31.81, 31.70, 30.65, 21.12, 19.51, 16.89. HRMS (EI): Exact mass calculated for C_28_H_33_NO [M + H]^+^: 399.2562, found 399.2563.

*(3S,8R,9S,10R,13S,14S)-17-(Isoquinolin-7-yl)-10,13-dimethyl-2,3,4,7,8,9,10,11,12,13,14,15-dodecahydro-1H-cyclopenta[a]phenanthren-3-ol* (**10j**). Colorless solid (50 mg, 83%). Purified by column chromatography on silica gel using 50% ethyl acetate in heptane as eluent. R_f_ = 0.26 (50% ethyl acetate in heptane). ^1^H NMR (400 MHz, DMSO) δ 9.32 (s, 1H), 8.44 (d, J = 5.6 Hz, 1H), 8.08 (d, J = 1.8 Hz, 1H), 7.93–7.82 (m, 2H), 7.81–7.72 (m, 1H), 6.33–6.15 (m, 1H), 5.41–5.21 (m, 1H), 4.60 (d, J = 4.5 Hz, 1H), 3.29–3.22 (m, 1H), 2.36–1.99 (m, 6H), 1.85–1.50 (m, 7H), 1.49–1.32 (m, 2H), 1.14 (s, 3H), 1.06–0.95 (m, 5H). ^13^C NMR (101 MHz, DMSO) δ 153.18, 152.54, 142.61, 141.61, 135.26, 134.05, 129.80, 129.13, 128.36, 126.36, 123.32, 120.23, 119.90, 69.98, 57.21, 49.84, 46.67, 42.24, 36.83, 36.26, 34.66, 31.40, 31.27, 30.96, 29.97, 20.52, 19.06, 16.27. HRMS (EI): Exact mass calculated for C_28_H_33_NO [M + H]^+^: 399.2562, found 399.2561.

*(3S,8R,9S,10R,13S,14S)-17-(1H-indol-4-yl)-10,13-Dimethyl-2,3,4,7,8,9,10,11,12,13,14,15-dodecahydro-1H-cyclopenta[a]phenanthren-3-ol* (**10k**). Colorless solid (60 mg, 78%). Purified by column chromatography on silica gel using 50% ethyl acetate in heptane as eluent. R_f_ = 0.21 (50% ethyl acetate in heptane). ^1^H NMR (400 MHz, DMSO) δ 11.05 (s, 1H), 7.32–7.21 (m, 2H), 7.02 (t, J = 7.7 Hz, 1H), 6.90 (dd, J = 7.4, 1.0 Hz, 1H), 6.49–6.43 (m, 1H), 5.96 (dd, J = 3.1, 1.7 Hz, 1H), 5.33 (d, J = 5.1 Hz, 1H), 4.59 (d, J = 4.5 Hz, 1H), 3.30–3.22 (m, 1H), 2.34–2.26 (m, 1H), 2.23–2.00 (m, 4H), 1.95–1.88 (m, 1H), 1.80–1.29 (m, 9H), 1.02 (d, J = 9.7 Hz, 8H). ^13^C NMR (101 MHz, DMSO) δ 153.28, 141.59, 136.06, 128.78, 127.49, 126.77, 124.76, 120.40, 120.30, 116.87, 110.07, 101.27, 69.98, 57.00, 50.07, 47.71, 42.26, 36.86, 36.30, 35.08, 31.54, 31.42, 31.10, 30.18, 20.51, 19.09, 16.69. HRMS (EI): Exact mass calculated for C_27_H_33_NO [M + H]^+^: 387.2562, found 387.2562.

*(3S,8R,9S,10R,13S,14S)-17-(1H-Indol-5-yl)-10,13-dimethyl-2,3,4,7,8,9,10,11,12,13,14,15-dodecahydro-1H-cyclopenta[a]phenanthren-3-ol* (**10l**). Colorless solid (56 mg, 72%). Purified by column chromatography on silica gel using 50% ethyl acetate in heptane as eluent. R_f_ = 0.18 (50% ethyl acetate in heptane). ^1^H NMR (400 MHz, DMSO) δ 11.00 (s, 1H), 7.52 (d, J = 1.9 Hz, 1H), 7.37–7.25 (m, 2H), 7.13 (dd, J = 8.4, 1.7 Hz, 1H), 6.45–6.34 (m, 1H), 5.81 (dd, J = 3.0, 1.6 Hz, 1H), 5.32 (d, J = 4.8 Hz, 1H), 4.65–4.53 (m, 1H), 3.29–3.22 (m, 1H), 2.23–2.10 (m, 4H), 2.06–1.94 (m, 2H), 1.82–1.30 (m, 9H), 1.08–0.95 (m, 8H). ^13^C NMR (101 MHz, DMSO) δ 155.31, 141.58, 134.96, 127.51, 125.38, 123.98, 120.37, 120.27, 117.46, 111.05, 101.28, 69.98, 57.30, 49.95, 46.64, 42.25, 36.84, 36.26, 35.17, 31.40, 31.03, 31.00, 30.07, 20.53, 19.06, 16.50. HRMS (EI): Exact mass calculated for C_27_H_33_NO [M + H]^+^: 387.2562, found 387.2563.

*(3S,8R,9S,10R,13S,14S)-17-(1H-Indol-6-yl)-10,13-dimethyl-2,3,4,7,8,9,10,11,12,13,14,15-dodecahydro-1H-cyclopenta[a]phenanthren-3-ol* (**10m**). Colorless solid (53 mg, 69%). Purified by column chromatography on silica gel using 50% ethyl acetate in heptane as eluent. R_f_ = 0.25 (50% ethyl acetate in heptane). ^1^H NMR (400 MHz, DMSO) δ 10.91 (s, 1H), 7.41 (d, J = 8.3 Hz, 1H), 7.35 (s, 1H), 7.26 (t, J = 2.7 Hz, 1H), 7.02 (dd, J = 8.3, 1.5 Hz, 1H), 6.45–6.17 (m, 1H), 5.92–5.73 (m, 1H), 5.42–5.12 (m, 1H), 4.54 (s, 1H), 3.26–3.18 (m, 1H), 2.21–2.06 (m, 4H), 2.03–1.93 (m, 2H), 1.79–1.26 (m, 9H), 1.06–0.91 (m, 8H). ^13^C NMR (101 MHz, DMSO) δ 155.05, 141.59, 135.93, 129.50, 126.49, 125.45, 124.72, 120.27, 119.59, 118.25, 108.80, 100.85, 69.98, 57.27, 49.93, 46.67, 42.25, 36.84, 36.27, 35.21, 31.41, 31.03, 30.06, 20.53, 19.07, 16.54. HRMS (EI): Exact mass calculated for C_27_H_33_NO [M + H]^+^: 387.2562, found 387.2563.

### 3.2. Biological Evaluation

#### 3.2.1. Cell Studies

Human cervical carcinoma (HeLa) cells were seeded in 96-well plates at 15,000 cells/well in the presence of 5-fold dilutions of the compounds. After 3 days of incubation, the cells were trypsinized and counted by means of a Coulter counter (Analis, Leuven, Belgium). Human dermal microvascular endothelial (HMEC-1) cells were seeded on gelatin- coated 48-well plates at 20,000 cells/well. After overnight incubation, 5-fold dilutions of the compounds were added. Three days later, the cells were trypsinized and counted. Human T-cell leukemia (CEM) cells were seeded in 96-well plates at 60,000 cells/well in the presence of the compounds, allowed to proliferate for 4 days and then counted. The 50% inhibitory concentration (IC_50_) was defined as the compound concentration required to reduce cell proliferation by 50% [18].

#### 3.2.2. Protein Kinase Assay

For the CDK8 profiling we used a kinase selectivity and profiling assay (DiscoveRx) [19]. The assay uses kinase-tagged T7 phage strains which were grown in parallel in 24-well blocks in an *Escherichia coli* host derived from the BL21 strain. *E. coli* were grown to log-phase and infected with T7 phage from a frozen stock (multiplicity of infection = 0.4) and incubated with shaking at 32 °C until lysis (90–150 min). The lysates were centrifuged (6000× *g*) and filtered (0.2 μm) to remove cell debris. Streptavidin-coated magnetic beads were treated with biotinylated small molecule ligands for 30 min at room temperature to generate affinity resins for kinase assays. The liganded beads were blocked with excess biotin and washed with blocking buffer (SeaBlock (Pierce), 1% BSA, 0.05% Tween 20, 1 mM DTT) to remove unbound ligand and to reduce non-specific phage binding. Binding reactions were assembled by combining kinases, liganded affinity beads, and test compounds in 1× binding buffer (20% SeaBlock, 0.17× PBS, 0.05% Tween 20, 6 mM DTT). Test compounds were prepared as 40× stocks in 100% DMSO and directly diluted into the assay. All reactions were performed in polypropylene 384-well plates in a final volume of 20 µL. The assay plates were incubated at room temperature with shaking for 1 h and the affinity beads were washed with wash buffer (1× PBS, 0.05% Tween 20). The beads were then re-suspended in elution buffer (1× PBS, 0.05% Tween 20, 0.5 μM non-biotinylated affinity ligand) and incubated at room temperature with shaking for 30 min. The kinase concentration in the eluates was measured by qPCR. The test compounds were screened at 50 nM, and results for primary screen binding interactions are reported as POC (percent of control). The negative control consists of adding an equal DMSO volume without a test compound and the positive control consist of a control compound. From this POC is calculated: ((test compound signal–positive control signal)/(negative control signal–positive control signal)) × 100%. Negative control = DMSO (100% Ctrl); Positive control = control compound (0% Ctrl), where lower numbers indicate stronger hits in the matrix.

## 4. Conclusions

Structure-activity relationship (SAR) studies have revealed that steroidal analogs of cortistatin A to act as novel leads for further anti-cancer drug development mediated by inhibition of CDK8 [7,8,20]. In total, 13 new analogs of cortistatin A **4** have been prepared using the Suzuki–Miyaura reaction. All analogs were evaluated for their cytotoxic effects. Additionally, some of the analogs were evaluated for their ability to inhibit CDK8 at 50 nM concentration. The most cytotoxic compounds proved to be compounds **10i**, **j** and **l**, with either a 6- or 7-isoquinoline attached in the 17 position of the steroidal nucleus This is in accordance with previous literature based on 3-amminosteroids [7,17]. However, the compounds proved less potent than the included standard 2-ME **2**. One exception was compound **10l**, which exhibited an IC_50_-value of 0.59 µM towards the HMEC-1 cell line. The compound has a 5-indol ring attached to the 17-position of the steroidal core. No correlation between the ability to inhibit CDK8 and the cytotoxicity was observed, as the most prominent CDK8 inhibitor proved to be compound **10h**, with a 5-isoquinoline sidechain attached. Hence, the mechanism behind the toxicity of these compounds cannot be related to inhibition of these enzymes.

## Figures and Tables

**Table 1:** Biological evaluation of compounds **10a–10m**.

Compound	CEM Cell Assay IC_50_ (µM) ^a^	HeLa Cell Assay IC_50_ (µM) ^a^	HMEC-1 Cell Assay IC_50_ (µM) ^a^	CDK8 Per Cent of Control (POC)
**10a**	>100	>100	>100	n.d. ^b^
**10b**	16 ± 3	90 ± 14	17 ± 4	n.d.
**10c**	18 ± 4	97 ± 4	60 ± 4	95%
**10d**	>100	2.1 ± 1.7	88 ± 8	n.d.
**10e**	10 ± 1	18 ± 11	8.2 ± 1.9	100%
**10f**	24 ± 16	7.0 ± 2.5	11 ± 1	n.d.
**10g**	14 ± 1	24 ± 6	15 ± 0	n.d.
**10h**	16 ± 2	24 ± 8	13 ± 0	32%
**10i**	5.4 ± 0.6	9.2 ± 0.9	2.6 ± 0.4	100%
**10j**	5.8 ± 3.1	8.7 ± 0.0	5.4 ± 3.6	100%
**10k**	26 ± 17	91 ± 12	8.4 ± 4.3	100%
**10l**	1.5 ± 0.2	2.9 ± 1.0	0.59 ± 0.07	100%
**10m**	50 ± 21	44 ± 2	14 ± 2	n.d.
**2-ME (3)**	1.6 ± 0.9	0.41 ± 0.06	1.3 ± 0.5	n.d.

^a^ Results of three experiments performed as triplicates, ^b^ n.d = not determined.

## Supplementary Materials

The following are available online, Protocols for the preparation, physical and spectral data of the intermediates **7**, **8** and **9a–9m**.
