# Supplementary material for: Synthesis and Biological Evaluation of Analogs of Didehydroepiandrosterone as Potential New Anticancer Agents
Source: Molecules. 2020 Jul 3;25(13):3052. doi: 10.3390/molecules25133052 (PMC7412091; doi:10.3390/molecules25133052)
Supplement: Supplementary file 1 [file molecules-25-03052-s001.pdf]

3-tert-butyldimethylsiloxy-dehydroepiandrosterone

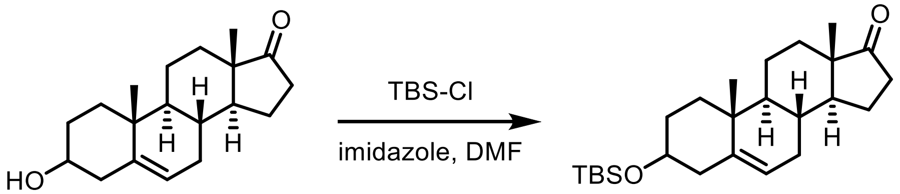

Dehydroepiandrosterone (2.88 g, 10.0 mmol) was placed in a 250 mL round bottomed flask and dissolved in dry DMF (50 mL). Imidazole (0.817 g, 12.0 mmol) and TBSCl (1.583 g, 10.50 mmol) were added. The reaction mixture was stirred at room temperature under argon atmosphere for 5 h. Saturated aqeous NaCl (30 mL) was added, and the mixture was extracted with ethyl acetate (4 x 20 mL). The combined organic extract was washed with brine (20 mL), aqueous AcOH (10%, 20 mL) and aqueous $NaHCO_3$ (20 mL) before it was dried ($MgSO_4$) and evaporated *in vacuo*. The product was purified by flash chromatography (silica gel, 30% ethyl acetate in hexane) to give the pure product as white solid in 96% yield 3.86 g. ^{1}H NMR (400 MHz, $CDCl_3$) δ 5.48 – 5.24 (m, 1H), 3.67 – 3.33 (m, 1H), 2.56 – 2.37 (m, 1H), 2.33 – 2.23 (m, 1H), 2.23 – 2.15 (m, 1H), 2.14 – 2.02 (m, 2H), 2.00 – 1.88 (m, 1H), 1.88 – 1.78 (m, 2H), 1.76 – 1.39 (m, 7H), 1.33 – 1.21 (m, 2H), 1.12 – 0.94 (m, 5H), 0.89 (s, 9H), 0.88 (s, 3H), 0.06 (s, 6H). ^{13}C NMR (101 MHz, $CDCl_3$) δ 141.81, 120.38, 72.44, 51.83, 50.36, 47.55, 42.80, 37.33, 36.74, 35.84, 32.03, 31.55, 31.49, 30.84, 25.92, 21.89, 20.37, 19.45, 18.24, 13.55, -4.58.

(8R,9S,10R,13S,14S)-3-((tert-butyldimethylsilyl)oxy)-10,13-dimethyl-2,3,4,7,8,9,10,11,12,13,14,15-dodecahydro-1H-cyclopenta[a]phenanthren-17-yl trifluoromethanesulfonate

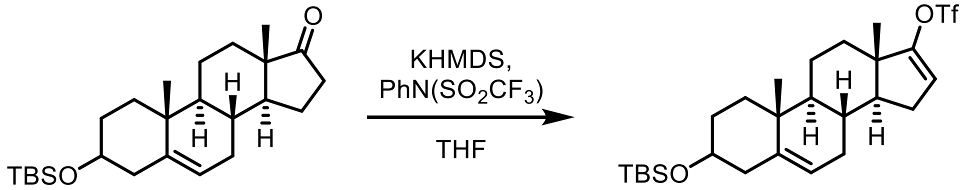

TBS protected dehydroepiandrosterone (3.624 g, 9.0 mmol) and *N*-Phenyl-bis(trifluoromethanesulfonimide) (3.537 g, 9.90 mmol) were dissolved in dry THF (90 mL) and cooled to -78 °C. KHMDS, 0.6 M in toluene (30.0 mL, 18.0 mmol), was added dropwise at −78 °C and the resulting mixture was stirred at -78 °C for 4 h. Then the reaction was brought to room temperature and quenched by addition of saturated aqueous NH_4Cl (30 mL). The mixture was extracted with CH_2Cl_2 (2 x 30 mL); the combined organic extracts were washed with water and brine, dried ($MgSO_4$) and concentrated *in vacuo*. The residue was purified by flash chromatography (silica gel, 10% ethyl acetate in hexane) to give the pure product as white solid in 89% yield 4.31 g. ^{1}H NMR (400 MHz, $CDCl_3$) δ 5.64 – 5.53 (m, 1H), 5.41 – 5.22 (m, 1H), 3.57 – 3.38 (m, 1H), 2.37 – 2.13 (m, 3H), 2.08 – 1.94 (m, 2H), 1.85 – 1.37 (m, 10H), 1.06 – 1.01 (m, 5H), 0.99 (s, 3H),

0.89 (s, 9H), 0.06 (s, 6H). ^{13}C NMR (101 MHz, $CDCl_3$) δ 159.44, 142.19, 120.44, 118.46 (q, J = 320.5 Hz), 114.65, 72.60, 54.53, 50.74, 44.85, 42.98, 37.39, 36.97, 32.90, 32.17, 30.76, 30.16, 28.78, 26.08, 20.33, 19.46, 18.39, 15.25, -4.42.

*General procedure for the Suzuki cross-coupling and synthesis of (**5a-e**)*

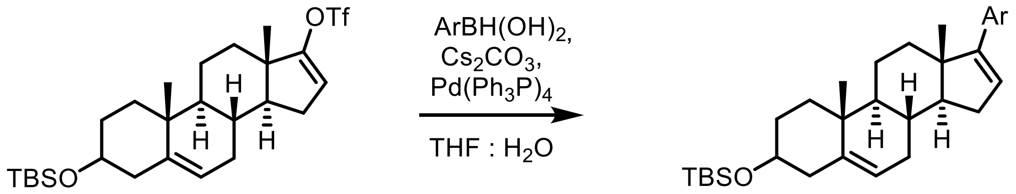

The TBS protected steroid triflate **4** (0.25-0.4 mmol, 1 equiv.), cesium carbonate (2 equiv.) and the boronic acid (1.05 equiv.) were placed in a flame dried 50 mL round-bottomed flask under argon atmosphere and dissolved in a 1:1 mixture of water and THF (10 mL). $Pd(PPh_3)_4$ (5 mol%) was added and the reaction mixture was stirred at room temperature (18 –22 h.). Upon completion the reaction mixture was poured into brine (15 mL) and extracted with ethyl acetate (4 x 5 mL). The combined organic extracts were dried ($MgSO_4$) and the solvent evaporated *in vacuo*. The residue was purified by flash chromatography (silica gel, 30% ethyl acetate in hexane) to give the pure products.

tert-butyl(((8R,9S,10R,13S,14S)-10,13-dimethyl-17-phenyl-2,3,4,7,8,9,10,11,12,13,14,15-dodecahydro-1H-cyclopenta[a]phenanthren-3-yl)oxy)dimethylsilane

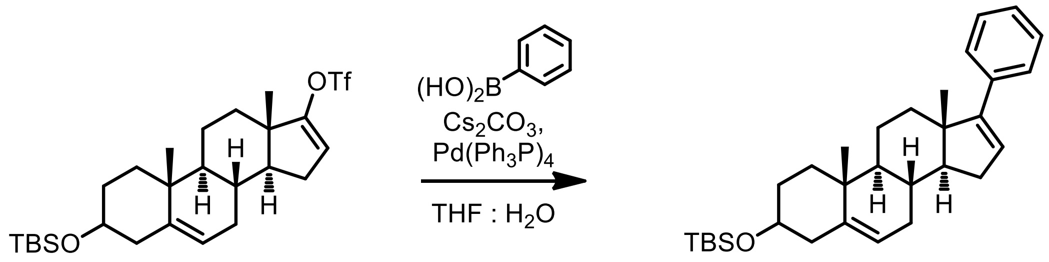

^{1}H NMR (400 MHz, $CDCl_3$) δ 7.41 – 7.35 (m, 2H), 7.33 – 7.27 (m, 2H), 7.25 – 7.19 (m, 1H), 5.95 – 5.88 (m, 1H), 5.40 – 5.34 (m, 1H), 3.56 – 3.45 (m, 1H), 2.35 – 2.17 (m, 3H), 2.14 – 1.98 (m, 3H), 1.86 – 1.42 (m, 9H), 1.13 – 1.00 (m, 8H), 0.90 (s, 9H), 0.07 (s, 6H). ^{13}C NMR (101 MHz, $CDCl_3$) δ 154.84, 141.92, 137.36, 128.06, 127.18, 126.69, 126.66, 120.93, 72.60, 57.75, 50.55, 47.24, 42.89, 37.34, 36.81, 35.45, 32.10, 31.63, 30.53, 25.95, 20.94, 19.38, 18.26, 16.63, -4.56. Yield 78%, 180 mg.

5-((8S,9S,13S,14S)-3-((tert-butyldimethylsilyl)oxy)-2-ethyl-13-methyl-7,8,9,11,12,13,14,15-octahydro-6H-cyclopenta[a]phenanthren-17-yl)isoquinoline

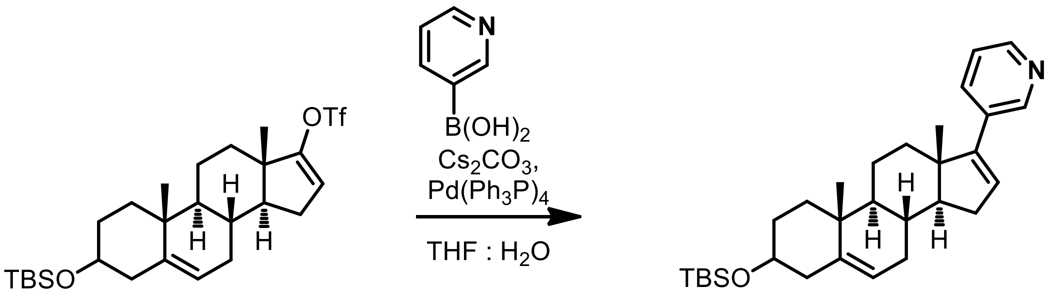

^{1}H NMR (400 MHz, $CDCl_3$) δ 8.62 (d, *J* = 2.2 Hz, 1H), 8.46 (d, *J* = 4.9 Hz, 1H), 7.66 (d, *J* = 8.0 Hz, 1H), 7.25 – 7.19 (m, 1H), 6.02 – 5.99 (m, 1H), 5.38 – 5.32 (m, 1H), 3.57 – 3.41 (m, 1H), 2.34 – 2.16 (m, 3H), 2.12 – 1.98 (m, 3H), 1.85 – 1.42 (m, 9H), 1.12 – 1.00 (m, 8H), 0.89 (s, 9H), 0.06 (s, 6H). ^{13}C NMR (101 MHz, $CDCl_3$) δ 151.81, 147.86, 147.75, 142.10, 134.07, 129.57, 123.23, 120.94, 72.71, 57.78, 50.63, 47.53, 43.01, 37.47, 36.95, 35.46, 32.23, 31.99, 31.72, 30.64, 26.10, 21.03, 19.53, 18.41, 16.73, -4.41. Yield 83%, 96 mg.

3-((3S,8R,9S,10R,13S,14S)-3-((tert-butyldimethylsilyl)oxy)-10,13-dimethyl-2,3,4,7,8,9,10,11,12,13,14,15-dodecahydro-1H-cyclopenta[a]phenanthren-17-yl)-5-chloropyridine

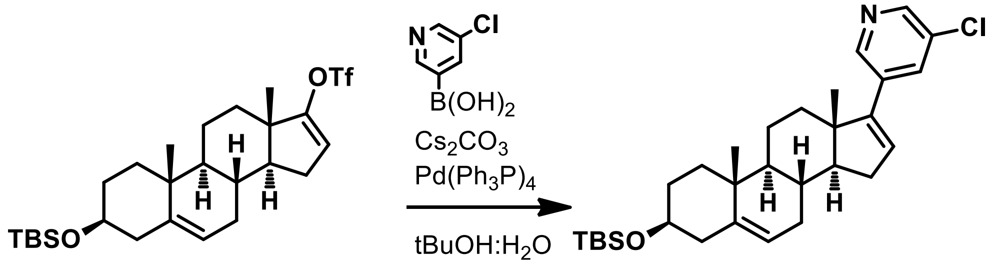

^{1}H NMR (400 MHz, $CDCl_3$) δ 8.26 (dd, *J* = 5.3, 0.7 Hz, 1H), 7.27 (dd, *J* = 1.4, 0.6 Hz, 1H), 7.17 (dd, *J* = 5.3, 1.5 Hz, 1H), 6.20 (dd, *J* = 3.4, 1.8 Hz, 1H), 5.34 (d, *J* = 5.3 Hz, 1H), 3.59 – 3.42 (m, 1H), 2.34 – 2.14 (m, 3H), 2.13 – 1.98 (m, 3H), 1.86 – 1.37 (m, 9H), 1.12 – 1.00 (m, 8H), 0.89 (s, 9H), 0.06 (s, 6H). ^{13}C NMR (101 MHz, $CDCl_3$) δ 151.83, 151.66, 149.47, 147.90, 142.07, 133.22, 121.46, 120.78, 120.04, 72.65, 57.71, 50.51, 47.36, 42.98, 37.42, 36.89, 35.22, 32.18, 32.04, 31.62, 30.50, 26.07, 20.94, 19.49, 18.38, 16.74, -4.43. Yield 67%, 83 mg.

4-((8R,9S,10R,13S,14S)-3-((tert-butyldimethylsilyl)oxy)-10,13-dimethyl-2,3,4,7,8,9,10,11,12,13,14,15-dodecahydro-1H-cyclopenta[a]phenanthren-17-yl)pyridine

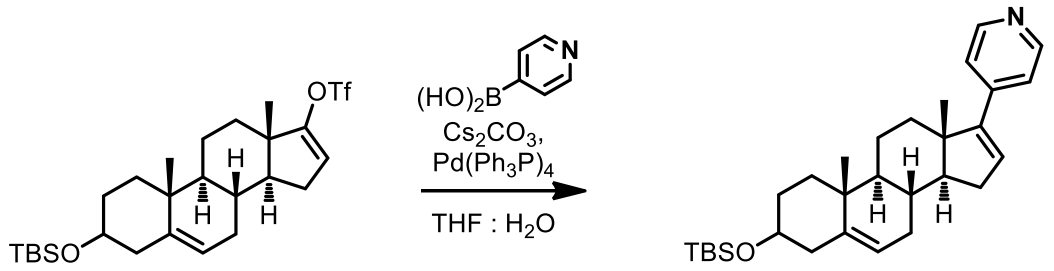

^{1}H NMR (400 MHz, $CDCl_3$) δ 8.43 (d, *J* = 6.2 Hz, 2H), 7.20 – 7.14 (m, 2H), 6.12 – 6.05 (m, 1H), 5.33 – 5.24 (m, 1H), 3.51 – 3.38 (m, 1H), 2.24 – 1.91 (m, 6H), 1.79 – 1.32 (m, 9H), 1.06 – 0.92 (m, 8H), 0.83 (s, 9H), 0.00 (s, 6H). ^{13}C NMR (101 MHz, $CDCl_3$) δ 152.51, 149.66, 144.48, 141.87, 131.35, 121.10, 120.71, 72.50, 57.58, 50.42, 47.13, 42.85, 37.28, 36.75, 35.17, 32.06, 31.81, 31.52, 30.38, 25.93, 20.84, 19.35, 18.21, 16.59, -4.56. Yield 86%, 100 mg.

4-((3S,8R,9S,10R,13S,14S)-3-((tert-butyldimethylsilyl)oxy)-10,13-dimethyl-2,3,4,7,8,9,10,11,12,13,14,15-dodecahydro-1H-cyclopenta[a]phenanthren-17-yl)-2-chloropyridine

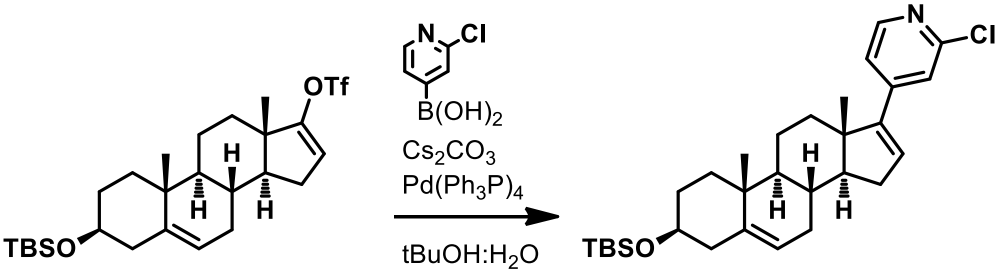

^{1}H NMR (400 MHz, $CDCl_3$) δ 8.43 (d, *J* = 1.9 Hz, 1H), 8.35 (d, *J* = 2.3 Hz, 1H), 7.56 (t, *J* = 2.1 Hz, 1H), 5.98 (dd, *J* = 3.3, 1.8 Hz, 1H), 5.29 (dd, *J* = 4.7, 2.4 Hz, 1H), 3.54 – 3.31 (m, 1H), 2.30 – 2.08 (m, 3H), 2.07 – 1.91 (m, 3H), 1.80 – 1.31 (m, 9H), 1.03 – 0.93 (m, 8H), 0.83 (s, 9H), -0.00 (s, 6H). ^{13}C NMR (101 MHz, $CDCl_3$) δ 150.47, 146.54, 145.62, 141.91, 134.15, 133.25, 131.56, 130.82, 120.70, 72.52, 57.58, 50.40, 47.40, 42.84, 37.29, 36.76, 35.21, 32.05, 31.88, 31.51, 30.43, 25.94, 20.83, 19.36, 18.24, 16.58, -4.56. Yield 53%, 105 mg.

4-((8R,9S,10R,13S,14S)-3-((tert-butyldimethylsilyl)oxy)-10,13-dimethyl-2,3,4,7,8,9,10,11,12,13,14,15-dodecahydro-1H-cyclopenta[a]phenanthren-17-yl)-1H-pyrazole

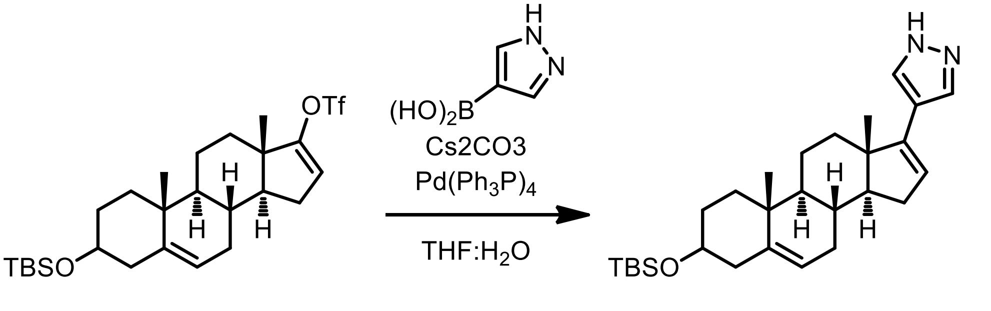

^{1}H NMR (400 MHz, $CDCl_3$) δ 7.63 (s, 2H), 5.90 – 5.70 (m, 1H), 5.46 – 5.24 (m, 1H), 3.60 – 3.31 (m, 1H), 2.37 – 2.15 (m, 3H), 2.10 – 1.92 (m, 3H), 1.87 – 1.45 (m, 9H), 1.06 (s, 5H), 0.95 (s, 3H), 0.89 (s, 10H), 0.06 (s, 6H). ^{13}C NMR (101 MHz, $CDCl_3$) δ 142.04, 124.17, 121.09, 100.16, 77.36, 72.75, 57.25, 50.83, 47.20, 43.03, 37.50, 36.97, 35.67, 32.26, 31.75, 31.66, 30.60, 29.86, 26.10, 21.13, 19.53, 18.42, 16.34, -4.41. Yield 81%, 92 mg.

4-((8R,9S,10R,13S,14S)-3-((tert-butyldimethylsilyl)oxy)-10,13-dimethyl-2,3,4,7,8,9,10,11,12,13,14,15-dodecahydro-1H-cyclopenta[a]phenanthren-17-yl)isoquinoline

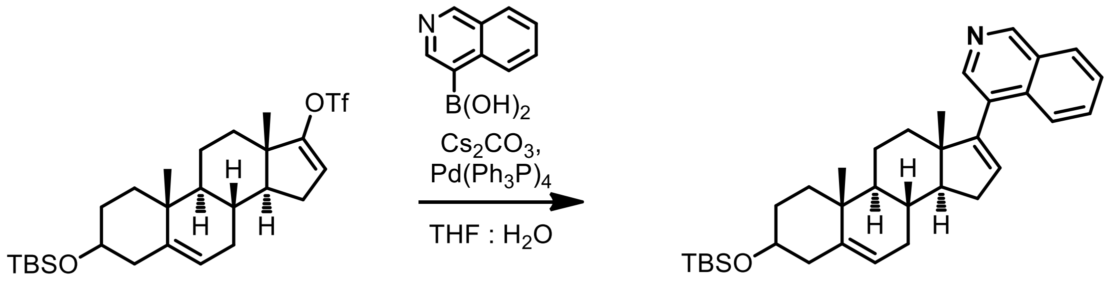

^{1}H NMR (400 MHz, $CDCl_3$) δ 9.16 (s, 1H), 8.34 (s, 1H), 8.06 – 7.92 (m, 2H), 7.72 – 7.54 (m, 2H), 5.91 – 5.79 (m, 1H), 5.43 – 5.33 (m, 1H), 3.59 – 3.43 (m, 1H), 2.48 – 2.38 (m, 1H), 2.34 – 2.08 (m, 4H), 1.89 – 1.42 (m, 10H), 1.16 – 0.96 (m, 8H), 0.90 (s, 9H), 0.07 (d, *J* = 1.1 Hz, 6H). ^{13}C NMR (101 MHz, $CDCl_3$) δ 151.29, 149.72, 142.15, 141.73, 135.76, 131.98, 130.19, 129.43, 128.60, 127.82, 127.10, 125.69, 120.96, 72.72, 57.67, 50.86, 49.76, 43.03, 37.48, 37.04, 35.28, 32.62, 32.22, 31.91, 31.09, 26.10, 21.00, 19.54, 18.40, 16.41, -4.40. Yield 87%, 112 mg.

5-((3S,8R,9S,10R,13S,14S)-3-((tert-butyldimethylsilyl)oxy)-10,13-dimethyl-2,3,4,7,8,9,10,11,12,13,14,15-dodecahydro-1H-cyclopenta[a]phenanthren-17-yl)isoquinoline

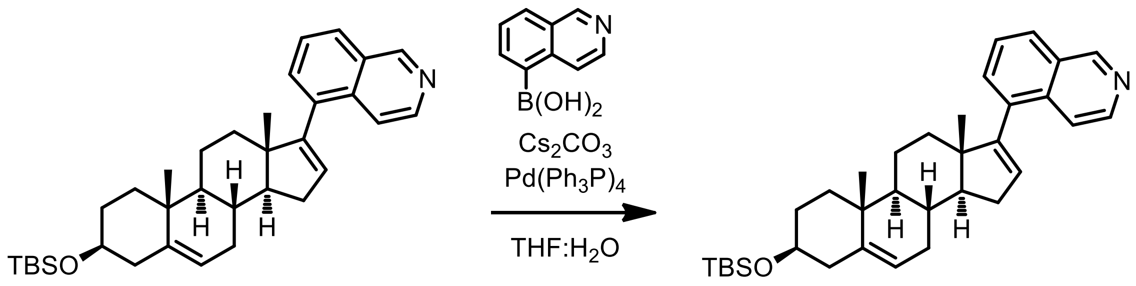

^{1}H NMR (400 MHz, $CDCl_3$) δ 9.23 (d, *J* = 0.9 Hz, 1H), 8.48 (d, *J* = 5.9 Hz, 1H), 7.92 – 7.83 (m, 2H), 7.61 – 7.54 (m, 1H), 7.49 (dd, *J* = 7.2, 1.3 Hz, 1H), 5.81 (dd, *J* = 3.1, 1.6 Hz, 1H), 5.50 – 5.24 (m, 1H), 3.65 – 3.34 (m, 1H), 2.42 (ddd, *J* = 15.4, 6.0, 3.0 Hz, 1H), 2.35 – 2.05 (m, 4H), 1.87 – 1.67 (m, 5H), 1.64 – 1.42 (m, 5H), 1.05 (s, 5H), 1.00 (s, 3H), 0.90 (s, 9H), 0.07 (d, *J* = 1.1 Hz, 6H). ^{13}C NMR (101 MHz, $CDCl_3$) δ 152.65, 151.29, 142.84, 142.12, 135.61, 135.24, 131.03, 129.70, 129.13, 126.67, 126.50, 120.98, 119.42, 72.71, 57.72, 50.86, 49.68, 43.02, 37.48, 37.02, 35.31, 32.52, 32.22, 31.90, 31.05, 26.10, 20.99, 19.53, 18.40, 16.49, -4.40. Yield 79%, 101 mg.

6-((3S,8R,9S,10R,13S,14S)-3-((tert-butyldimethylsilyl)oxy)-10,13-dimethyl-2,3,4,7,8,9,10,11,12,13,14,15-dodecahydro-1H-cyclopenta[a]phenanthren-17-yl)isoquinoline

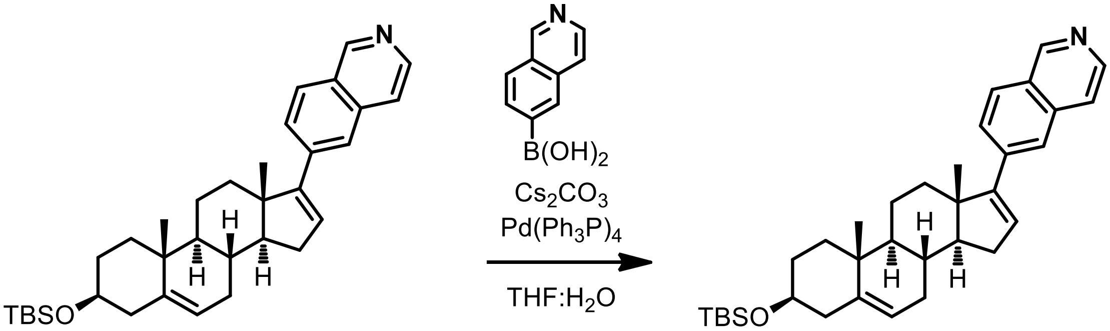

^{1}H NMR (400 MHz, $CDCl_3$) δ 9.19 (s, 1H), 8.49 (d, *J* = 5.8 Hz, 1H), 7.88 (d, *J* = 8.6 Hz, 1H), 7.77 (d, *J* = 1.7 Hz, 1H), 7.66 (dd, *J* = 8.6, 1.6 Hz, 1H), 7.61 (d, *J* = 5.7 Hz, 1H), 6.28 – 5.99 (m, 1H), 5.54 – 5.15 (m, 1H), 3.62 – 3.41 (m, 1H), 2.36 – 2.00 (m, 6H), 1.89 – 1.46 (m, 9H), 1.15 (s, 3H), 1.08 (s, 5H), 0.90 (s, 9H), 0.07 (s, 6H). ^{13}C NMR (101 MHz, $CDCl_3$) δ 154.13, 151.83, 142.95, 142.93, 141.95, 139.43, 136.00, 130.43, 127.54, 127.18, 122.90, 120.80, 120.64, 72.56, 57.78, 50.49, 47.47, 42.87, 37.33, 36.80, 35.49, 32.08, 31.90, 31.58, 30.50, 25.94, 20.95, 19.39, 18.25, 16.72, -4.57. Yield 60%, 77 mg.

7-((3S,8R,9S,10R,13S,14S)-3-((tert-butyldimethylsilyl)oxy)-10,13-dimethyl-2,3,4,7,8,9,10,11,12,13,14,15-dodecahydro-1H-cyclopenta[a]phenanthren-17-yl)isoquinoline

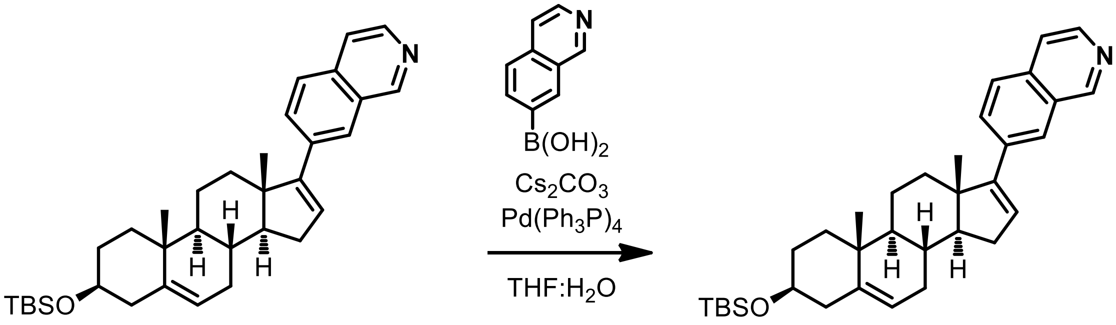

^{1}H NMR (400 MHz, $CDCl_3$) δ 9.21 (s, 1H), 8.47 (d, *J* = 5.7 Hz, 1H), 7.92 (s, 1H), 7.78 – 7.70 (m, 2H), 7.66 – 7.55 (m, 1H), 6.21 – 5.95 (m, 1H), 5.55 – 5.20 (m, 1H), 3.78 – 3.26 (m, 1H), 2.39 – 2.01 (m, 6H), 1.87 – 1.49 (m, 9H), 1.15 (s, 3H), 1.12 – 1.02 (m, 5H), 0.90 (s, 9H), 0.07 (s, 6H). ^{13}C NMR (101 MHz, $CDCl_3$) δ 153.98, 152.48, 142.50, 141.96, 136.35, 134.75, 130.28, 129.29, 128.78, 126.18, 124.00, 120.82, 120.18, 72.57, 57.78, 50.50, 47.44, 42.87, 37.33, 36.80, 35.53, 32.08, 31.81, 31.59, 30.51, 25.94, 20.96, 19.39, 18.26, 16.70, -4.57. Yield 63%, 81 mg.

4-((3S,10R,13S)-3-((tert-butyldimethylsilyl)oxy)-10,13-dimethyl-2,3,4,7,8,9,10,11,12,13,14,15-dodecahydro-1H-cyclopenta[a]phenanthren-17-yl)-1H-indole

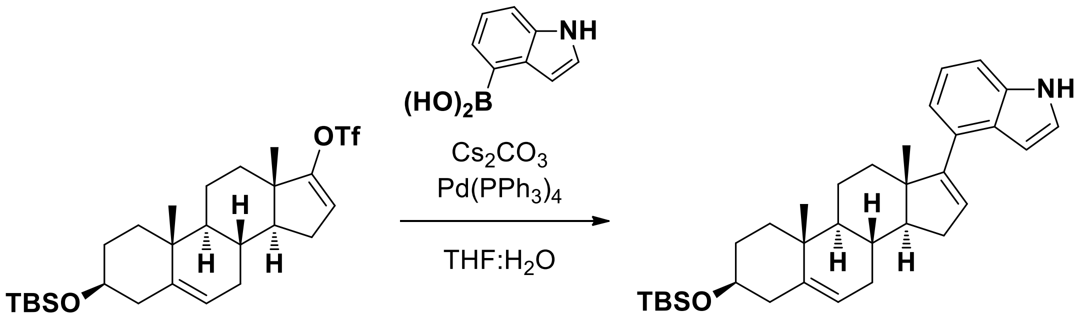

^{1}H NMR (400 MHz, CDCl$_3$) δ 8.18 (s, 1H), 7.30 – 7.27 (m, 1H), 7.22 – 7.10 (m, 2H), 7.04 (dd, *J* = 7.3, 1.0 Hz, 1H), 6.70 – 6.56 (m, 1H), 6.13 – 5.92 (m, 1H), 5.46 – 5.32 (m, 1H), 3.59 – 3.41 (m, 1H), 2.42 – 2.06 (m, 5H), 2.01 – 1.91 (m, 1H), 1.88 – 1.50 (m, 9H), 1.14 – 1.03 (m, 8H), 0.90 (s, 9H), 0.07 (s, 6H). ^{13}C NMR (101 MHz, CDCl$_3$) δ 153.52, 141.95, 136.01, 130.28, 128.53, 127.33, 123.58, 121.46, 121.01, 118.24, 109.57, 103.11, 72.64, 57.55, 50.73, 48.46, 42.90, 37.36, 36.86, 35.52, 32.11, 32.08, 31.74, 30.72, 25.96, 20.98, 19.41, 18.27, 16.83, -4.56. Yield 85%, 107 mg.

5-((3S,8R,9S,10R,13S,14S)-3-((tert-butyldimethylsilyl)oxy)-10,13-dimethyl-2,3,4,7,8,9,10,11,12,13,14,15-dodecahydro-1H-cyclopenta[a]phenanthren-17-yl)-1H-indole

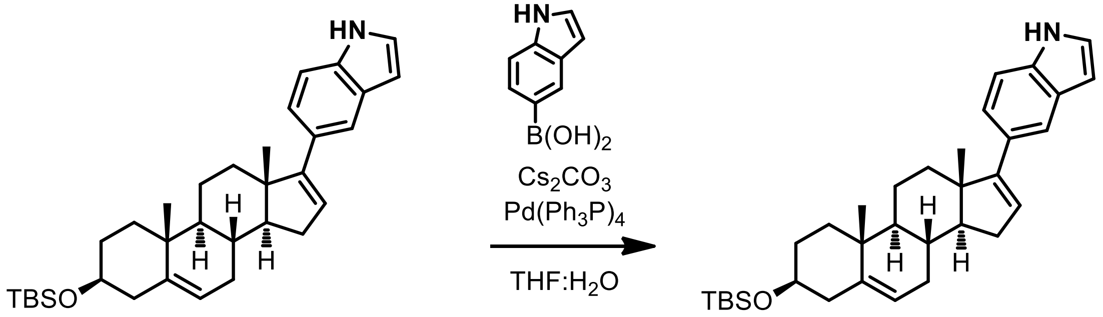

^{1}H NMR (400 MHz, CDCl$_3$) δ 8.10 (s, 1H), 7.65 (d, *J* = 1.8 Hz, 1H), 7.35 – 7.29 (m, 1H), 7.27 – 7.23 (m, 1H), 7.20 – 7.15 (m, 1H), 6.58 – 6.48 (m, 1H), 5.90 – 5.77 (m, 1H), 5.40 – 5.34 (m, 1H), 3.57 – 3.45 (m, 1H), 2.37 – 1.96 (m, 6H), 1.87 – 1.45 (m, 9H), 1.14 – 1.04 (m, 8H), 0.91 (s, 9H), 0.08 (s, 6H). ^{13}C NMR (101 MHz, CDCl$_3$) δ 155.95, 142.07, 135.09, 129.50, 127.92, 125.48, 124.45, 122.02, 121.19, 118.73, 110.71, 103.03, 72.81, 57.98, 50.78, 47.53, 43.06, 37.51, 36.99, 35.85, 32.28, 31.83, 31.74, 30.77, 26.12, 21.18, 19.56, 18.43, 16.84, -4.40. Yield 88%, 110 mg.

6-((3S,8R,9S,10R,13S,14S)-3-((tert-butyldimethylsilyl)oxy)-10,13-dimethyl-2,3,4,7,8,9,10,11,12,13,14,15-dodecahydro-1H-cyclopenta[a]phenanthren-17-yl)-1H-indole

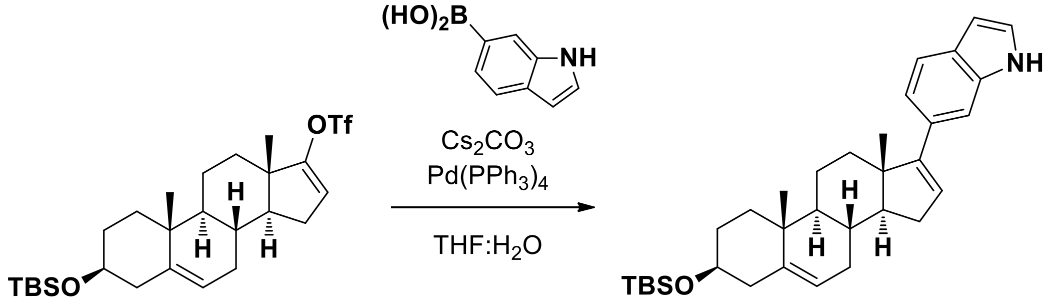

^{1}H NMR (400 MHz, $CDCl_3$) δ 8.09 (s, 1H), 7.65 – 7.50 (m, 1H), 7.45 – 7.35 (m, 1H), 7.23 – 7.10 (m, 2H), 6.58 – 6.47 (m, 1H), 5.99 – 5.84 (m, 1H), 5.43 – 5.29 (m, 1H), 3.60 – 3.41 (m, 1H), 2.44 – 1.96 (m, 6H), 1.92 – 1.43 (m, 9H), 1.12 – 1.02 (m, 8H), 0.92 (s, 9H), 0.08 (s, 6H). ^{13}C NMR (101 MHz, $CDCl_3$) δ 155.65, 141.91, 135.92, 131.53, 126.77, 125.99, 124.26, 121.02, 120.16, 119.66, 108.89, 102.55, 72.65, 57.82, 50.61, 47.38, 42.91, 37.36, 36.83, 35.71, 32.12, 31.66, 31.62, 30.60, 25.97, 21.04, 19.41, 18.28, 16.77, -4.54. Yield 76%, 114 mg.

A general procedure for the deprotection

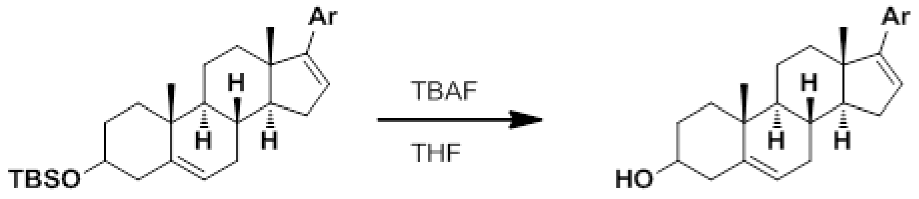

The TBS protected steroids **5a-m** (0.15-0.2 mmol, 1 equiv.) were placed in a dry round-bottomed flask under argon atmosphere, and dissolved in dry THF (3-4 mL). *Tert*-butylamoniumfluoride (1 M in THF, 1.1 equiv.) was added dropvise. The reaction mixture was stirred at room temperature (16-18 h.). Upon completion the reaction the mixture was poured into saturated aqueous $NaHCO_3$ (10 mL), and extracted with ethyl acetate (4 x 5 mL). The combined organic extracts were dried ($MgSO_4$) and the solvent evaporated *in vacuo*. The residues were purified by chromatography (silica gel, 20-50% ethyl acetate in hexane) to give the pure products.

(8R,9S,10R,13S,14S)-10,13-dimethyl-17-phenyl-2,3,4,7,8,9,10,11,12,13,14,15-dodecahydro-1H-cyclopenta[a]phenanthren-3-ol

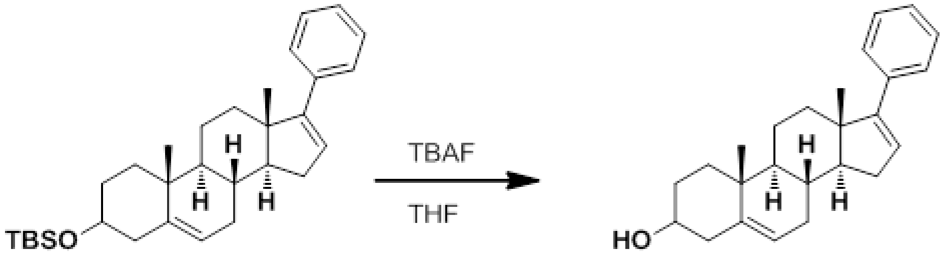

Colourless solid (61 mg, 87%). Purified by column chromatography on silica gel using 20% ethyl acetate in heptane as eluent. R_f = 0.43 (20% ethyl acetate in heptane, CAM-stain). ^{1}H NMR (400 MHz, $CDCl_3$) δ 7.37 – 7.32 (m, 2H), 7.29 – 7.15 (m, 3H), 6.00 – 5.68 (m, 1H), 5.50 – 5.22 (m, 1H), 3.64 – 3.36 (m, 1H), 2.37 – 2.14 (m, 3H), 2.12 – 1.95 (m, 3H), 1.88 – 1.38 (m, 9H), 1.12 – 0.99 (m, 8H), 0.89 – 0.82 (m, 1H). ^{13}C NMR (101 MHz, $CDCl_3$) δ 154.95, 141.27, 137.48, 128.22, 127.32, 126.83, 121.61, 71.90, 57.84, 50.61, 47.37, 42.49, 37.35, 36.87, 35.57, 31.81, 31.76, 31.73, 30.66, 21.10, 19.49, 16.78.

(3S,8R,9S,10R,13S,14S)-10,13-dimethyl-17-(pyridin-3-yl)-2,3,4,7,8,9,10,11,12,13,14,15-dodecahydro-1H-cyclopenta[a]phenanthren-3-ol

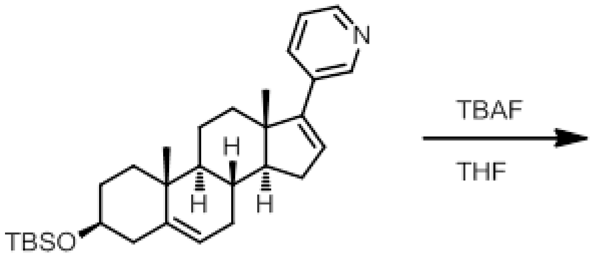

Colourless solid (62 mg, 89%). Purified by column chromatography on silica gel using 50% ethyl acetate in heptane as eluent. R_f = 0.21 (50% ethyl acetate in heptane, CAM-stain). ^{1}H NMR (400 MHz, $CDCl_3$) δ 8.61 (d, *J* = 2.5 Hz, 1H), 8.45 (dd, *J* = 4.8, 1.7 Hz, 1H), 7.64 (dt, *J* = 7.9, 1.9 Hz, 1H), 7.25 – 7.17 (m, 1H), 5.99 (dd, *J* = 3.3, 1.8 Hz, 1H), 5.52 – 5.27 (m, 1H), 3.60 – 3.48 (m, 1H), 2.38 – 2.20 (m, 3H), 2.15 – 2.00 (m, 3H), 1.90 – 1.41 (m, 9H), 1.15 – 1.01 (m, 8H). ^{13}C NMR (101 MHz, $CDCl_3$) δ 151.81, 147.95, 147.85, 141.34, 133.90, 133.17, 129.42, 123.18, 121.42, 71.79, 57.71, 50.53, 47.50, 42.47, 37.34, 36.85, 35.42, 31.96, 31.79, 31.67, 30.61, 21.03, 19.48, 16.72.

(3S,8R,9S,10R,13S,14S)-17-(5-chloropyridin-3-yl)-10,13-dimethyl-2,3,4,7,8,9,10,11,12,13,14,15-dodecahydro-1H-cyclopenta[a]phenanthren-3-ol

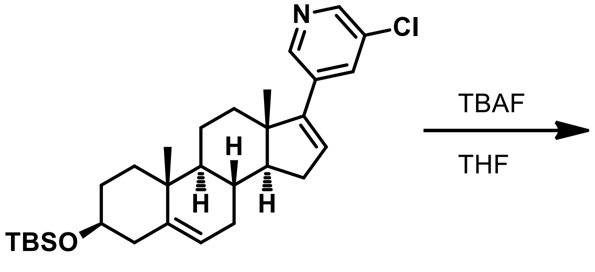

Colourless solid (56 mg, 73%). Purified by column chromatography on silica gel using 50% ethyl acetate in heptane as eluent. R_f = 0.48 (50% ethyl acetate in heptane, CAM-stain). ^{1}H NMR (400 MHz, $CDCl_3$) δ 8.49 (d, *J* = 1.9 Hz, 1H), 8.42 (d, *J* = 2.3 Hz, 1H), 7.64 (t, *J* = 2.1 Hz, 1H), 6.05 (dd, *J* = 3.3, 1.8 Hz, 1H), 5.39 (d, *J* = 5.2 Hz, 1H), 3.76 – 3.45 (m, 1H), 2.38 – 2.21 (m, 3H), 2.13

– 1.99 (m, 3H), 1.89 – 1.81 (m, 2H), 1.81 – 1.42 (m, 8H), 1.15 – 1.08 (m, 1H), 1.07 (s, 3H), 1.04 (s, 3H). ^{13}C NMR (101 MHz, $CDCl_3$) δ 150.39, 146.43, 145.50, 141.15, 134.21, 133.40, 131.63, 130.95, 121.25, 71.67, 57.53, 50.30, 47.39, 42.28, 37.16, 36.69, 35.17, 31.88, 31.62, 31.47, 30.41, 20.84, 19.34, 16.60.

(3S,8R,9S,10R,13S,14S)-10,13-dimethyl-17-(pyridin-4-yl)-2,3,4,7,8,9,10,11,12,13,14,15-dodecahydro-1H-cyclopenta[a]phenanthren-3-ol

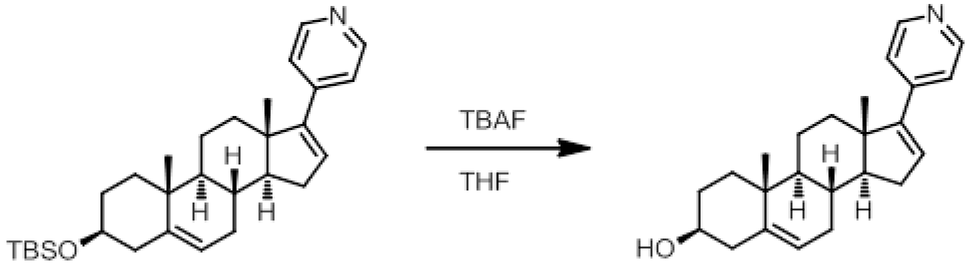

Colourless solid (62 mg, 88%). Purified by column chromatography on silica gel using 50% ethyl acetate in heptane as eluent. R_f = 0.17 (50% ethyl acetate in heptane, CAM-stain).^{1}H NMR (400 MHz, $CDCl_3$) δ 8.50 (d, *J* = 5.0 Hz, 2H), 7.26 (dd, *J* = 4.6, 1.6 Hz, 2H), 6.18 (dd, *J* = 3.3, 1.8 Hz, 1H), 5.42 – 5.36 (m, 1H), 3.61 – 3.47 (m, 1H), 2.38 – 2.20 (m, 3H), 2.17 – 2.00 (m, 3H), 1.90 – 1.40 (m, 9H), 1.17 – 1.00 (m, 8H). ^{13}C NMR (101 MHz, $CDCl_3$) δ 152.61, 149.57, 144.94, 141.34, 131.83, 121.39, 121.36, 71.82, 57.71, 50.50, 47.31, 42.46, 37.33, 36.85, 35.31, 32.00, 31.79, 31.64, 30.54, 21.01, 19.49, 16.77.

(3S,8R,9S,10R,13S,14S)-17-(2-chloropyridin-4-yl)-10,13-dimethyl-2,3,4,7,8,9,10,11,12,13,14,15-dodecahydro-1H-cyclopenta[a]phenanthren-3-ol

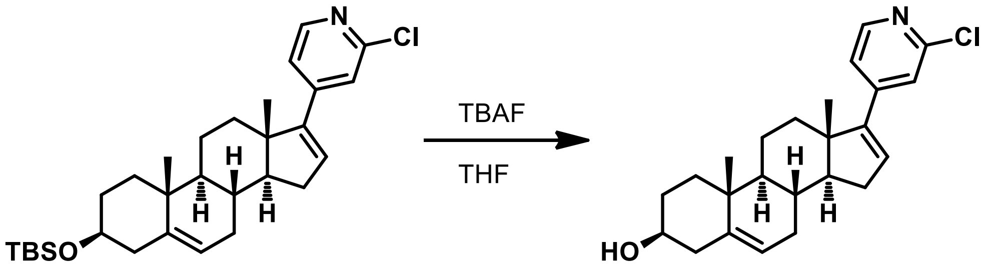

Colourless solid (50 mg, 65%). Purified by column chromatography on silica gel using 50% ethyl acetate in heptane as eluent. R_f = 0.58 (50% ethyl acetate in heptane, CAM-stain). ^{1}H NMR (400 MHz, $CDCl_3$) δ 8.27 (d, *J* = 5.2 Hz, 1H), 7.29 (d, *J* = 1.4 Hz, 1H), 7.18 (dd, *J* = 5.2, 1.5 Hz, 1H), 6.22 (dd, *J* = 3.3, 1.9 Hz, 1H), 5.39 (dt, *J* = 5.2, 2.0 Hz, 1H), 3.54 (tt, *J* = 11.3, 4.6 Hz, 1H), 2.39 – 2.20 (m, 3H), 2.14 – 2.00 (m, 3H), 1.90 – 1.81 (m, 2H), 1.80 – 1.39 (m, 8H), 1.17 – 1.01 (m, 7H). ^{13}C NMR (101 MHz, $CDCl_3$) δ 151.65, 151.47, 149.31, 147.79, 141.16, 133.17, 121.33, 121.20,

119.92, 71.66, 57.51, 50.27, 47.20, 42.27, 37.15, 36.68, 35.04, 31.91, 31.61, 31.44, 30.34, 20.81, 19.33, 16.62.

(3S,8R,9S,10R,13S,14S)-10,13-dimethyl-17-(1H-pyrazol-4-yl)-2,3,4,7,8,9,10,11,12,13,14,15-dodecahydro-1H-cyclopenta[a]phenanthren-3-ol

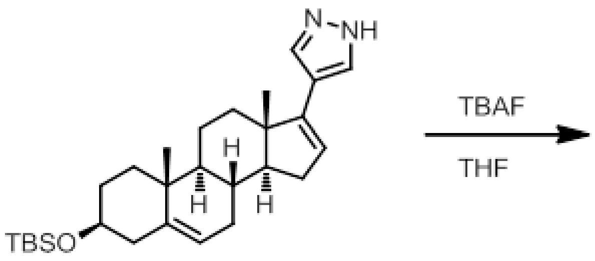

Colourless solid (28 mg, 41%). Purified by column chromatography on silica gel using 50% ethyl acetate in heptane as eluent. R_f = 0.16 (50% ethyl acetate in heptane, CAM-stain). ^{1}H NMR (400 MHz, DMSO) δ 12.69 (s, 1H), 7.66 (s, 2H), 5.76 (dd, *J* = 3.1, 1.7 Hz, 1H), 5.50 – 5.10 (m, 1H), 4.59 (d, *J* = 4.5 Hz, 1H), 3.28 – 3.14 (m, 1H), 2.23 – 2.05 (m, 4H), 2.05 – 1.88 (m, 2H), 1.83 – 1.29 (m, 9H), 1.06 – 0.94 (m, 5H), 0.90 (s, 3H). ^{13}C NMR (101 MHz, DMSO) δ 145.95, 141.57, 121.66, 120.26, 116.06, 69.98, 56.61, 50.08, 46.33, 42.25, 40.15, 36.83, 36.27, 34.72, 31.41, 31.03, 30.90, 29.96, 20.53, 19.06, 15.92.

(3S,8R,9S,10R,13S,14S)-17-(isoquinolin-4-yl)-10,13-dimethyl-2,3,4,7,8,9,10,11,12,13,14,15-dodecahydro-1H-cyclopenta[a]phenanthren-3-ol

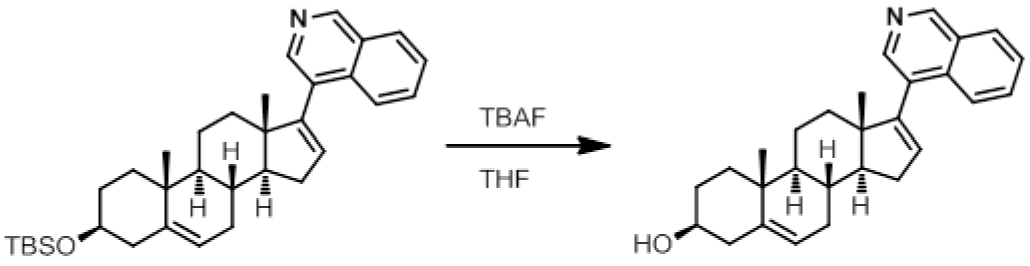

Colourless solid (64 mg, 80%). Purified by column chromatography on silica gel using 50% ethyl acetate in heptane as eluent. R_f = 0.27 (50% ethyl acetate in heptane, CAM-stain). ^{1}H NMR (400 MHz, $CDCl_3$) δ 9.16 (d, *J* = 0.9 Hz, 1H), 8.33 (s, 1H), 8.03 (dd, *J* = 8.5, 1.1 Hz, 1H), 7.97 (dt, *J* = 8.0, 1.1 Hz, 1H), 7.73 – 7.63 (m, 1H), 7.65 – 7.55 (m, 1H), 5.87 (dd, *J* = 3.1, 1.6 Hz, 1H), 5.50 – 5.34 (m, 1H), 3.68 – 3.42 (m, 1H), 2.48 – 2.37 (m, 1H), 2.40 – 2.18 (m, 3H), 2.19 – 2.09 (m, 1H), 1.90 – 1.71 (m, 5H), 1.63 – 1.43 (m, 5H), 1.17 – 1.03 (m, 5H), 1.01 (s, 3H). ^{13}C NMR (101 MHz, $CDCl_3$) δ 151.16, 149.65, 141.48, 141.38, 135.81, 132.05, 130.31, 129.54, 128.58, 127.87, 127.18, 125.69, 121.49, 71.87, 57.65, 50.78, 49.75, 42.48, 37.37, 36.94, 35.24, 32.62, 31.86, 31.81, 31.07, 21.01, 19.50, 16.42.

(3S,8R,9S,10R,13S,14S)-17-(isoquinolin-5-yl)-10,13-dimethyl-2,3,4,7,8,9,10,11,12,13,14,15-dodecahydro-1H-cyclopenta[a]phenanthren-3-ol

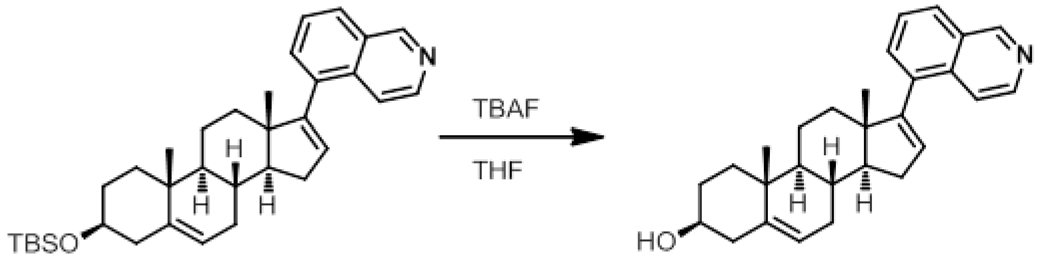

Colourless solid (62 mg, 77%). Purified by column chromatography on silica gel using 50% ethyl acetate in heptane as eluent. R_f = 0.29 (50% ethyl acetate in heptane, CAM-stain). ^{1}H NMR (400 MHz, $CDCl_3$) δ 9.21 (s, 1H), 8.47 (d, *J* = 5.9 Hz, 1H), 7.90 – 7.81 (m, 2H), 7.59 – 7.53 (m, 1H), 7.48 (dd, *J* = 7.2, 1.3 Hz, 1H), 5.79 (dd, *J* = 3.1, 1.6 Hz, 1H), 5.55 – 5.24 (m, 1H), 3.77 – 3.36 (m, 1H), 2.45 – 2.07 (m, 5H), 1.89 – 1.68 (m, 5H), 1.62 – 1.43 (m, 5H), 1.16 – 1.03 (m, 5H), 0.99 (s, 3H). ^{13}C NMR (101 MHz, $CDCl_3$) δ 152.66, 151.24, 142.88, 141.39, 135.54, 135.15, 130.95, 129.61, 129.11, 126.63, 126.44, 121.40, 119.35, 71.72, 57.64, 50.74, 49.62, 42.46, 37.34, 36.90, 35.24, 32.47, 31.82, 31.76, 30.99, 20.96, 19.47, 16.46.

(3S,8R,9S,10R,13S,14S)-17-(isoquinolin-6-yl)-10,13-dimethyl-2,3,4,7,8,9,10,11,12,13,14,15-dodecahydro-1H-cyclopenta[a]phenanthren-3-ol

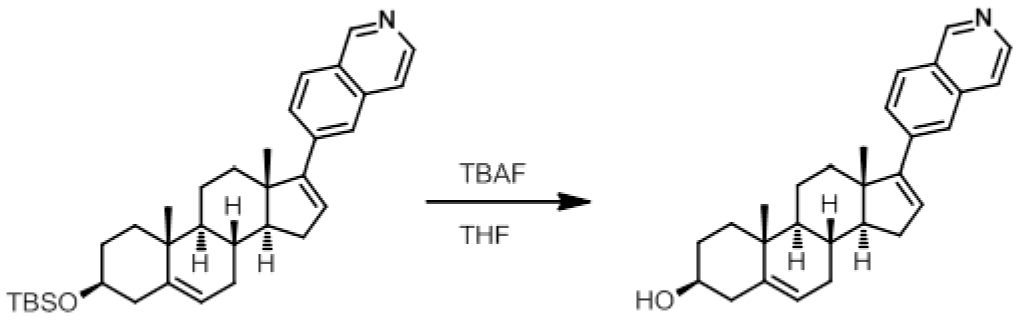

Colourless solid (52 mg, 87%). Purified by column chromatography on silica gel using 50% ethyl acetate in heptane as eluent. R_f = 0.24 (50% ethyl acetate in heptane, CAM-stain). ^{1}H NMR (400 MHz, $CDCl_3$) δ 9.19 (s, 1H), 8.49 (d, *J* = 5.8 Hz, 1H), 7.88 (dd, *J* = 8.4, 0.9 Hz, 1H), 7.80 – 7.74 (m, 1H), 7.66 (dd, *J* = 8.6, 1.6 Hz, 1H), 7.64 – 7.57 (m, 1H), 6.16 (dd, *J* = 3.3, 1.8 Hz, 1H), 5.44 – 5.37 (m, 1H), 3.62 – 3.48 (m, 1H), 2.38 – 2.24 (m, 3H), 2.21 (dt, *J* = 12.2, 3.6 Hz, 1H), 2.17 – 2.04 (m, 2H), 1.92 – 1.45 (m, 9H), 1.15 (s, 5H), 1.09 (s, 3H). ^{13}C NMR (101 MHz, $CDCl_3$) δ 154.26, 152.07, 143.22, 141.32, 139.49, 136.13, 130.53, 127.72, 127.42, 127.30, 123.05, 121.50, 120.77,

71.86, 57.88, 50.55, 47.61, 42.48, 37.35, 36.87, 35.62, 32.05, 31.81, 31.70, 30.65, 21.12, 19.51, 16.89.

(3S,8R,9S,10R,13S,14S)-17-(isoquinolin-7-yl)-10,13-dimethyl-2,3,4,7,8,9,10,11,12,13,14,15-dodecahydro-1H-cyclopenta[a]phenanthren-3-ol

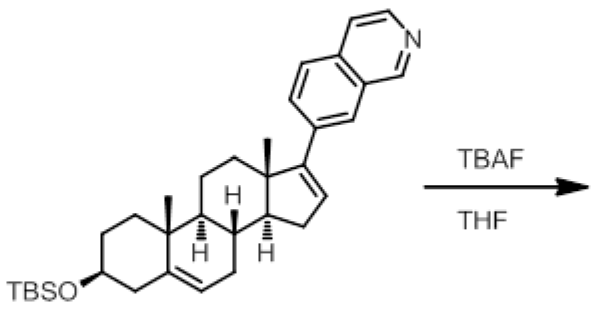

Colourless solid (50 mg, 83%). Purified by column chromatography on silica gel using 50% ethyl acetate in heptane as eluent. R_f = 0.26 (50% ethyl acetate in heptane, CAM-stain). ^{1}H NMR (400 MHz, DMSO) δ 9.32 (s, 1H), 8.44 (d, *J* = 5.6 Hz, 1H), 8.08 (d, *J* = 1.8 Hz, 1H), 7.93 – 7.82 (m, 2H), 7.81 – 7.72 (m, 1H), 6.33 – 6.15 (m, 1H), 5.41 – 5.21 (m, 1H), 4.60 (d, *J* = 4.5 Hz, 1H), 3.29 – 3.22 (m, 1H), 2.36 – 1.99 (m, 6H), 1.85 – 1.50 (m, 7H), 1.49 – 1.32 (m, 2H), 1.14 (s, 3H), 1.06 – 0.95 (m, 5H). ^{13}C NMR (101 MHz, DMSO) δ 153.18, 152.54, 142.61, 141.61, 135.26, 134.05, 129.80, 129.13, 128.36, 126.36, 123.32, 120.23, 119.90, 69.98, 57.21, 49.84, 46.67, 42.24, 36.83, 36.26, 34.66, 31.40, 31.27, 30.96, 29.97, 20.52, 19.06, 16.27.

(3S,8R,9S,10R,13S,14S)-17-(1H-indol-4-yl)-10,13-dimethyl-2,3,4,7,8,9,10,11,12,13,14,15-dodecahydro-1H-cyclopenta[a]phenanthren-3-ol

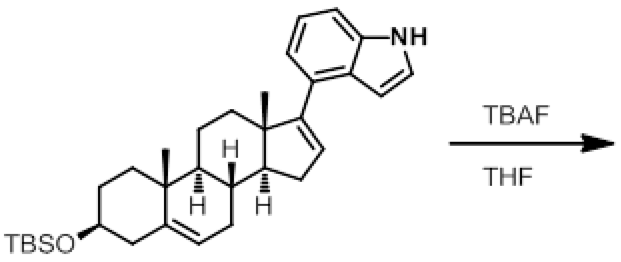

Colourless solid (60 mg, 78%). Purified by column chromatography on silica gel using 50% ethyl acetate in heptane as eluent. R_f = 0.21 (50% ethyl acetate in heptane, CAM-stain). ^{1}H NMR (400 MHz, DMSO) δ 11.05 (s, 1H), 7.32 – 7.21 (m, 2H), 7.02 (t, *J* = 7.7 Hz, 1H), 6.90 (dd, *J* = 7.4, 1.0 Hz, 1H), 6.49 – 6.43 (m, 1H), 5.96 (dd, *J* = 3.1, 1.7 Hz, 1H), 5.33 (d, *J* = 5.1 Hz, 1H), 4.59 (d, *J* = 4.5 Hz, 1H), 3.30 – 3.22 (m, 1H), 2.34 – 2.26 (m, 1H), 2.23 – 2.00 (m, 4H), 1.95 – 1.88 (m, 1H), 1.80 – 1.29 (m, 9H), 1.02 (d, *J* = 9.7 Hz, 8H). ^{13}C NMR (101 MHz, DMSO) δ 153.28, 141.59,

136.06, 128.78, 127.49, 126.77, 124.76, 120.40, 120.30, 116.87, 110.07, 101.27, 69.98, 57.00, 50.07, 47.71, 42.26, 36.86, 36.30, 35.08, 31.54, 31.42, 31.10, 30.18, 20.51, 19.09, 16.69.

(3S,8R,9S,10R,13S,14S)-17-(1H-indol-5-yl)-10,13-dimethyl-2,3,4,7,8,9,10,11,12,13,14,15-dodecahydro-1H-cyclopenta[a]phenanthren-3-ol

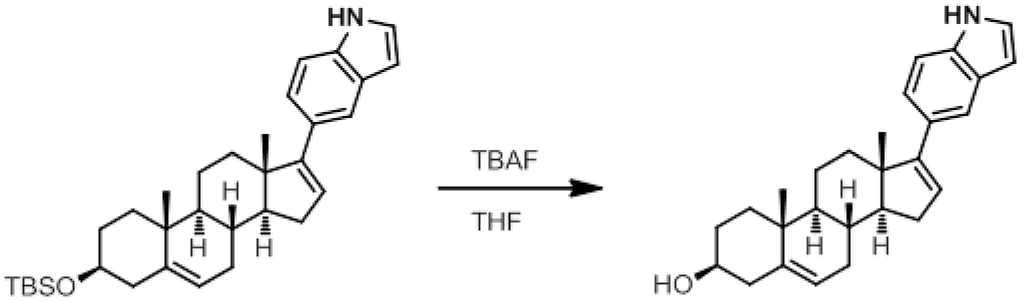

Colourless solid (56 mg, 72%). Purified by column chromatography on silica gel using 50% ethyl acetate in heptane as eluent. R_f = 0.18 (50% ethyl acetate in heptane, CAM-stain). ^{1}H NMR (400 MHz, DMSO) δ 11.00 (s, 1H), 7.52 (d, *J* = 1.9 Hz, 1H), 7.37 – 7.25 (m, 2H), 7.13 (dd, *J* = 8.4, 1.7 Hz, 1H), 6.45 – 6.34 (m, 1H), 5.81 (dd, *J* = 3.0, 1.6 Hz, 1H), 5.32 (d, *J* = 4.8 Hz, 1H), 4.65 – 4.53 (m, 1H), 3.29 – 3.22 (m, 1H), 2.23 – 2.10 (m, 4H), 2.06 – 1.94 (m, 2H), 1.82 – 1.30 (m, 9H), 1.08 – 0.95 (m, 8H). ^{13}C NMR (101 MHz, DMSO) δ 155.31, 141.58, 134.96, 127.51, 125.38, 123.98, 120.37, 120.27, 117.46, 111.05, 101.28, 69.98, 57.30, 49.95, 46.64, 42.25, 36.84, 36.26, 35.17, 31.40, 31.03, 31.00, 30.07, 20.53, 19.06, 16.50.

(3S,8R,9S,10R,13S,14S)-17-(1H-indol-6-yl)-10,13-dimethyl-2,3,4,7,8,9,10,11,12,13,14,15-dodecahydro-1H-cyclopenta[a]phenanthren-3-ol

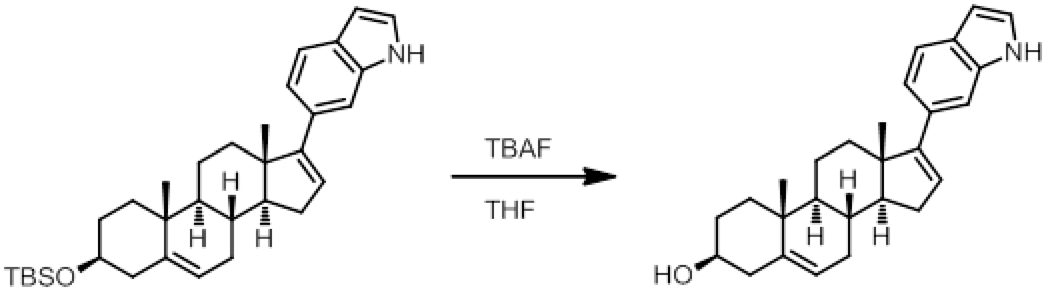

Colourless solid (53 mg, 69%). Purified by column chromatography on silica gel using 50% ethyl acetate in heptane as eluent. R_f = 0.25 (50% ethyl acetate in heptane, CAM-stain). ^{1}H NMR (400 MHz, DMSO) δ 10.91 (s, 1H), 7.41 (d, *J* = 8.3 Hz, 1H), 7.35 (s, 1H), 7.26 (t, *J* = 2.7 Hz, 1H), 7.02 (dd, *J* = 8.3, 1.5 Hz, 1H), 6.45 – 6.17 (m, 1H), 5.92 – 5.73 (m, 1H), 5.42 – 5.12 (m, 1H), 4.54 (s, 1H), 3.26 – 3.18 (m, 1H), 2.21 – 2.06 (m, 4H), 2.03 – 1.93 (m, 2H), 1.79 – 1.26 (m, 9H), 1.06 – 0.91 (m, 8H). ^{13}C NMR (101 MHz, DMSO) δ 155.05, 141.59, 135.93, 129.50, 126.49, 125.45,

124.72, 120.27, 119.59, 118.25, 108.80, 100.85, 69.98, 57.27, 49.93, 46.67, 42.25, 36.84, 36.27, 35.21, 31.41, 31.03, 30.06, 20.53, 19.07, 16.54.

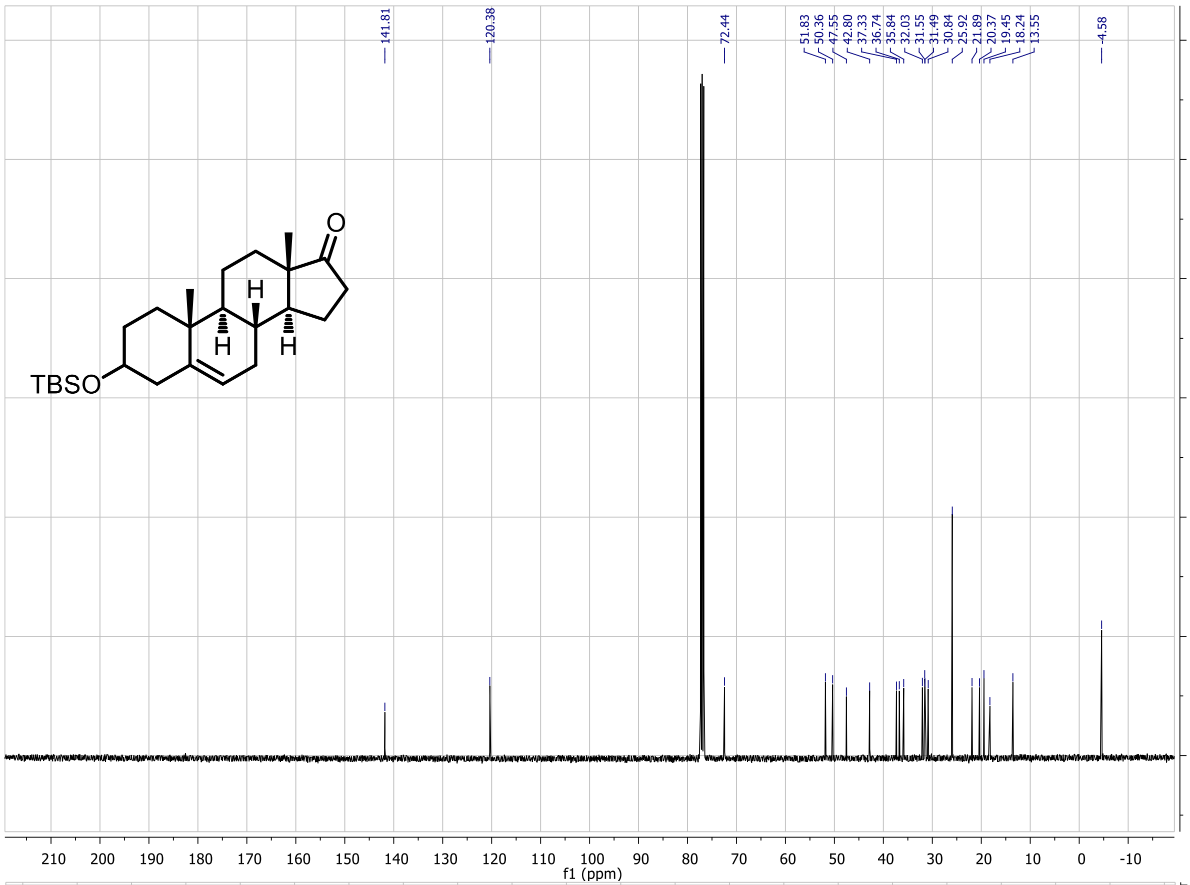

141.81
120.38
72.44
51.83
50.36
47.55
42.80
37.33
36.74
35.84
32.03
31.55
31.49
30.84
25.92
21.89
20.37
19.45
18.24
13.55
-4.58
O
H
H
H
TBSO
f1 (ppm)

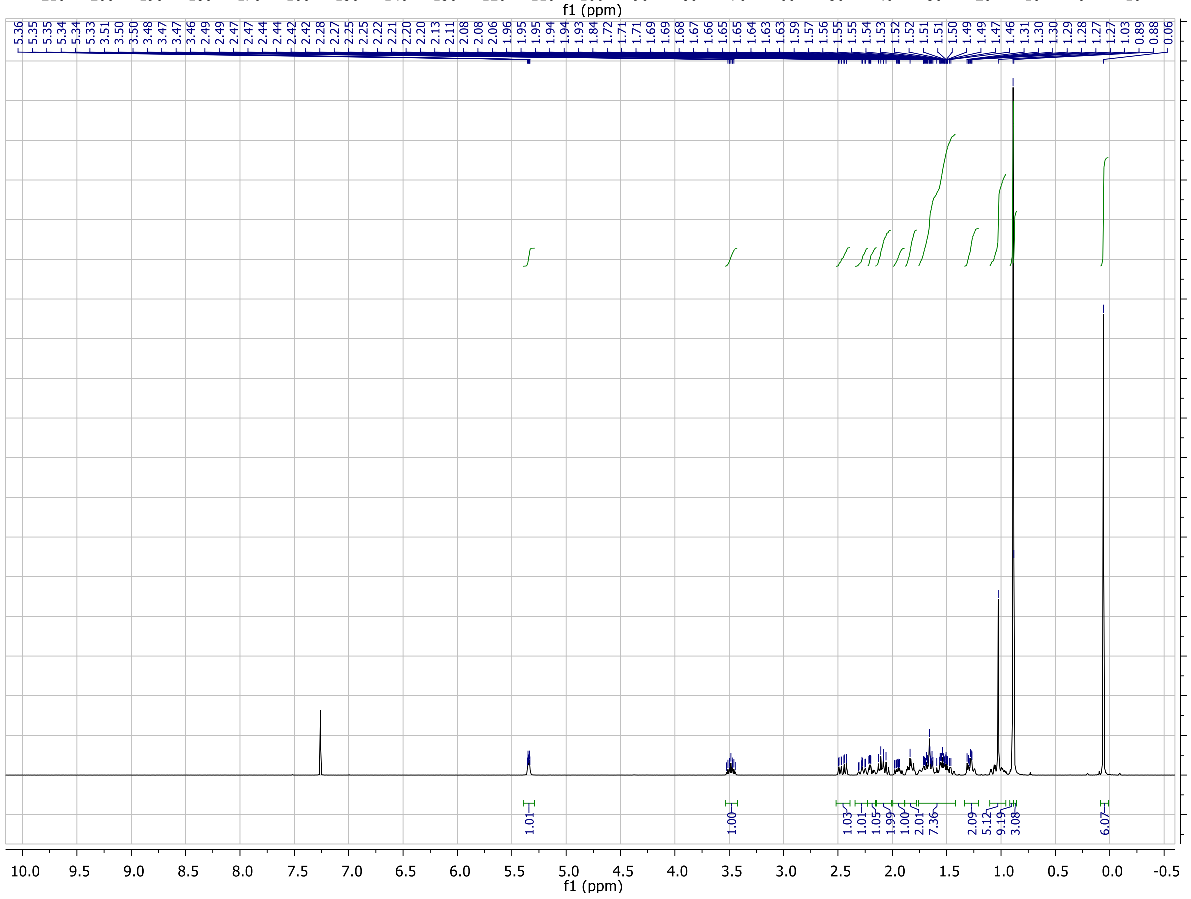

5.36 5.35 5.35 5.34 5.34 5.33 3.51 3.50 3.50 3.48 3.47 3.46 2.49 2.49 2.47 2.47 2.44 2.44 2.42 2.42 2.28 2.27 2.25 2.22 2.22 2.21 2.20 2.20 2.13 2.11 2.08 2.08 2.06 1.96 1.95 1.95 1.94 1.94 1.93 1.84 1.72 1.71 1.71 1.69 1.69 1.68 1.67 1.66 1.65 1.65 1.64 1.63 1.63 1.59 1.57 1.56 1.55 1.55 1.54 1.53 1.52 1.52 1.51 1.51 1.50 1.49 1.49 1.47 1.46 1.31 1.30 1.29 1.28 1.27 1.27 1.03 0.89 0.88 0.06
1.01
1.00
1.03
1.01
1.05
1.99
1.00
2.01
7.36
2.09
5.12
9.19
3.08
6.07
f1 (ppm)

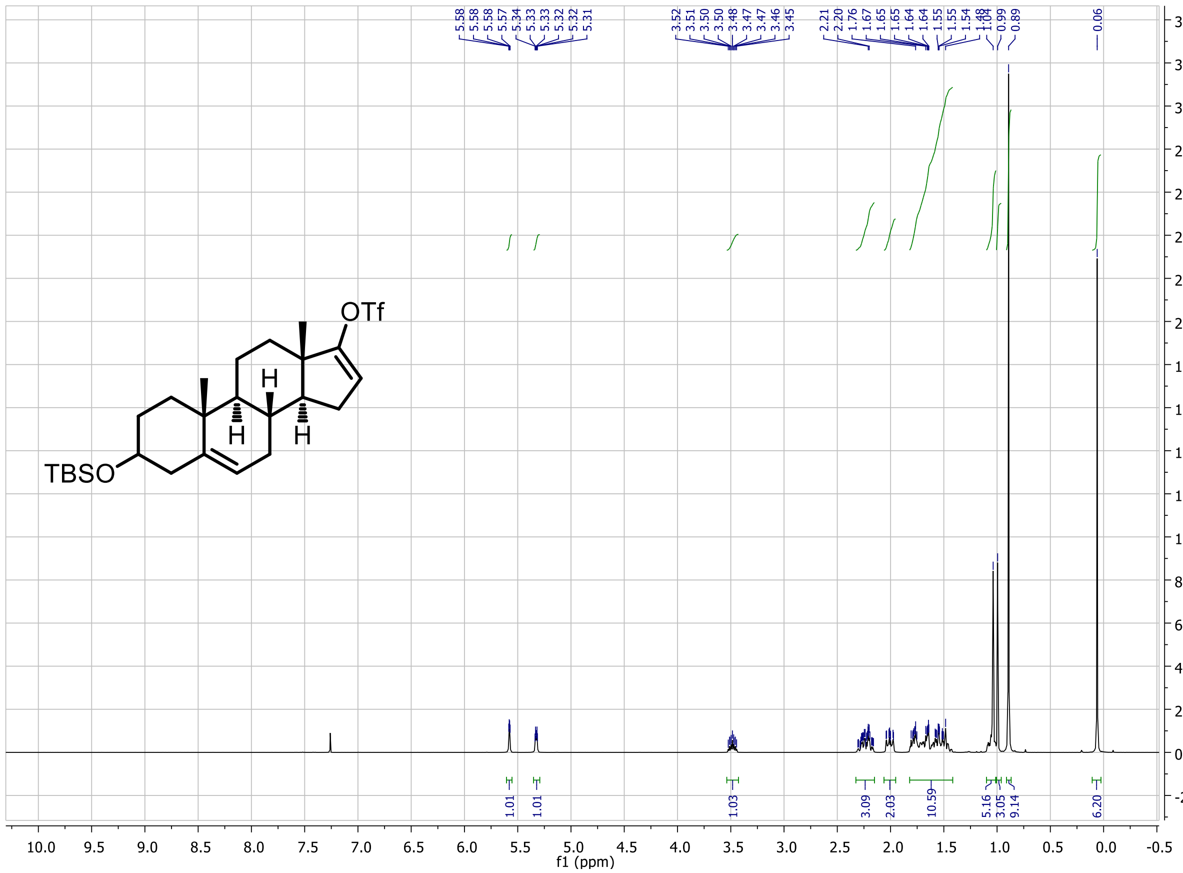
OTf
TBSO
f1 (ppm)

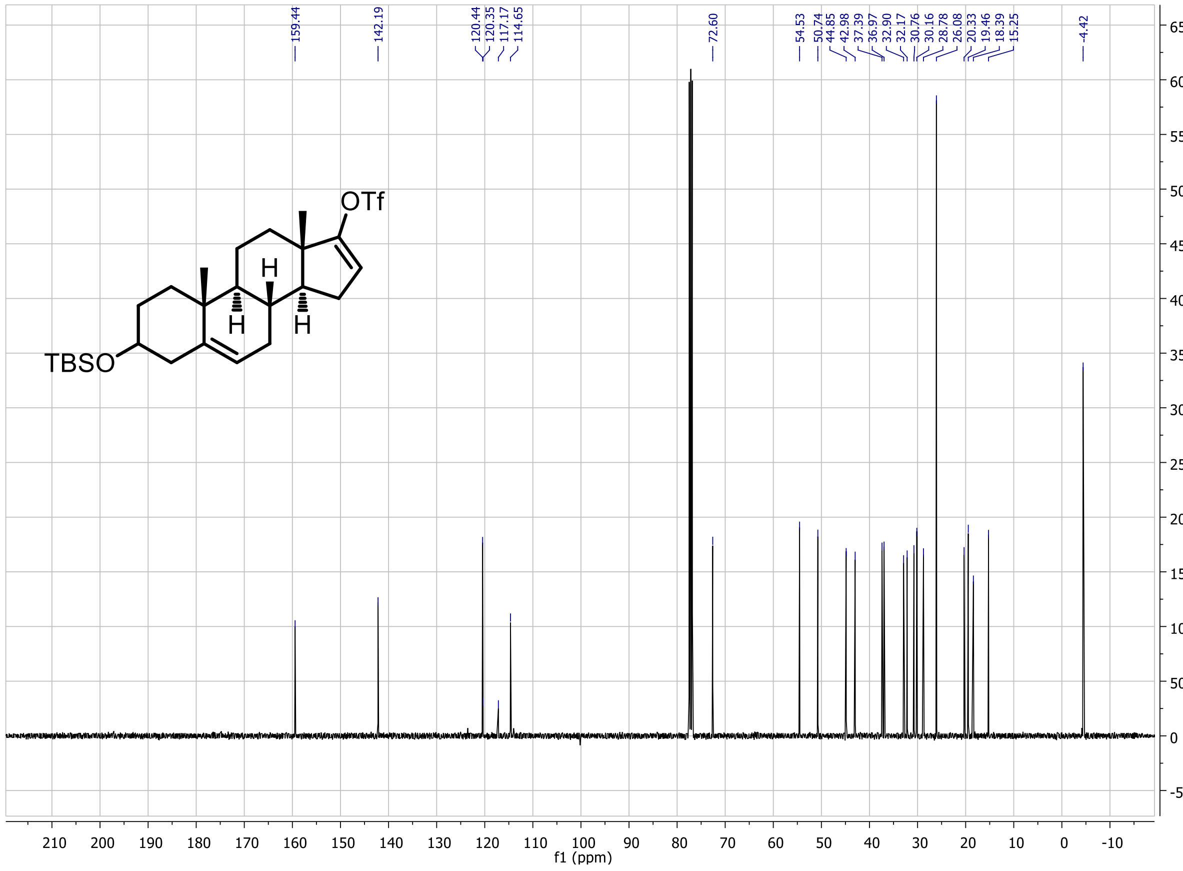
OTf
TBSO
f1 (ppm)

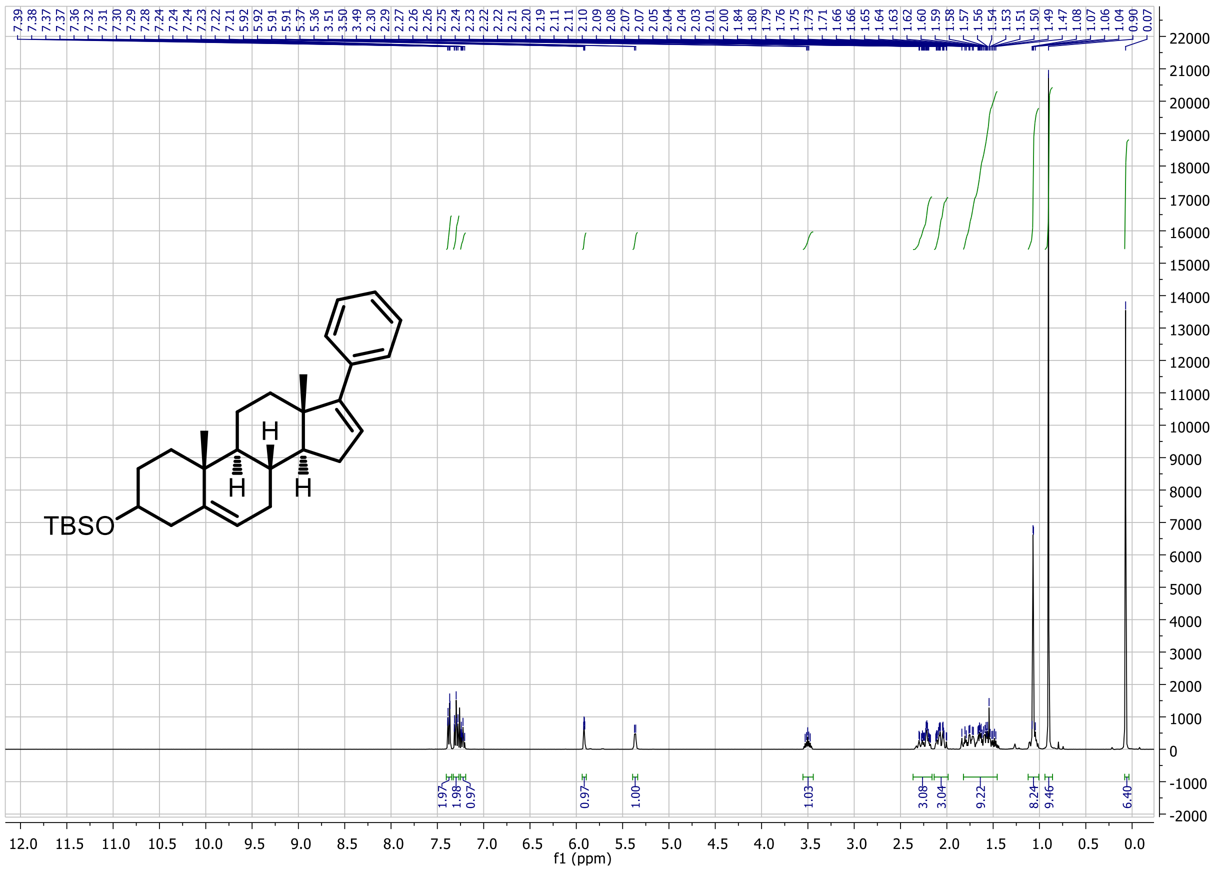
TBSO
H
H
H
f1 (ppm)

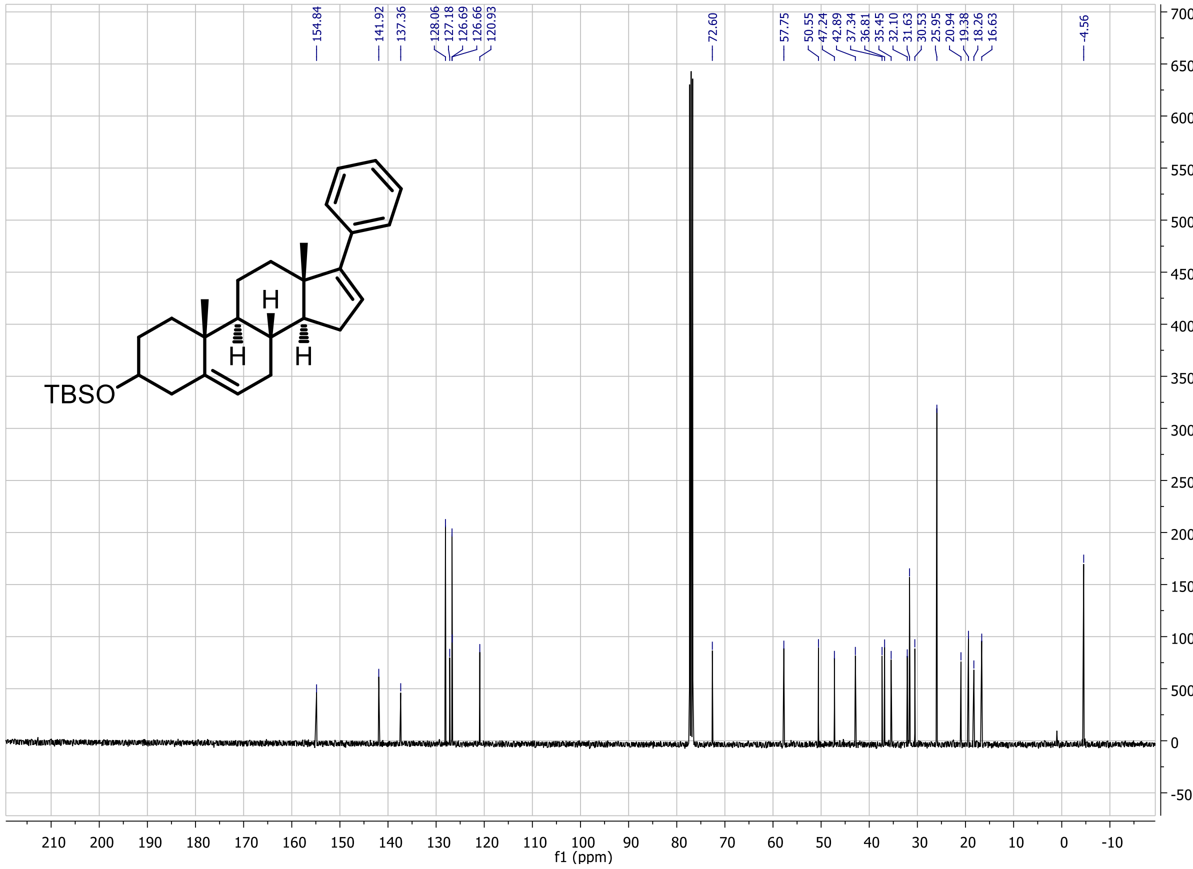
TBSO
H
H
H
f1 (ppm)

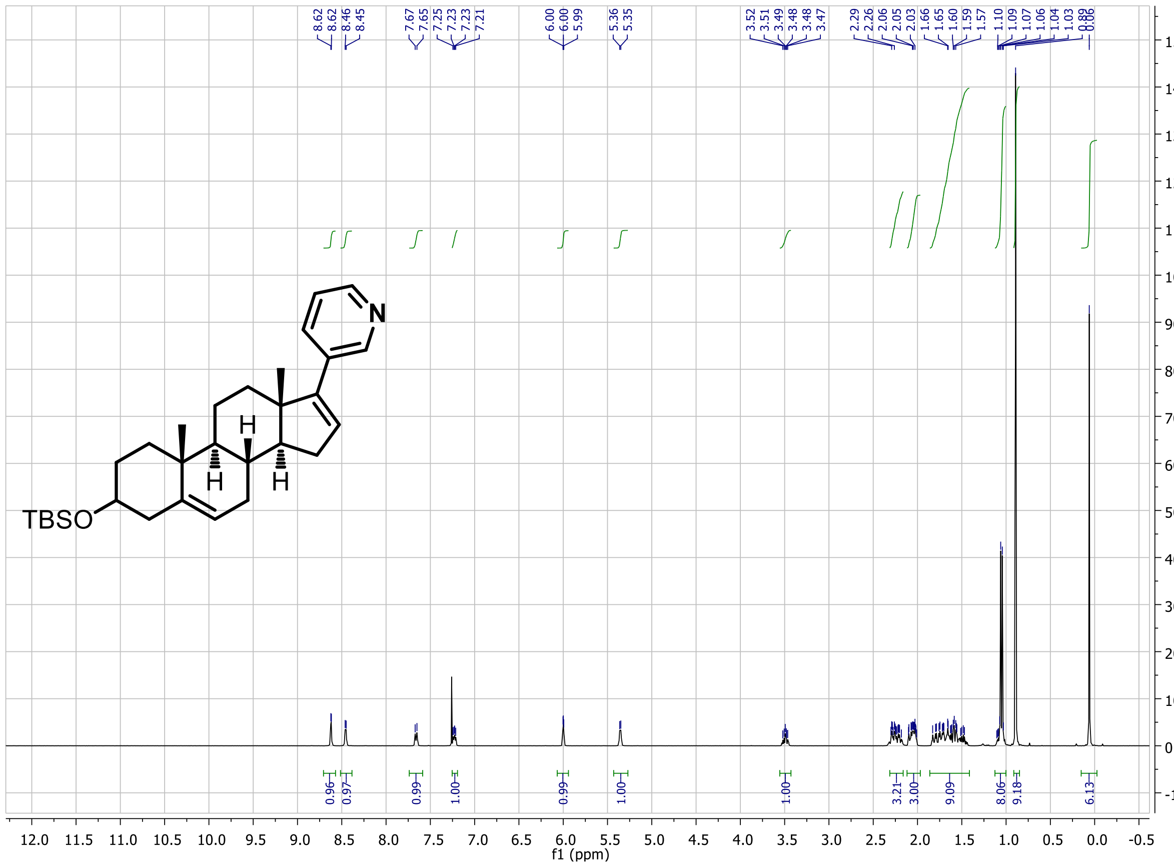
8.62 8.62 8.46 8.45 7.67 7.65 7.25 7.23 7.23 7.21 6.00 6.00 5.99 5.36 5.35 3.52 3.51 3.49 3.48 3.48 3.47 2.29 2.26 2.06 2.05 2.03 1.66 1.65 1.60 1.59 1.57 1.10 1.09 1.07 1.06 1.04 1.03 0.89 0.06
0.96 0.97 0.99 1.00 0.99 1.00 1.00 3.21 3.00 9.09 8.06 9.18 6.13
N
H
H
H
TBSO
f1 (ppm)

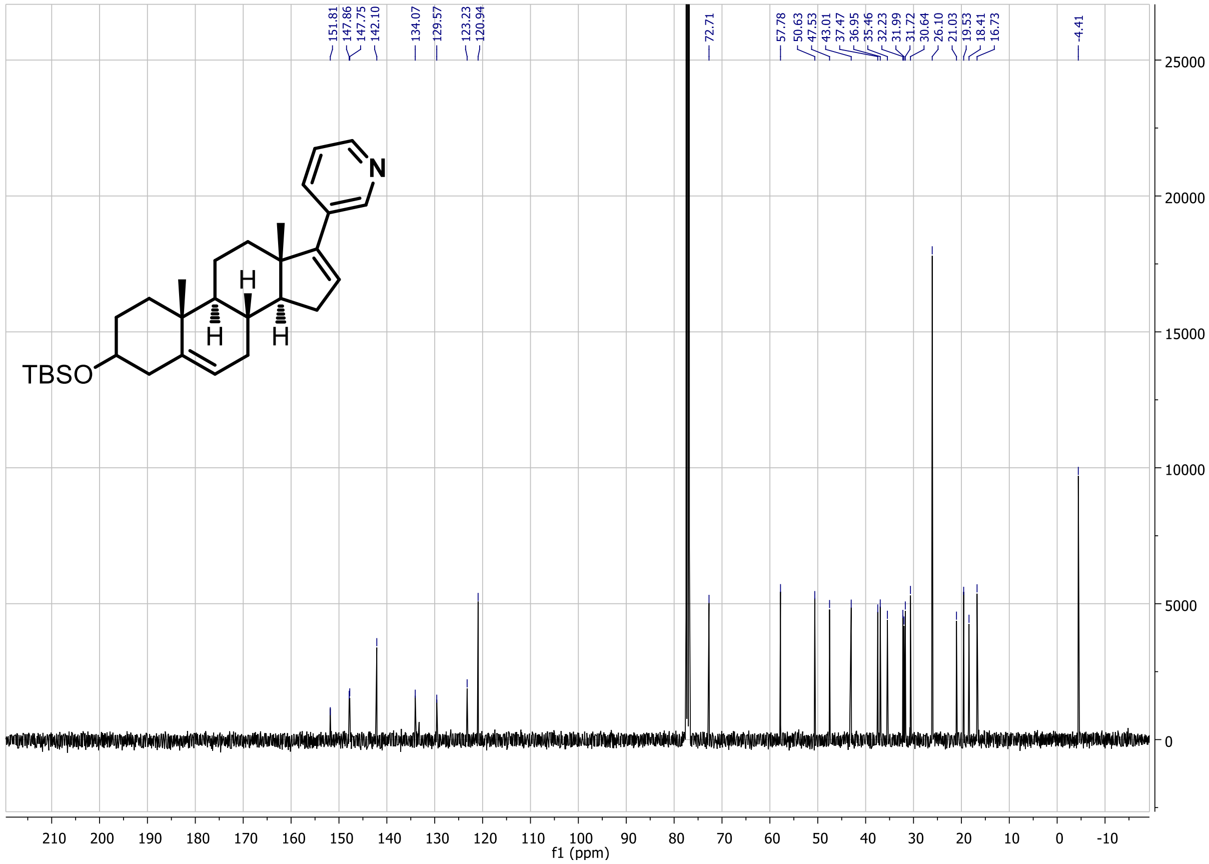
151.81 147.86 147.75 142.10 134.07 129.57 123.23 120.94 72.71 57.78 50.63 47.53 43.01 37.47 36.95 35.46 32.23 31.99 31.72 30.64 26.10 21.03 19.53 18.41 16.73 -4.41
N
H
H
H
TBSO
f1 (ppm)

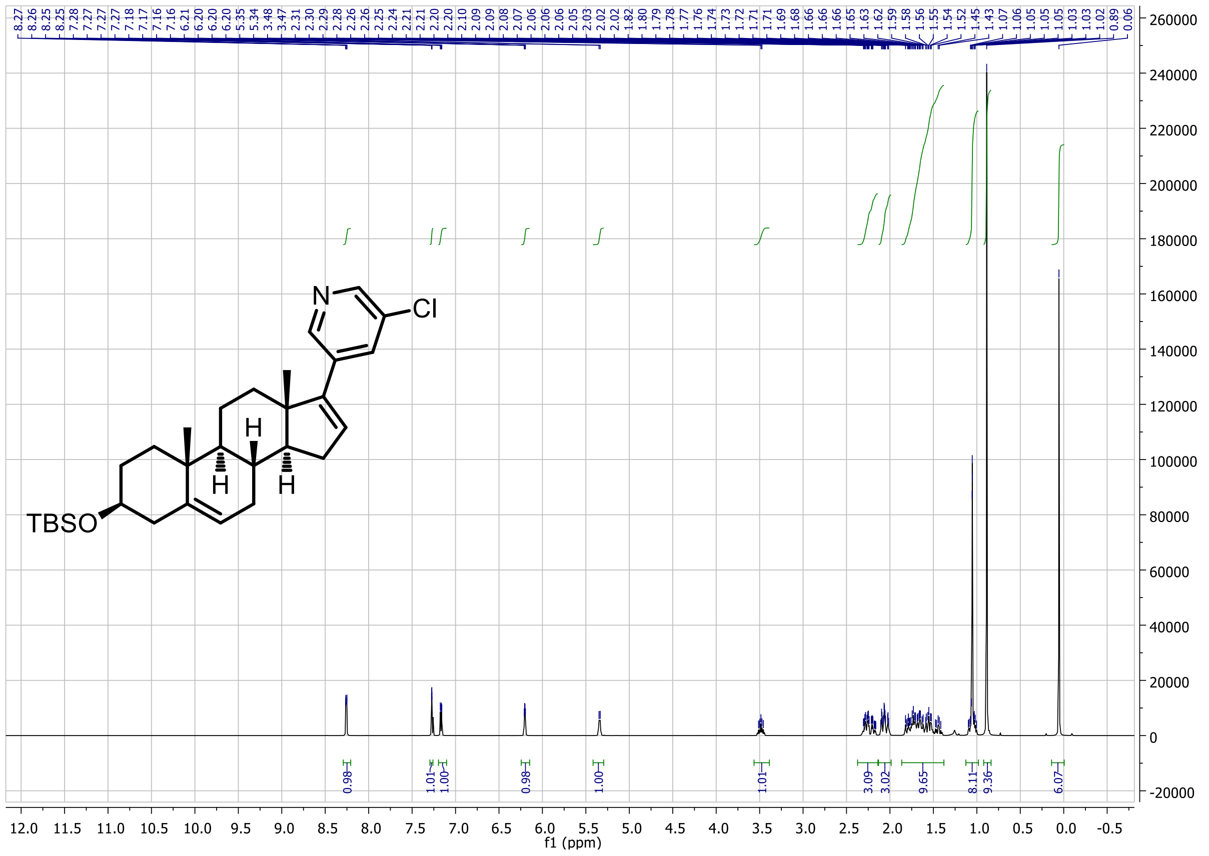

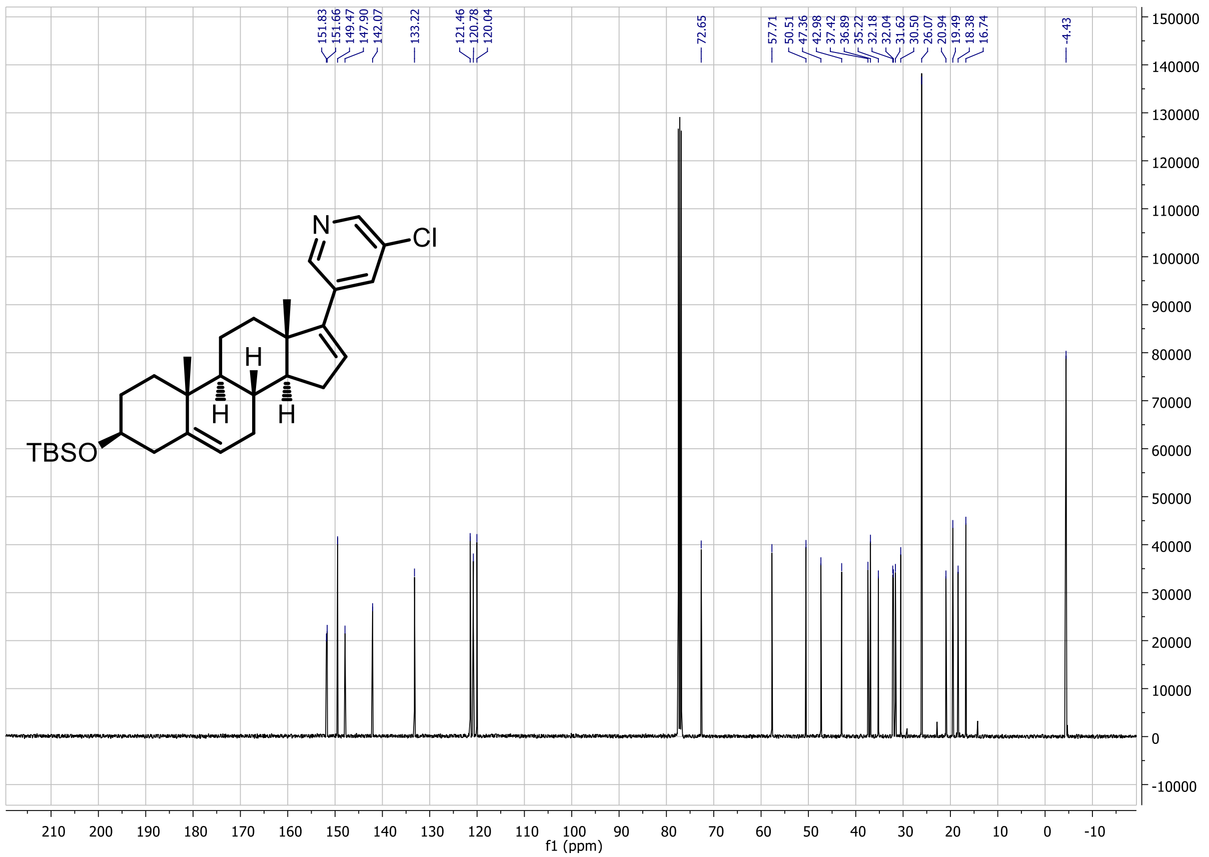

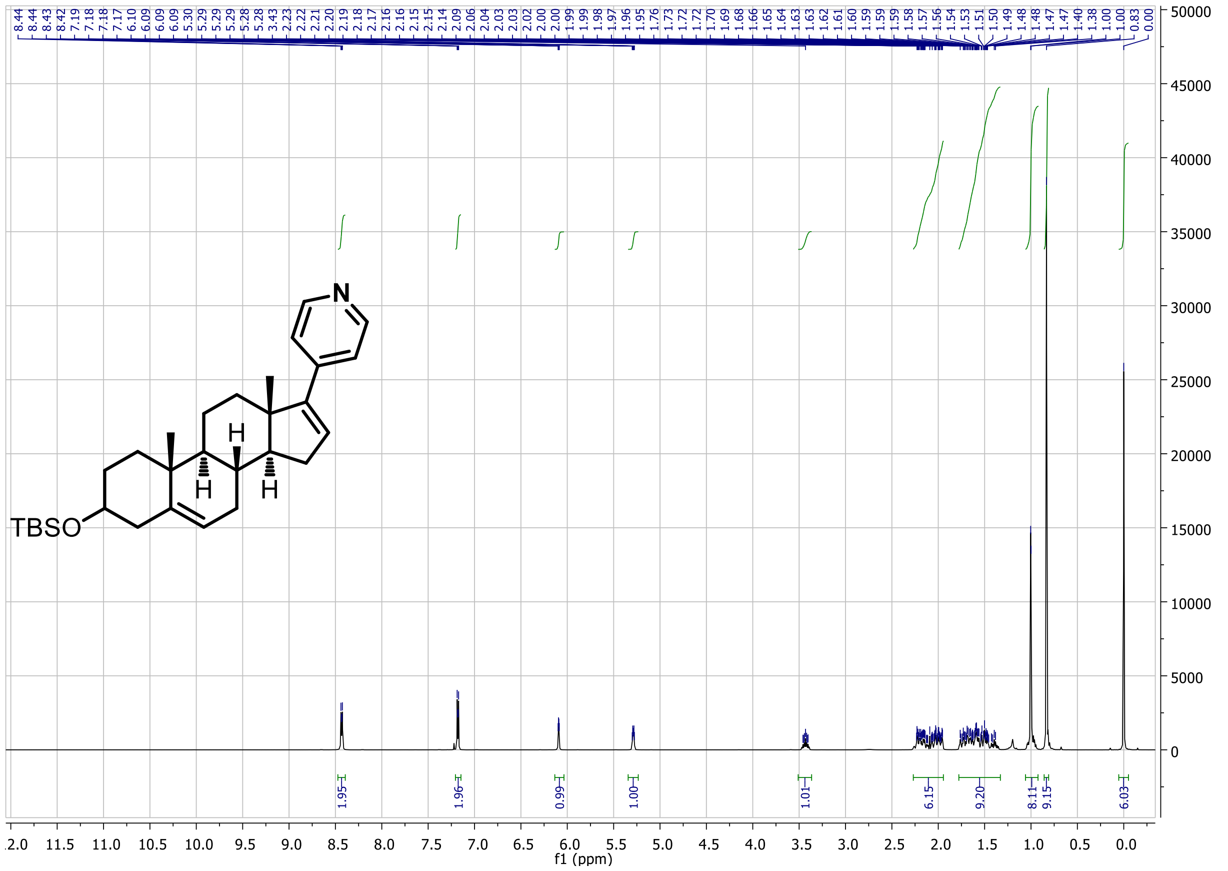

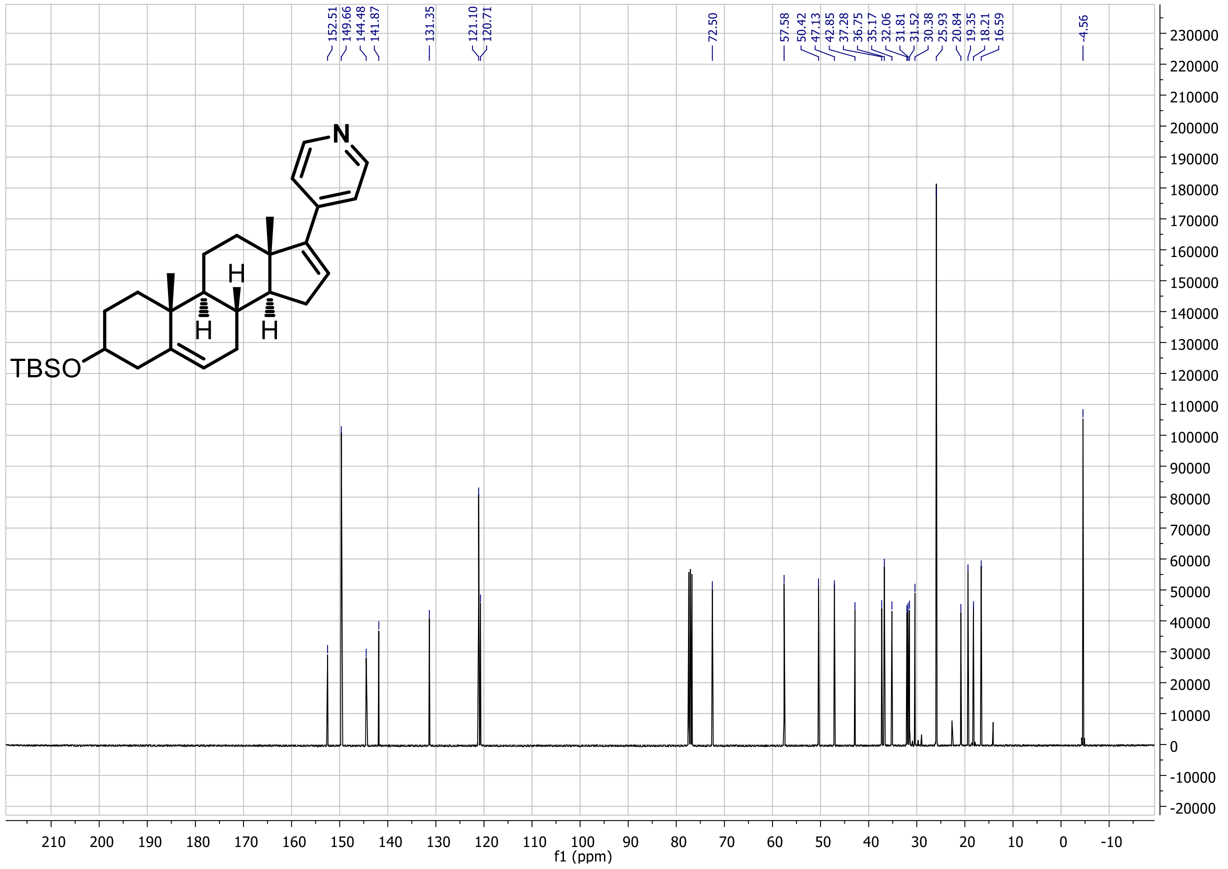

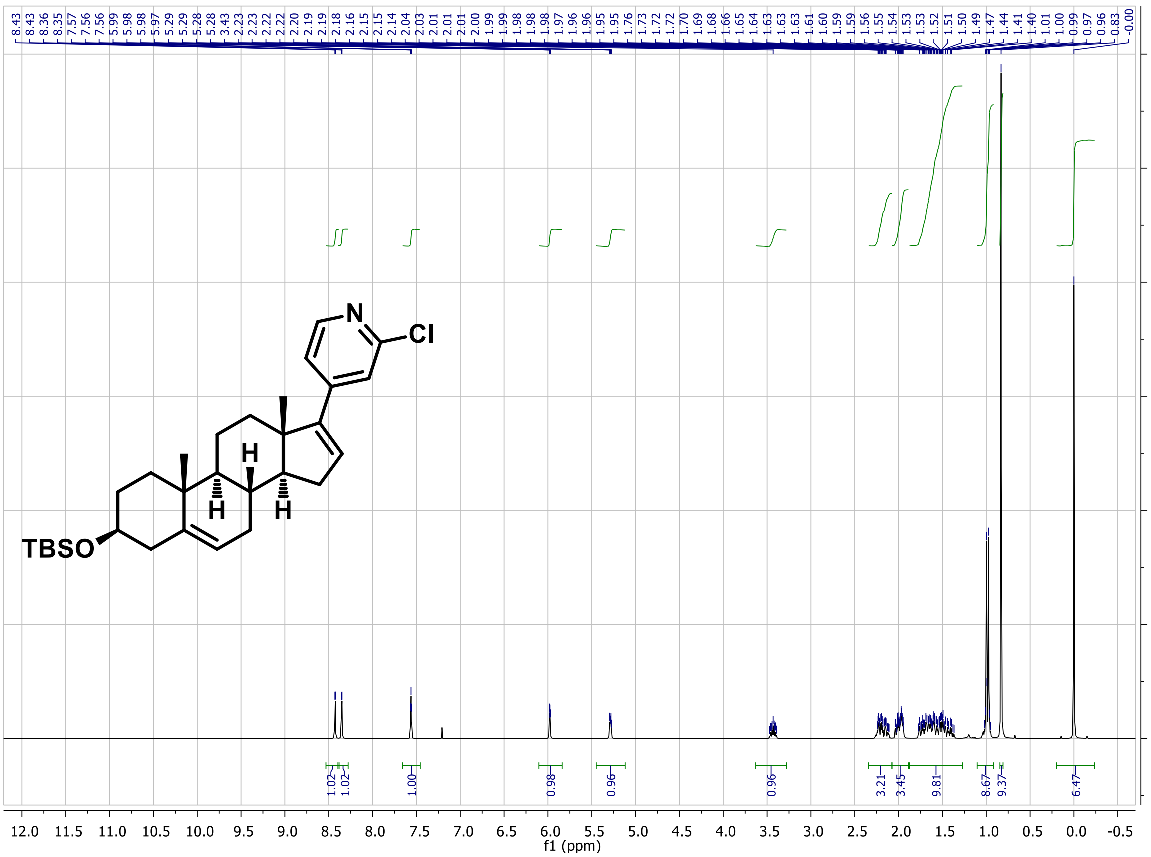
8.43 8.43 8.36 8.35 7.57 7.56 7.56 5.99 5.98 5.98 5.97 5.29 5.29 5.28 5.28 3.43 2.23 2.23 2.22 2.22 2.20 2.19 2.19 2.19 2.18 2.16 2.15 2.15 2.14 2.04 2.03 2.01 2.01 2.01 2.00 1.99 1.99 1.98 1.98 1.98 1.97 1.96 1.96 1.95 1.95 1.76 1.73 1.72 1.72 1.70 1.69 1.68 1.66 1.65 1.64 1.63 1.63 1.63 1.61 1.60 1.59 1.59 1.56 1.55 1.54 1.53 1.53 1.52 1.51 1.50 1.49 1.47 1.44 1.41 1.40 1.01 1.00 0.99 0.97 0.96 0.83 -0.00
1.02 1.02 1.00 0.98 0.96 0.96 3.21 3.45 9.81 8.67 9.37 6.47
N
Cl
H
H
H
TBSO
f1 (ppm)

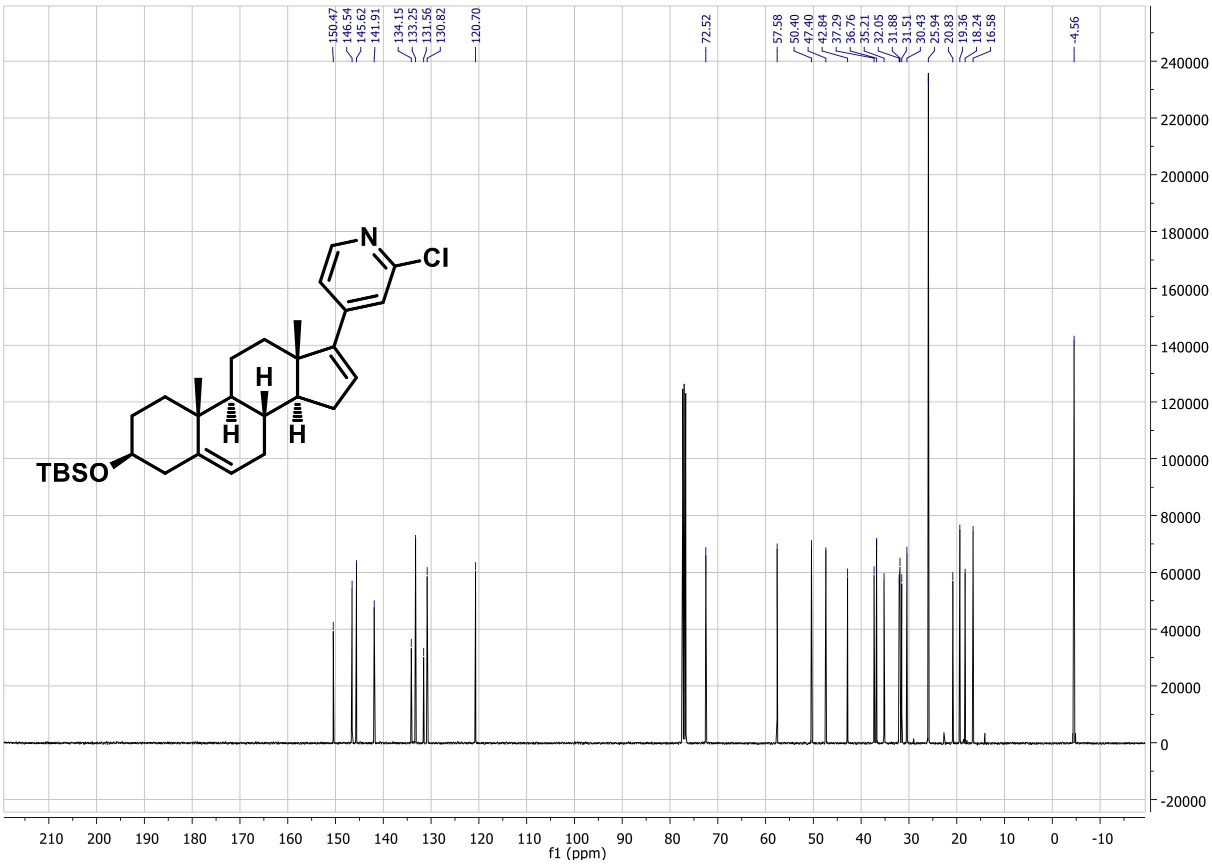
150.47 146.54 145.62 141.91 134.15 133.25 131.56 130.82 120.70 72.52 57.58 50.40 47.40 42.84 37.29 36.76 35.21 32.05 31.88 31.51 30.43 25.94 20.83 19.36 18.24 16.58 -4.56
N
Cl
H
H
H
TBSO
f1 (ppm)

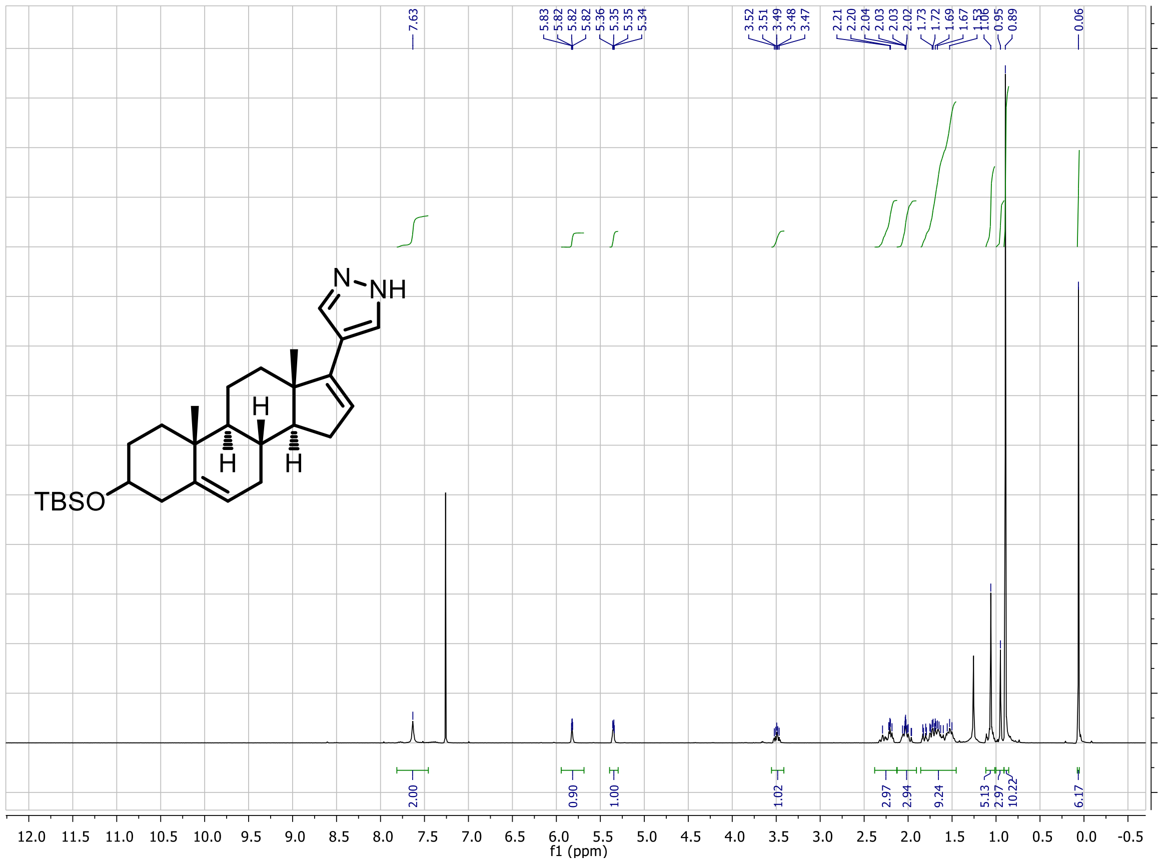

TBSO
N
NH
H
H
H
7.63
5.83
5.82
5.82
5.82
5.36
5.35
5.35
5.34
3.52
3.51
3.49
3.48
3.47
2.21
2.20
2.04
2.03
2.03
2.02
1.73
1.72
1.69
1.67
1.53
1.06
0.95
0.89
0.06
2.00
0.90
1.00
1.02
2.97
2.94
9.24
5.13
2.97
10.22
6.17
f1 (ppm)

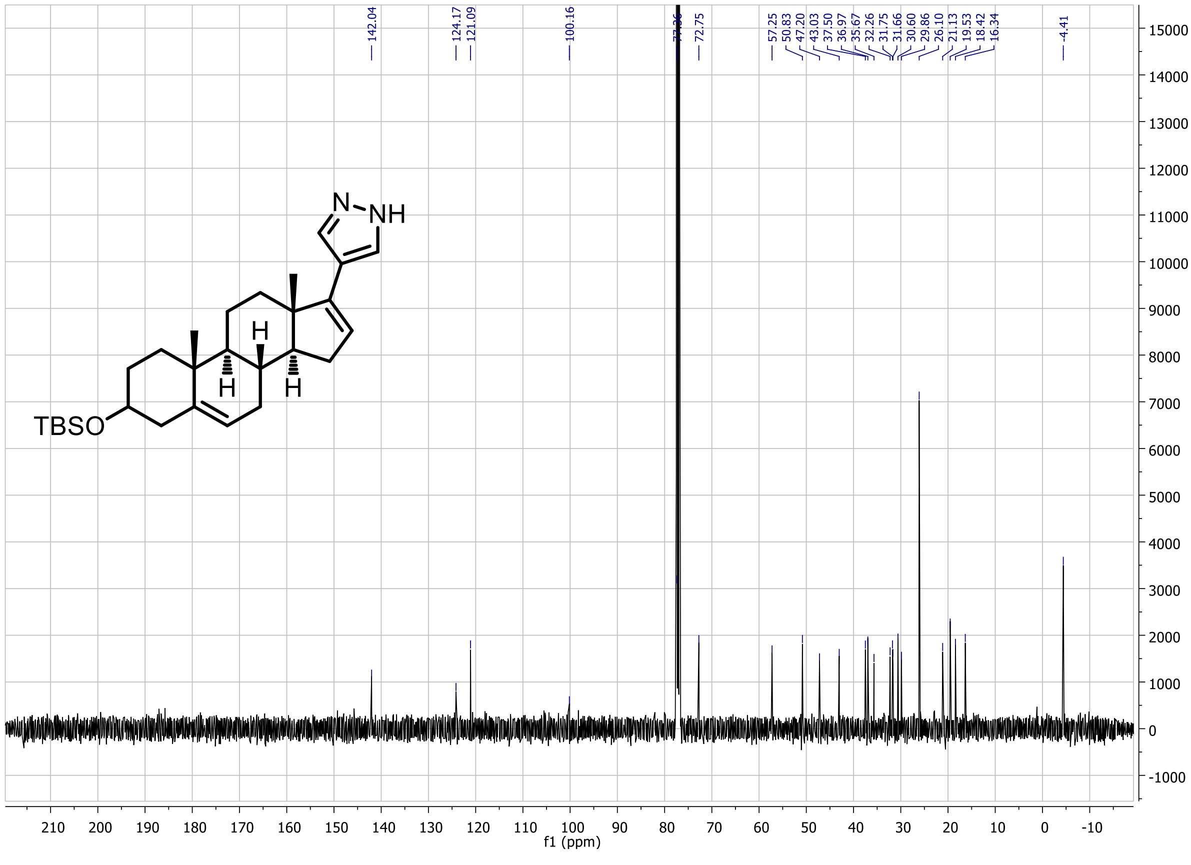

TBSO
N
NH
H
H
H
142.04
124.17
121.09
100.16
72.75
57.25
50.83
47.20
43.03
37.50
36.97
35.67
32.26
31.75
31.66
30.60
29.86
26.10
21.13
19.53
18.42
16.34
-4.41
f1 (ppm)

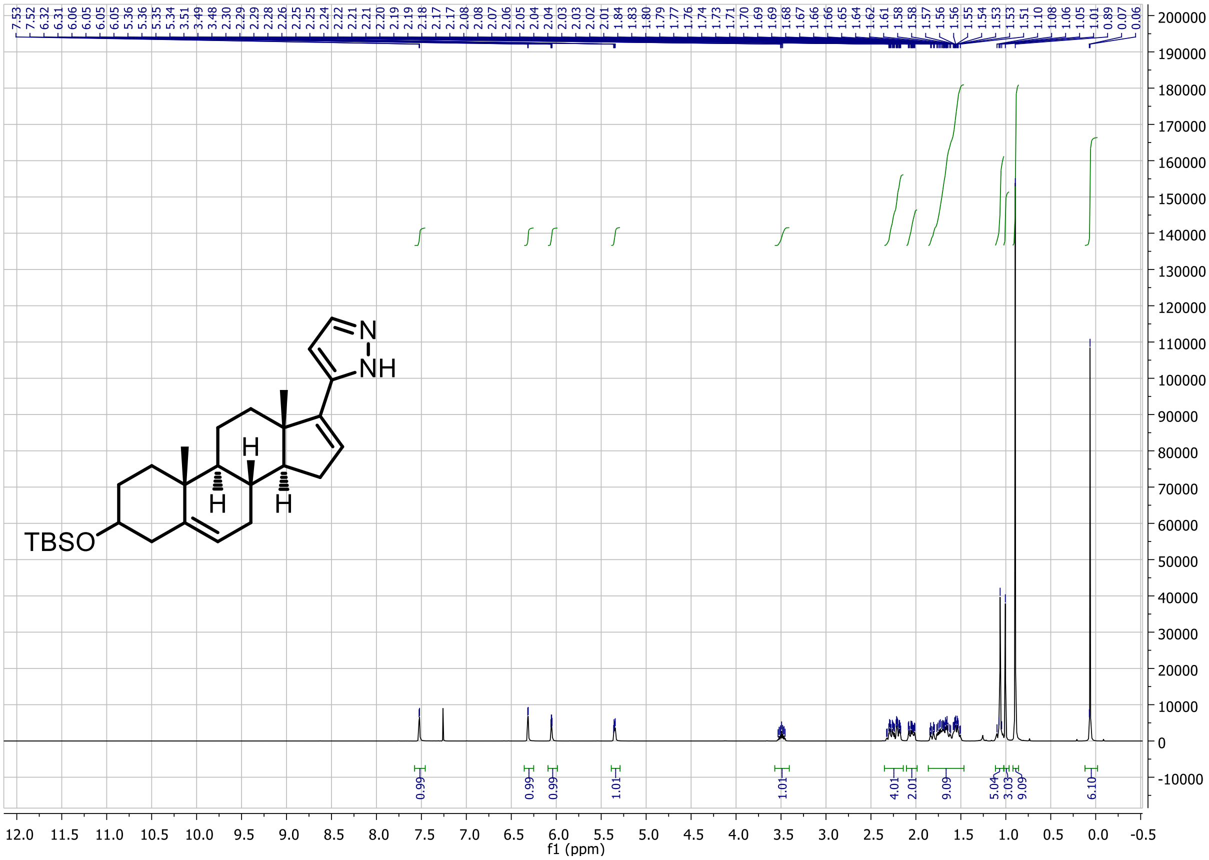
7.53
7.52
6.32
6.31
6.06
6.05
6.05
6.05
5.36
5.36
5.35
5.34
3.51
3.49
3.48
2.30
2.29
2.29
2.28
2.26
2.25
2.25
2.24
2.22
2.21
2.21
2.20
2.19
2.19
2.18
2.17
2.17
2.08
2.08
2.07
2.06
2.05
2.04
2.04
2.03
2.03
2.02
2.01
1.84
1.83
1.80
1.79
1.77
1.76
1.74
1.73
1.71
1.70
1.69
1.69
1.68
1.67
1.66
1.65
1.64
1.64
1.62
1.61
1.58
1.58
1.57
1.56
1.56
1.55
1.54
1.53
1.53
1.51
1.10
1.08
1.06
1.05
1.01
0.89
0.07
0.06
N
NH
H
H
H
TBSO
0.99
0.99
0.99
1.01
1.01
4.01
2.01
9.09
5.04
3.03
9.09
6.10
f1 (ppm)

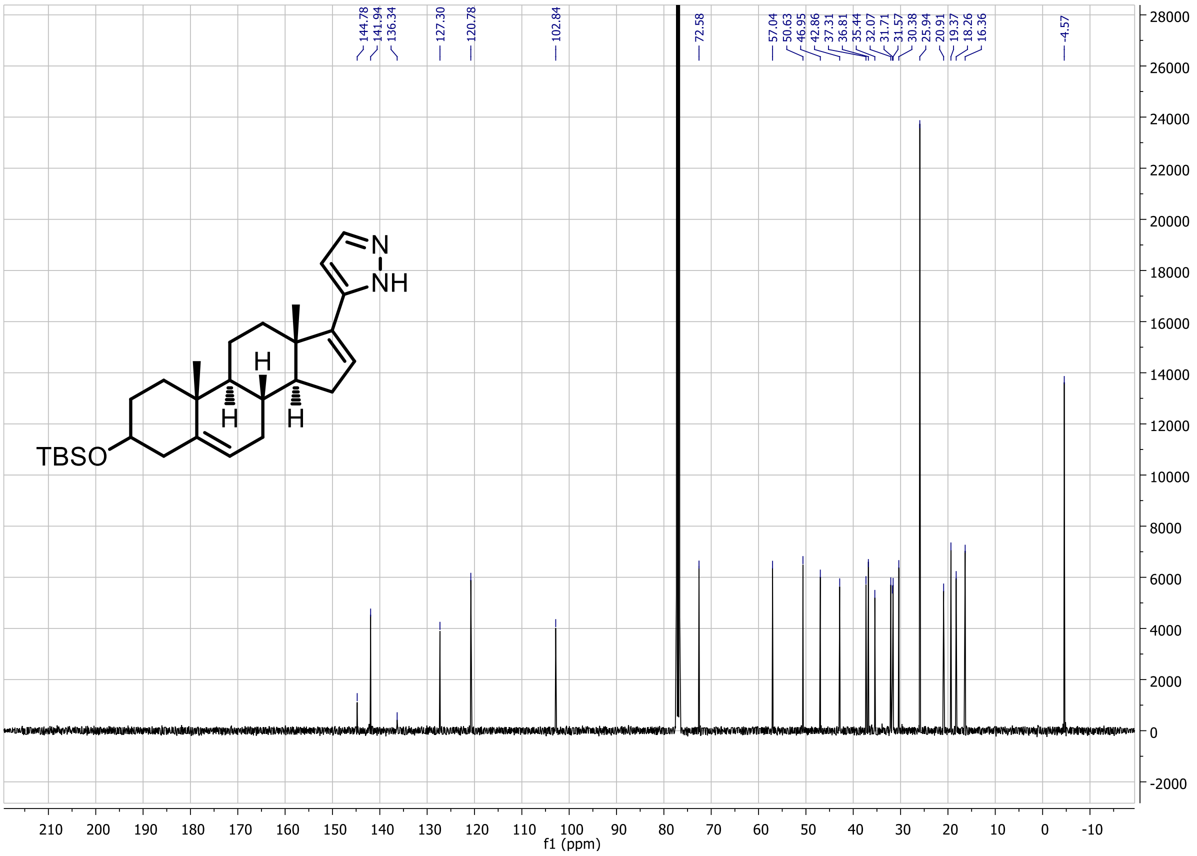
144.78
141.94
136.34
127.30
120.78
102.84
72.58
57.04
50.63
46.95
42.86
37.31
36.81
35.44
32.07
31.71
31.57
30.38
25.94
20.91
19.37
18.26
16.36
-4.57
N
NH
H
H
H
TBSO
f1 (ppm)

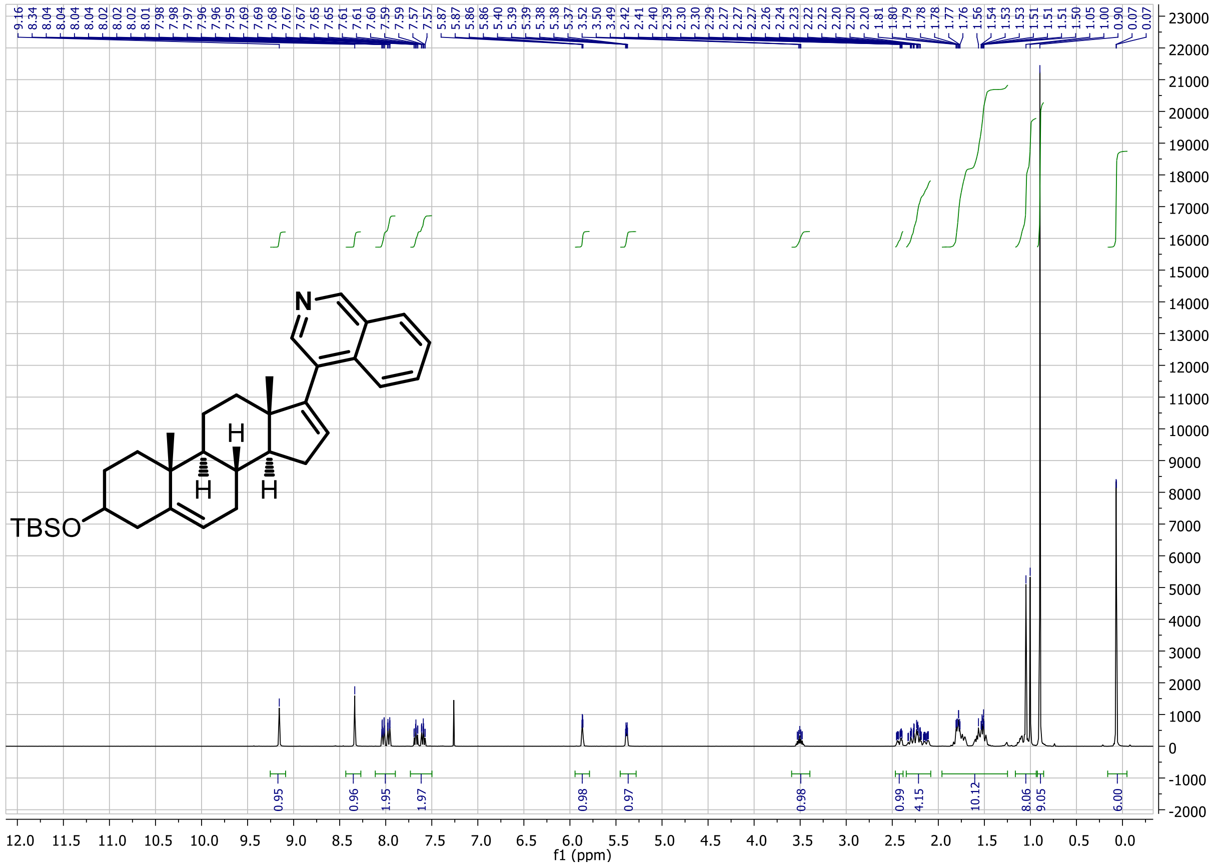
9.16 8.34 8.04 8.04 8.04 8.04 8.02 8.02 8.02 8.01 7.98 7.98 7.97 7.96 7.96 7.95 7.69 7.69 7.68 7.67 7.67 7.65 7.61 7.61 7.60 7.59 7.59 7.57 7.57 5.87 5.87 5.86 5.40 5.39 5.39 5.38 5.37 3.52 3.50 3.49 2.42 2.41 2.40 2.39 2.30 2.30 2.29 2.27 2.27 2.27 2.26 2.24 2.23 2.22 2.22 2.22 2.20 2.20 2.20 2.20 1.81 1.80 1.79 1.78 1.78 1.77 1.76 1.56 1.54 1.53 1.53 1.51 1.51 1.51 1.50 1.05 1.00 0.90 0.07 0.07
N
H
H
H
TBSO
0.95 0.96 1.95 1.97 0.98 0.97 0.98 0.99 4.15 10.12 8.06 9.05 6.00
f1 (ppm)

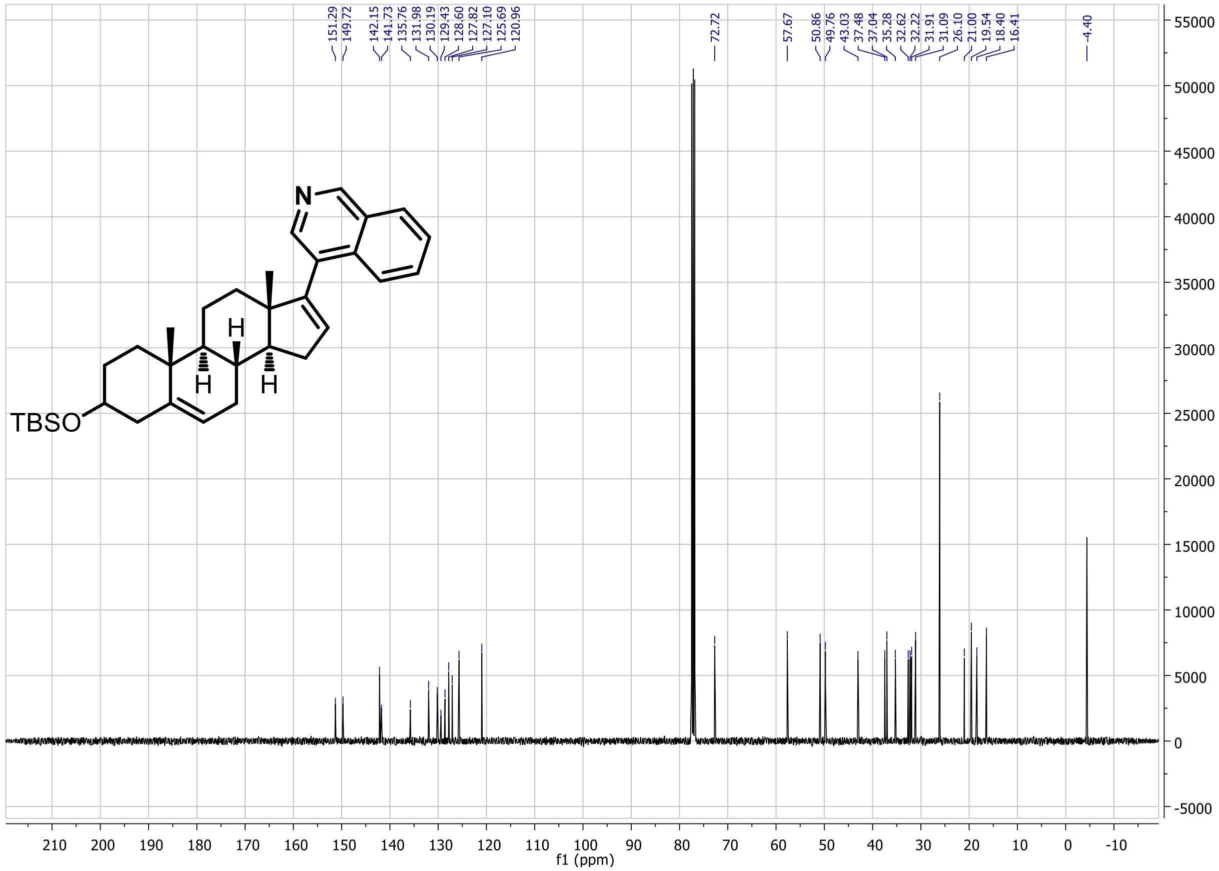
151.29 149.72 142.15 141.73 135.76 131.98 130.19 129.43 128.60 127.82 127.10 125.69 120.96
72.72
57.67 50.86 49.76 43.03 37.48 37.04 35.28 32.62 32.22 31.91 31.09 26.10 21.00 19.54 18.40 16.41
-4.40
N
H
H
H
TBSO
f1 (ppm)

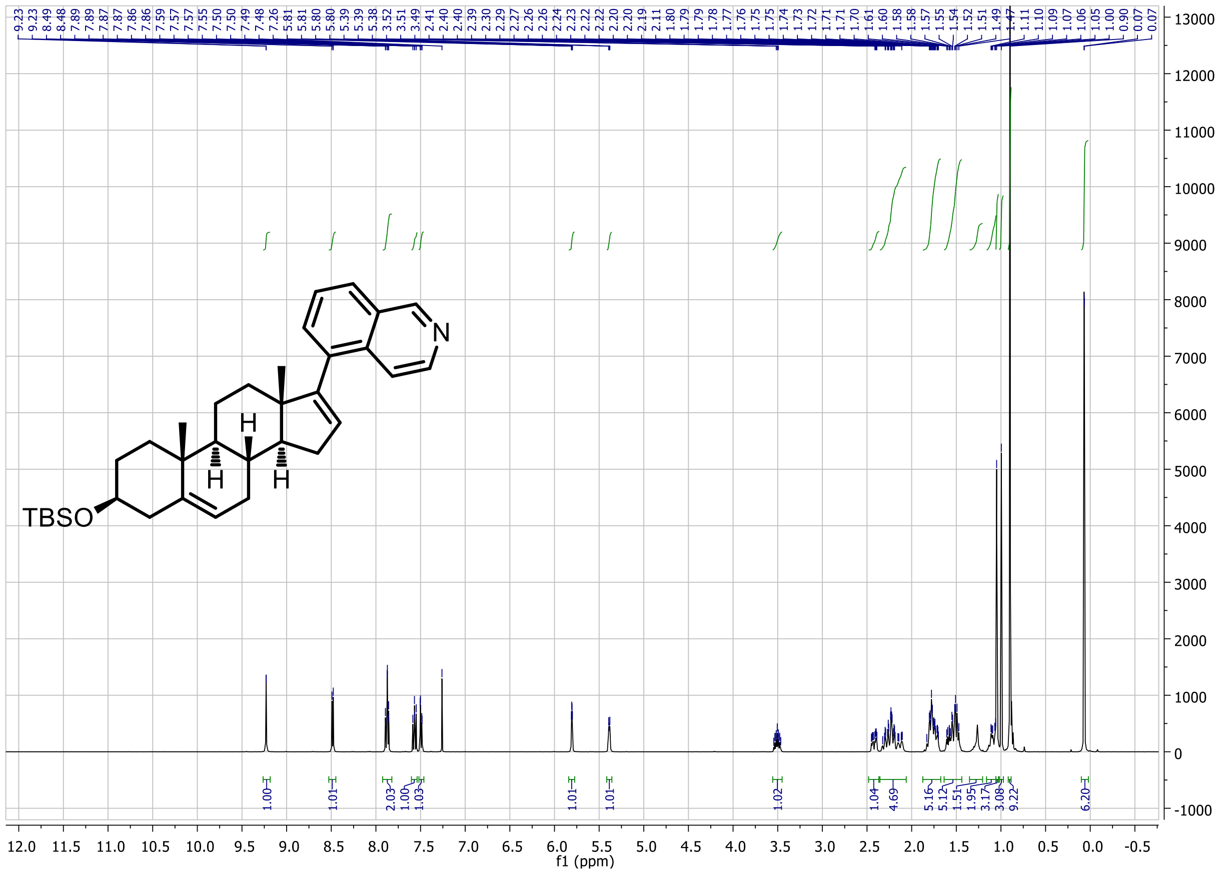
f1 (ppm)
TBSO
N
H
H
H

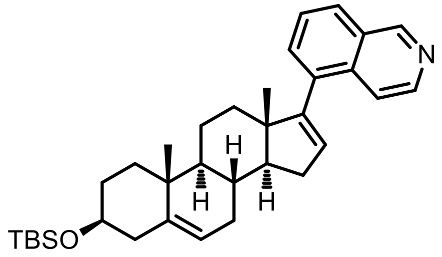
TBSO
N
H
H
H

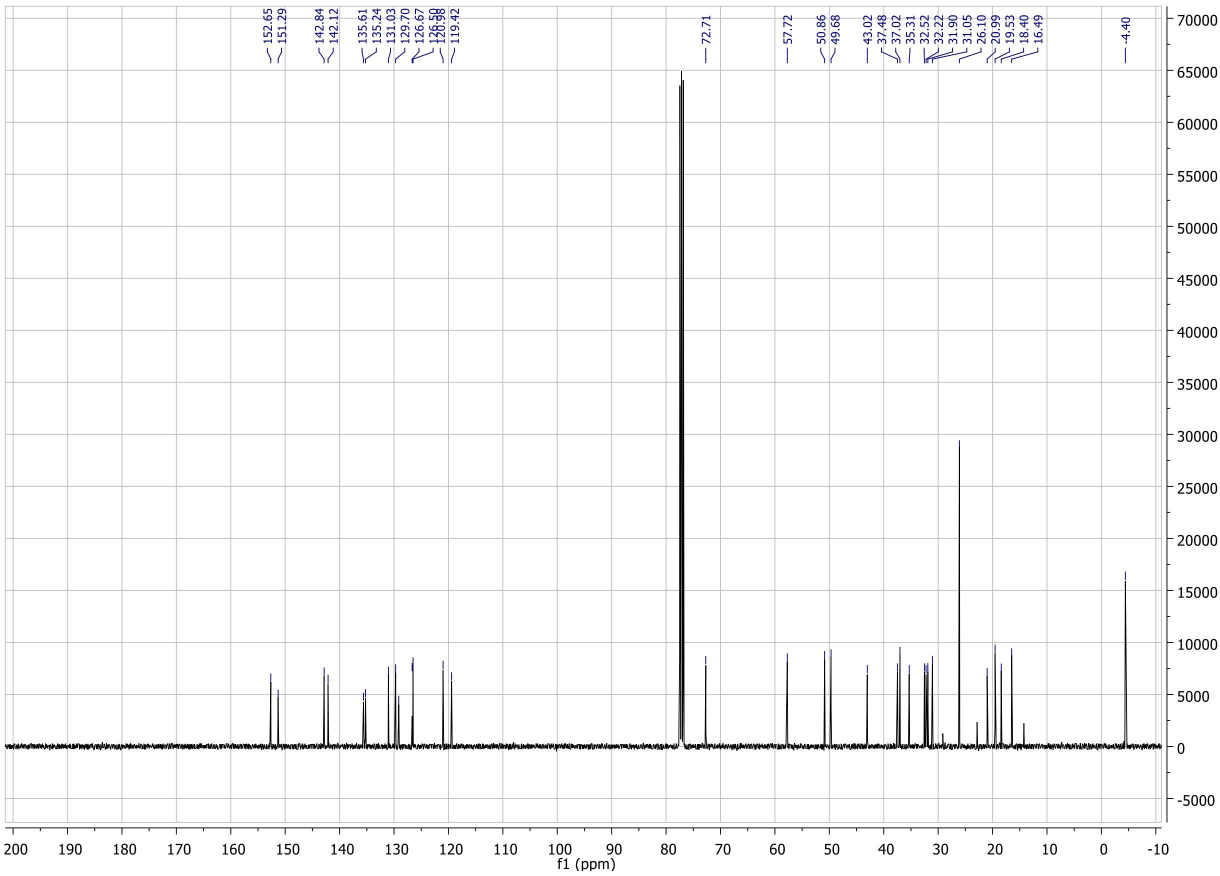
152.65
151.29
142.84
142.12
135.61
135.24
131.03
129.70
126.67
126.50
120.98
119.42
72.71
57.72
50.86
49.68
43.02
37.48
37.02
35.31
32.52
32.22
31.90
31.05
26.10
20.99
19.53
18.40
16.49
-4.40
f1 (ppm)

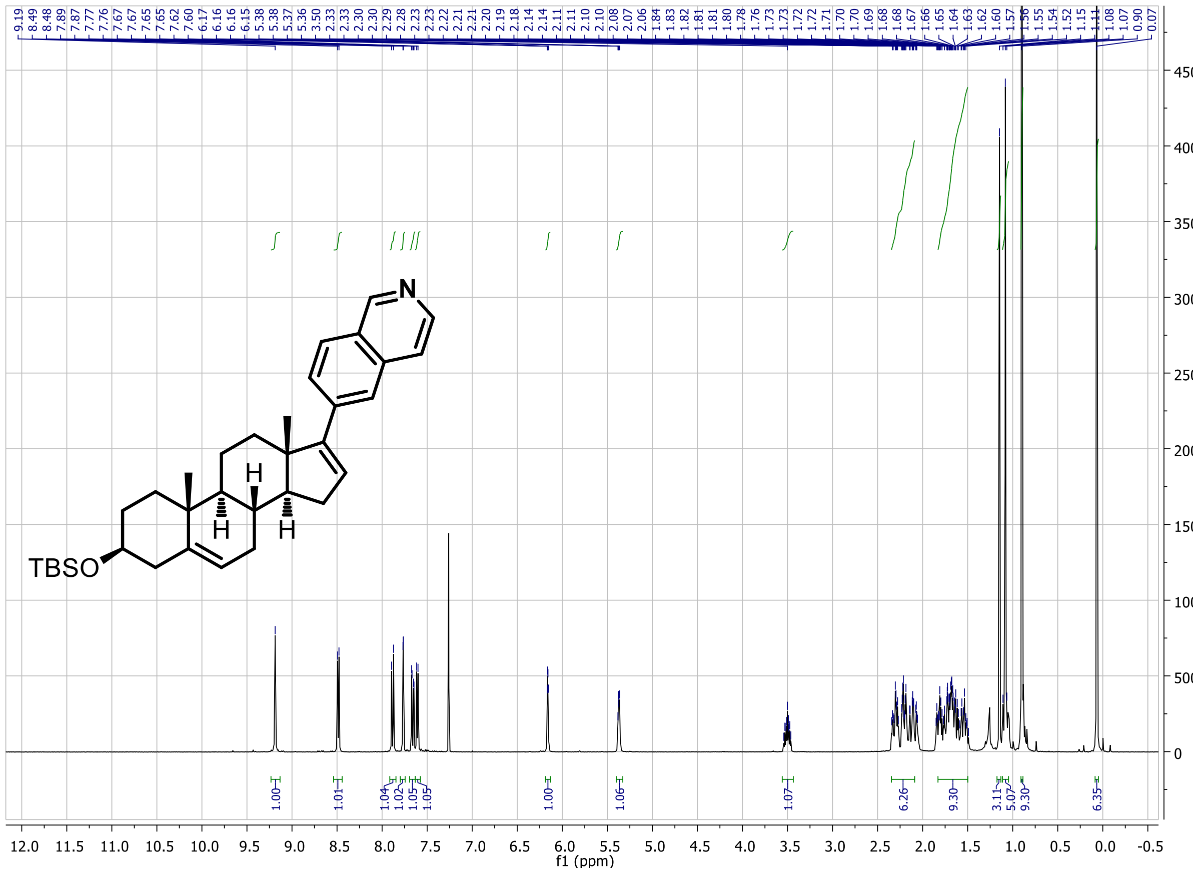

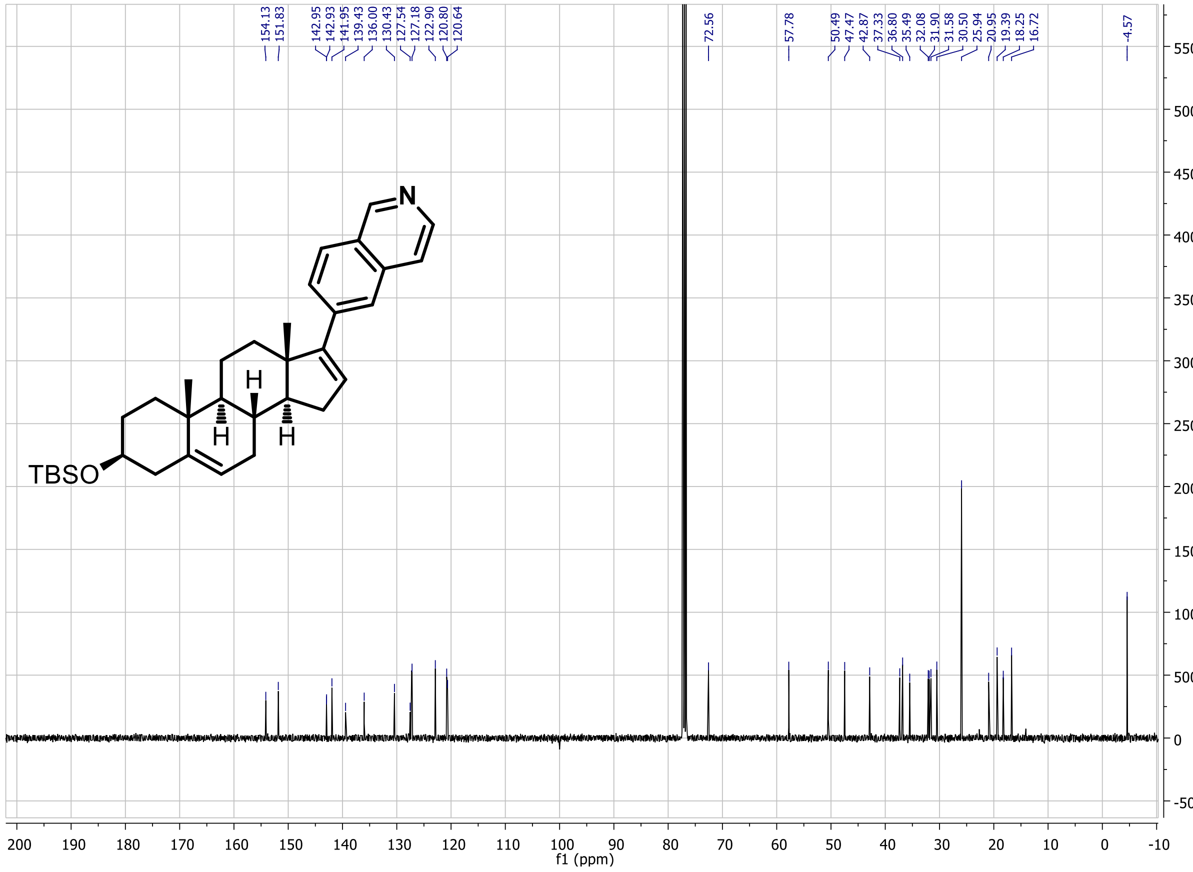

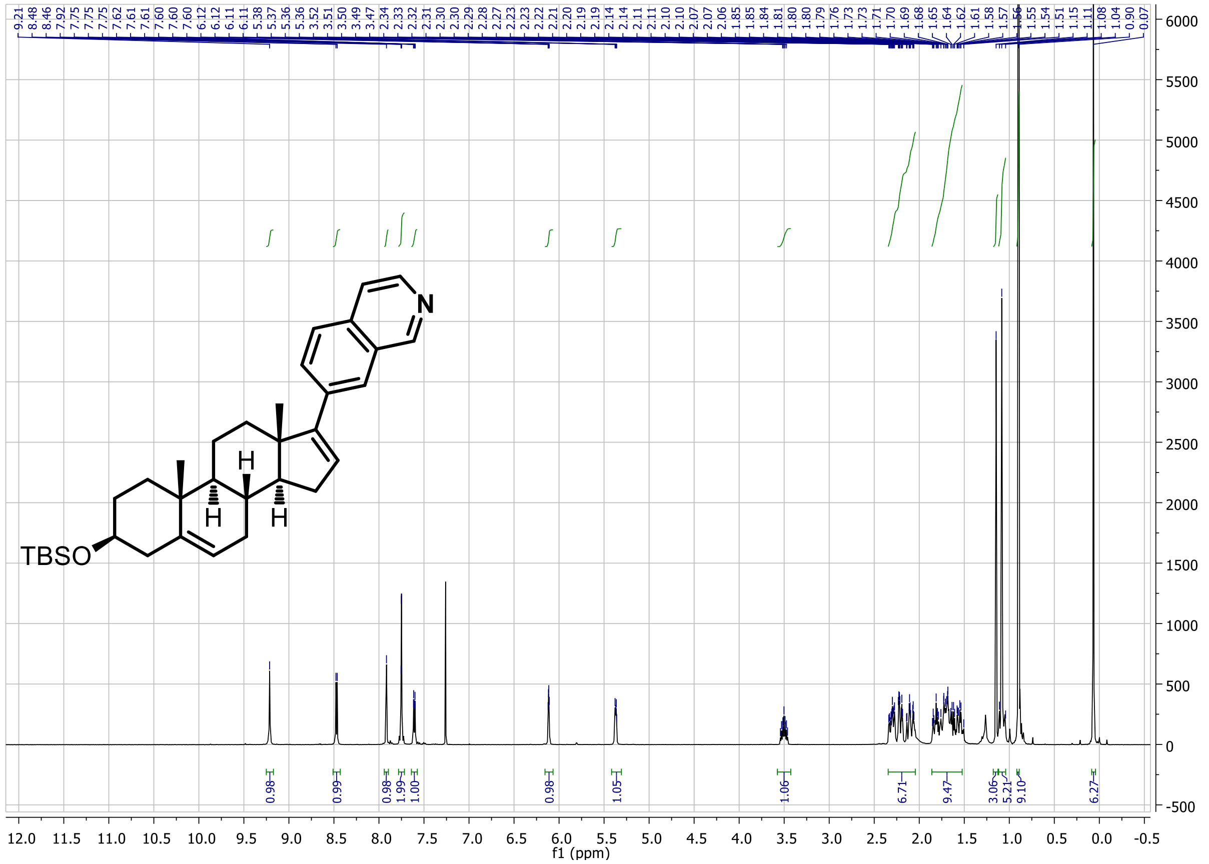
9.21 8.48 8.46 7.92 7.75 7.75 7.75 7.62 7.61 7.61 7.60 7.60 7.60 6.12 6.12 6.11 6.11 5.38 5.37 5.36 5.36 3.52 3.51 3.50 3.49 3.47 2.34 2.33 2.32 2.31 2.30 2.30 2.29 2.28 2.27 2.23 2.23 2.22 2.21 2.20 2.19 2.19 2.14 2.14 2.11 2.11 2.10 2.10 2.07 2.07 2.06 1.85 1.85 1.84 1.81 1.80 1.80 1.79 1.76 1.73 1.73 1.71 1.70 1.69 1.68 1.65 1.64 1.62 1.61 1.58 1.57 1.56 1.55 1.54 1.51 1.15 1.11 1.08 1.04 0.90 0.07
0.98 0.99 0.98 1.99 1.00 0.98 1.05 1.06 6.71 9.47 3.06 5.21 9.10 6.27
TBSO
H
H
H
N
f1 (ppm)

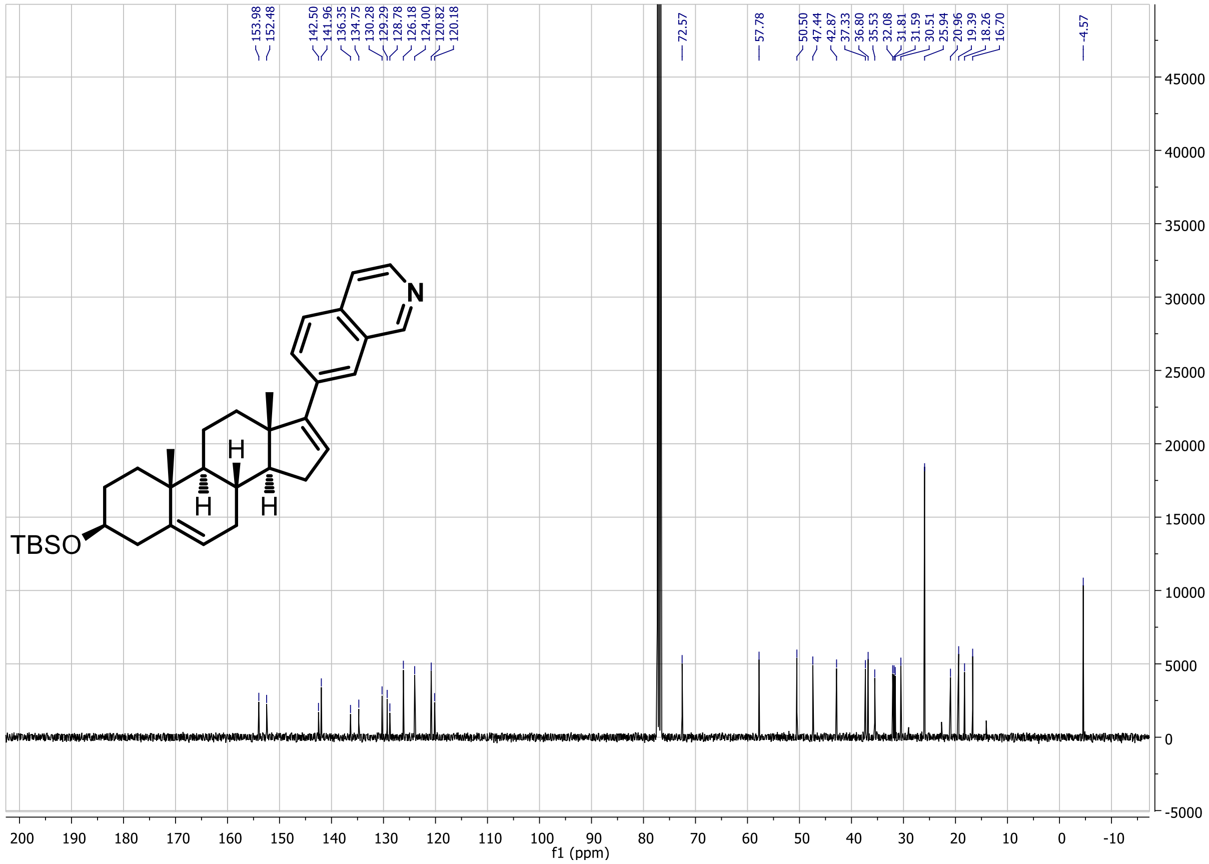
153.98 152.48 142.50 141.96 136.35 134.75 130.28 129.29 128.78 126.18 124.00 120.82 120.18 72.57 57.78 50.50 47.44 42.87 37.33 36.80 35.53 32.08 31.81 31.59 30.51 25.94 20.96 19.39 18.26 16.70 -4.57
TBSO
H
H
H
N
f1 (ppm)

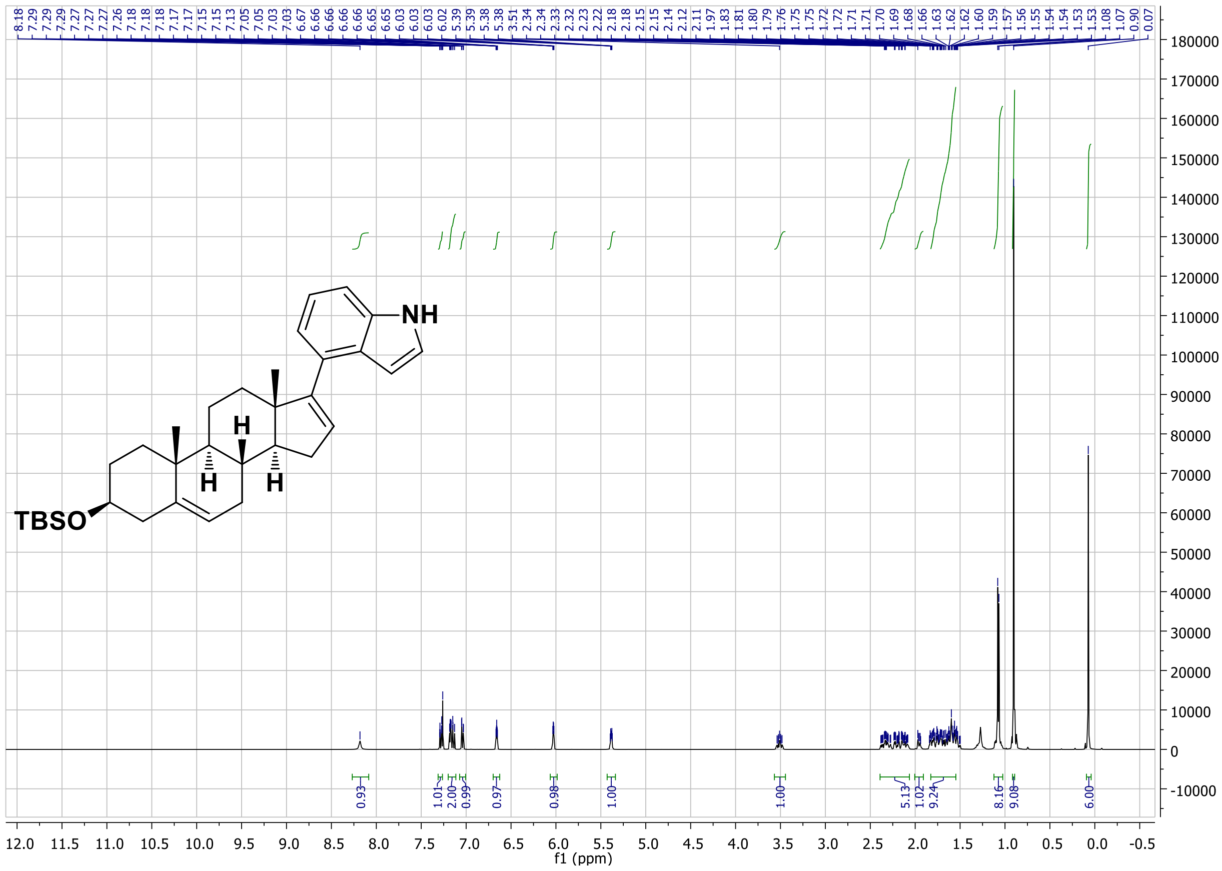
8.18
7.29
7.29
7.29
7.27
7.27
7.27
7.26
7.18
7.18
7.18
7.17
7.17
7.15
7.15
7.13
7.05
7.05
7.03
7.03
6.67
6.66
6.66
6.66
6.66
6.65
6.65
6.03
6.03
6.03
6.02
5.39
5.39
5.39
5.38
5.38
3.51
2.34
2.34
2.33
2.32
2.23
2.22
2.18
2.18
2.15
2.15
2.14
2.12
2.11
1.97
1.83
1.81
1.80
1.79
1.76
1.75
1.75
1.72
1.72
1.71
1.71
1.70
1.69
1.68
1.66
1.63
1.62
1.62
1.60
1.59
1.57
1.56
1.55
1.54
1.54
1.53
1.53
1.08
1.07
0.90
0.07
NH
H
H
H
TBSO
0.93
1.01
2.00
0.99
0.97
0.98
1.00
1.00
5.13
1.02
9.24
8.16
9.08
6.00
f1 (ppm)

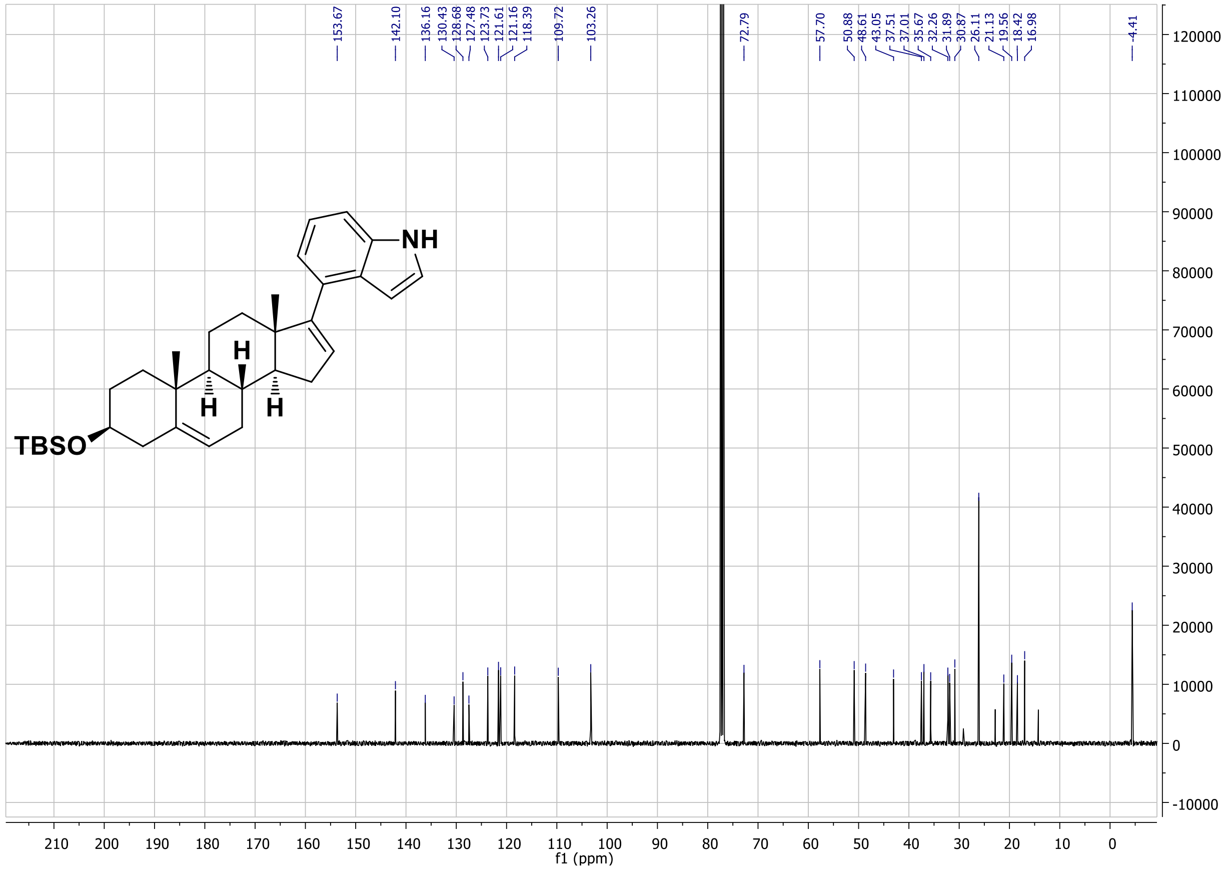
153.67
142.10
136.16
130.43
128.68
127.48
123.73
121.61
121.16
118.39
109.72
103.26
72.79
57.70
50.88
48.61
43.05
37.51
37.01
35.67
32.26
31.89
30.87
26.11
21.13
19.56
18.42
16.98
-4.41
NH
H
H
H
TBSO
f1 (ppm)

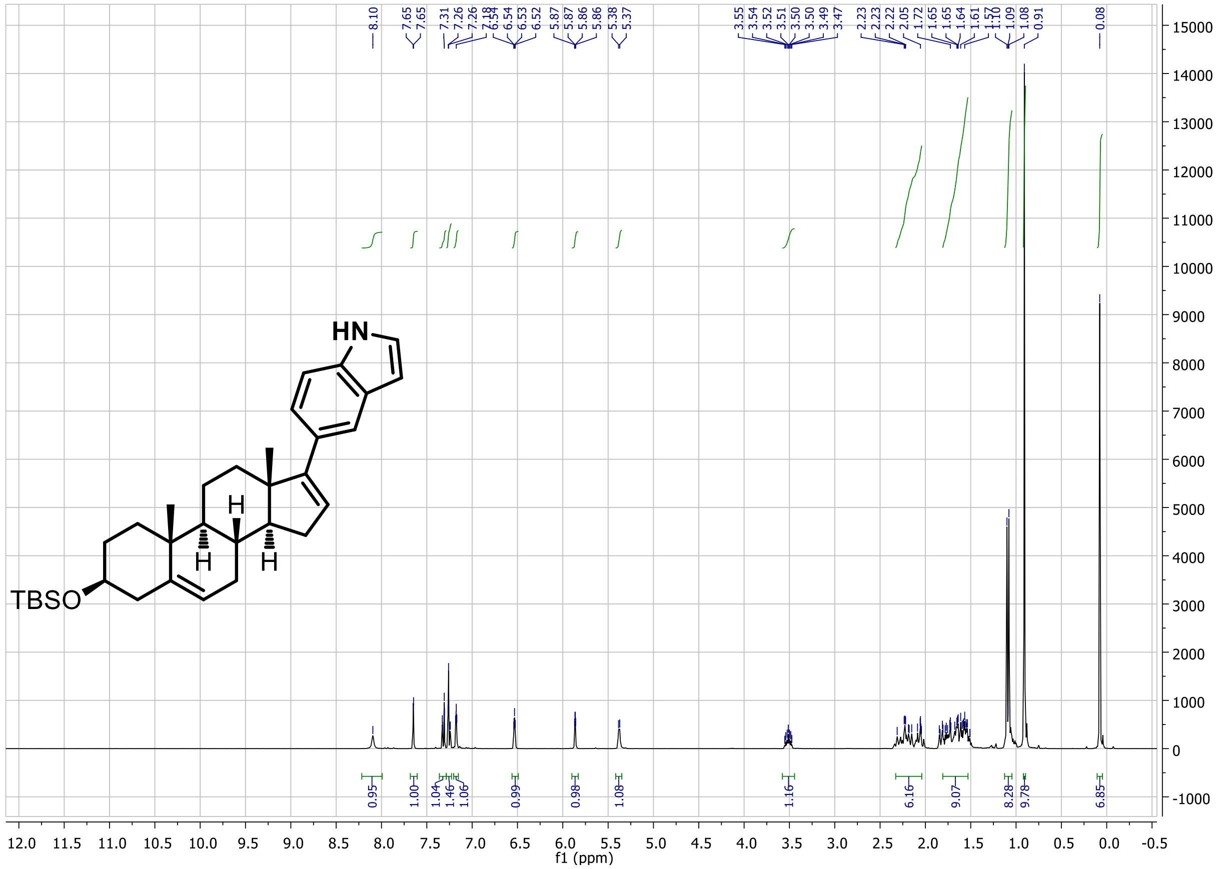
8.10
7.65
7.65
7.31
7.26
7.26
7.18
6.54
6.53
6.52
5.87
5.87
5.86
5.86
5.38
5.37
3.55
3.54
3.52
3.51
3.50
3.50
3.49
3.47
2.23
2.23
2.22
2.05
1.72
1.65
1.65
1.64
1.61
1.57
1.10
1.09
1.08
0.91
0.08
HN
TBSO
H
H
H
0.95
1.00
1.04
1.46
1.06
0.99
0.98
1.08
1.16
6.16
9.07
8.28
9.78
6.85
f1 (ppm)

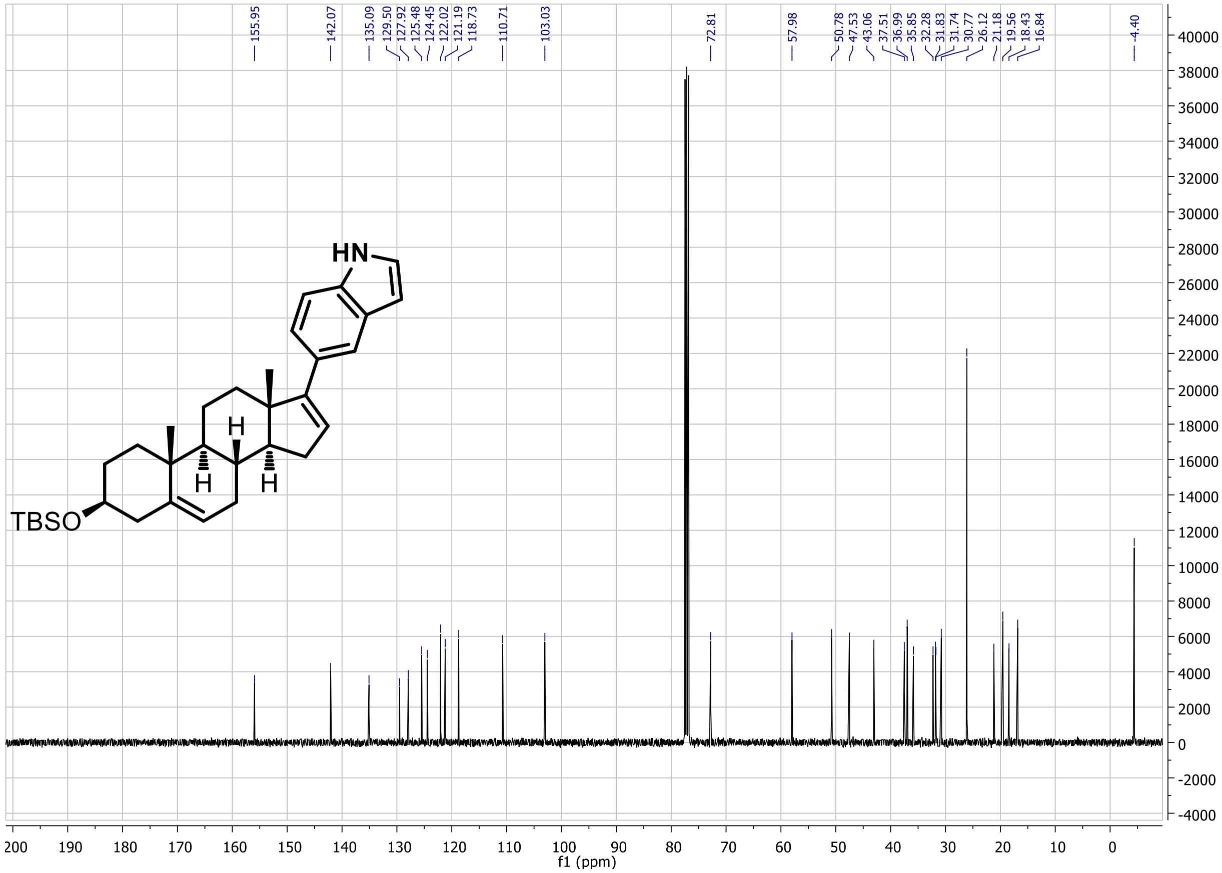
155.95
142.07
135.09
129.50
127.92
125.48
124.45
122.02
121.19
118.73
110.71
103.03
72.81
57.98
50.78
47.53
43.06
37.51
36.99
35.85
32.28
31.83
31.74
30.77
26.12
21.18
19.56
18.43
16.84
-4.40
HN
TBSO
H
H
H
f1 (ppm)

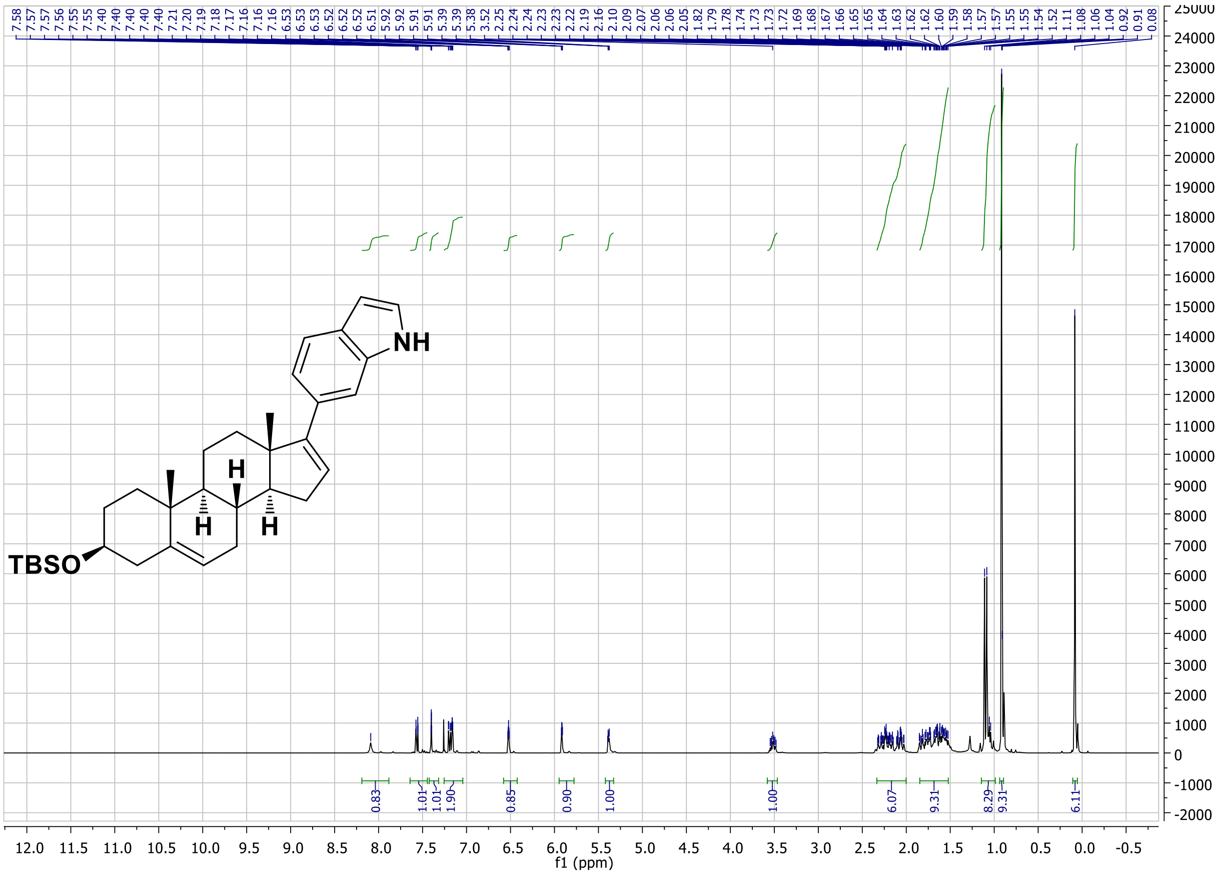
NH
H
H
H
TBSO
f1 (ppm)

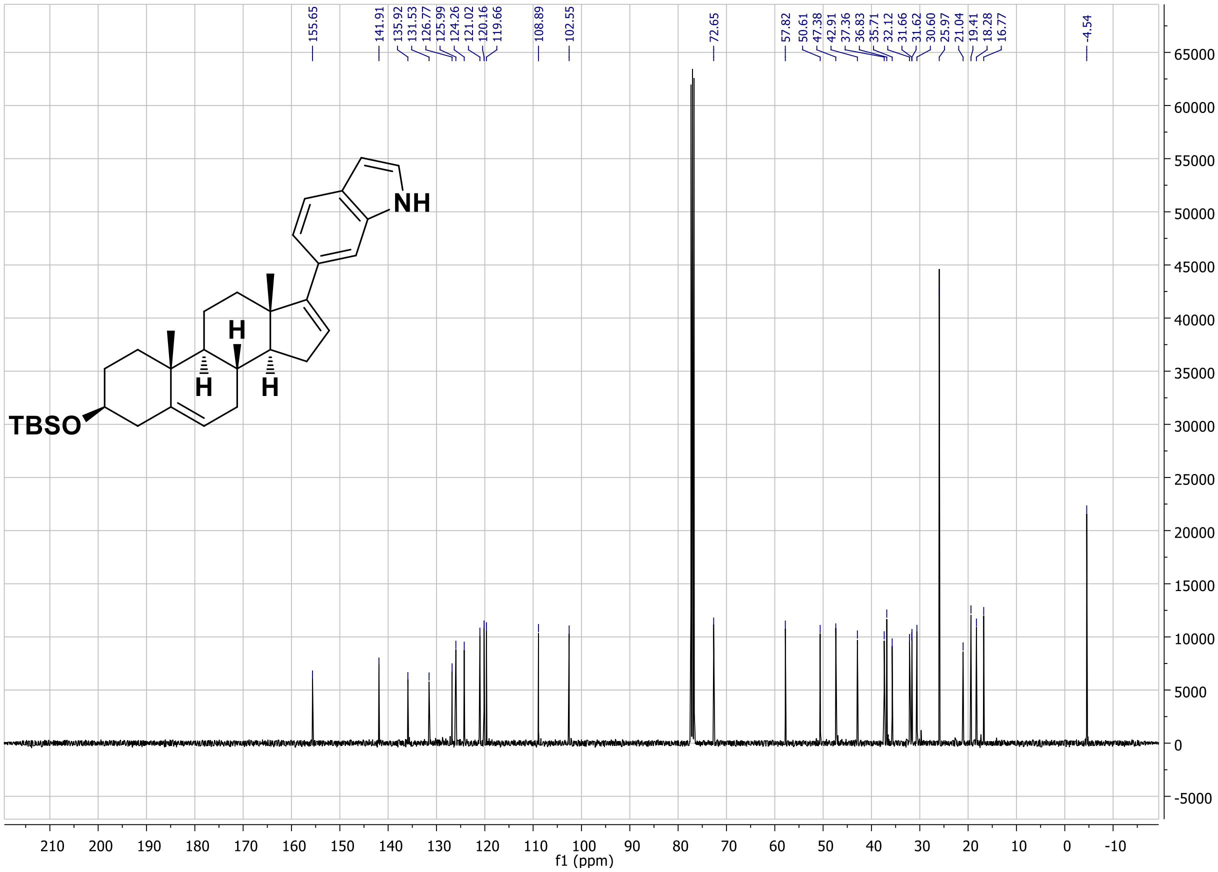
155.65
141.91
135.92
131.53
126.77
125.99
124.26
121.02
120.16
119.66
108.89
102.55
72.65
57.82
50.61
47.38
42.91
37.36
36.83
35.71
32.12
31.66
31.62
30.60
25.97
21.04
19.41
18.28
16.77
-4.54
NH
H
H
H
TBSO
f1 (ppm)

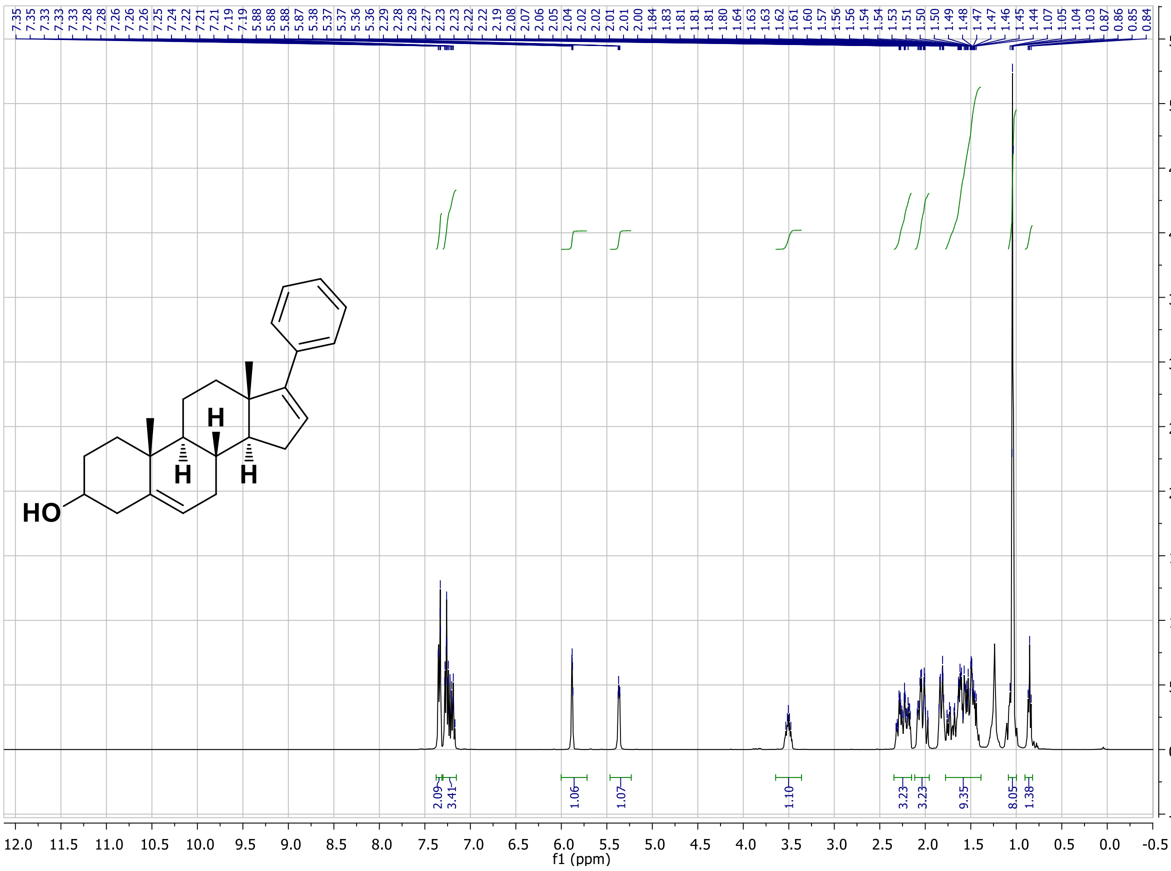

7.35 7.35 7.33 7.33 7.33 7.28 7.28 7.26 7.26 7.26 7.25 7.24 7.22 7.21 7.21 7.19 5.88 5.88 5.88 5.87 5.38 5.37 5.37 5.36 5.36 2.29 2.28 2.28 2.27 2.23 2.23 2.22 2.22 2.19 2.08 2.07 2.06 2.05 2.04 2.02 2.02 2.01 2.00 1.84 1.83 1.83 1.81 1.81 1.80 1.64 1.63 1.63 1.62 1.61 1.60 1.57 1.56 1.56 1.54 1.54 1.53 1.51 1.50 1.50 1.49 1.48 1.47 1.47 1.46 1.45 1.44 1.07 1.05 1.04 1.03 0.87 0.86 0.85 0.84
HO
H
H
H
2.09
3.41
1.06
1.07
1.10
3.23
3.23
9.35
8.05
1.38
f1 (ppm)

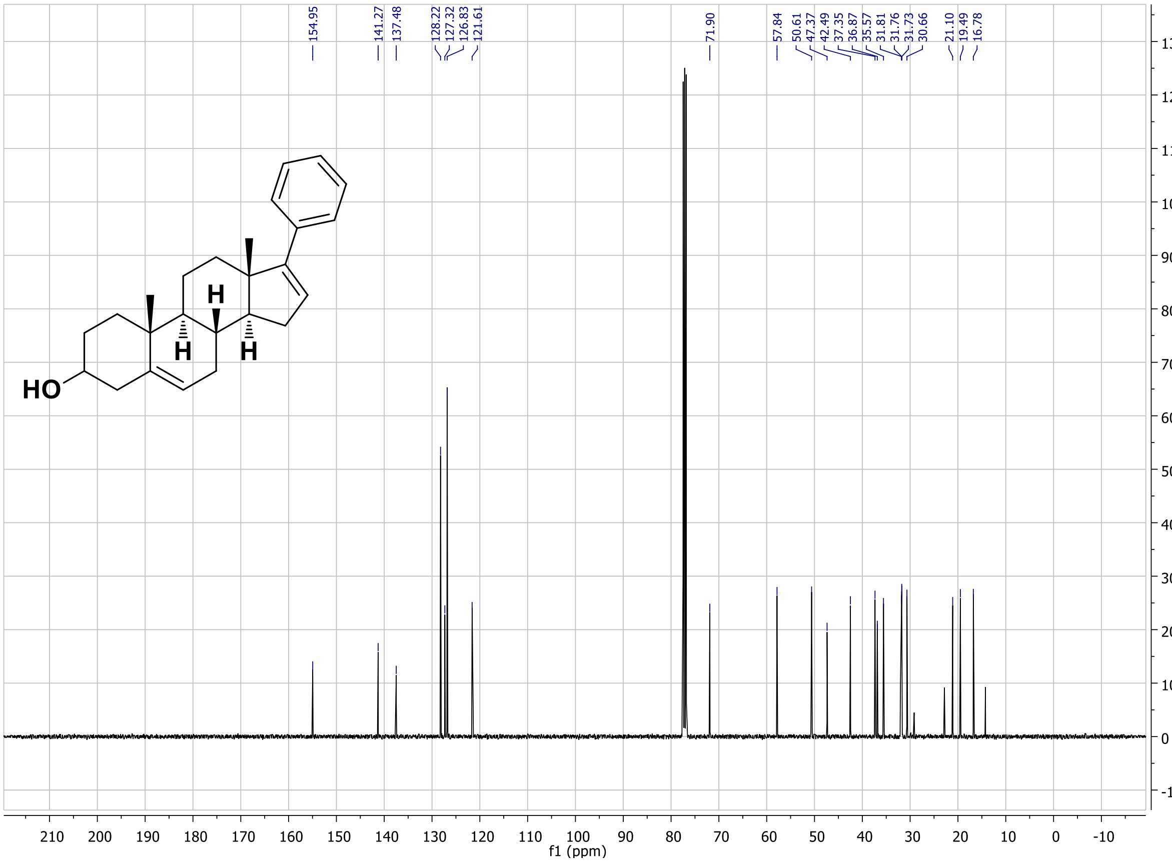

154.95
141.27
137.48
128.22 127.32 126.83 121.61
71.90
57.84 50.61 47.37 42.49 37.35 36.87 35.57 31.81 31.76 31.73 30.66 21.10 19.49 16.78
HO
H
H
H
f1 (ppm)

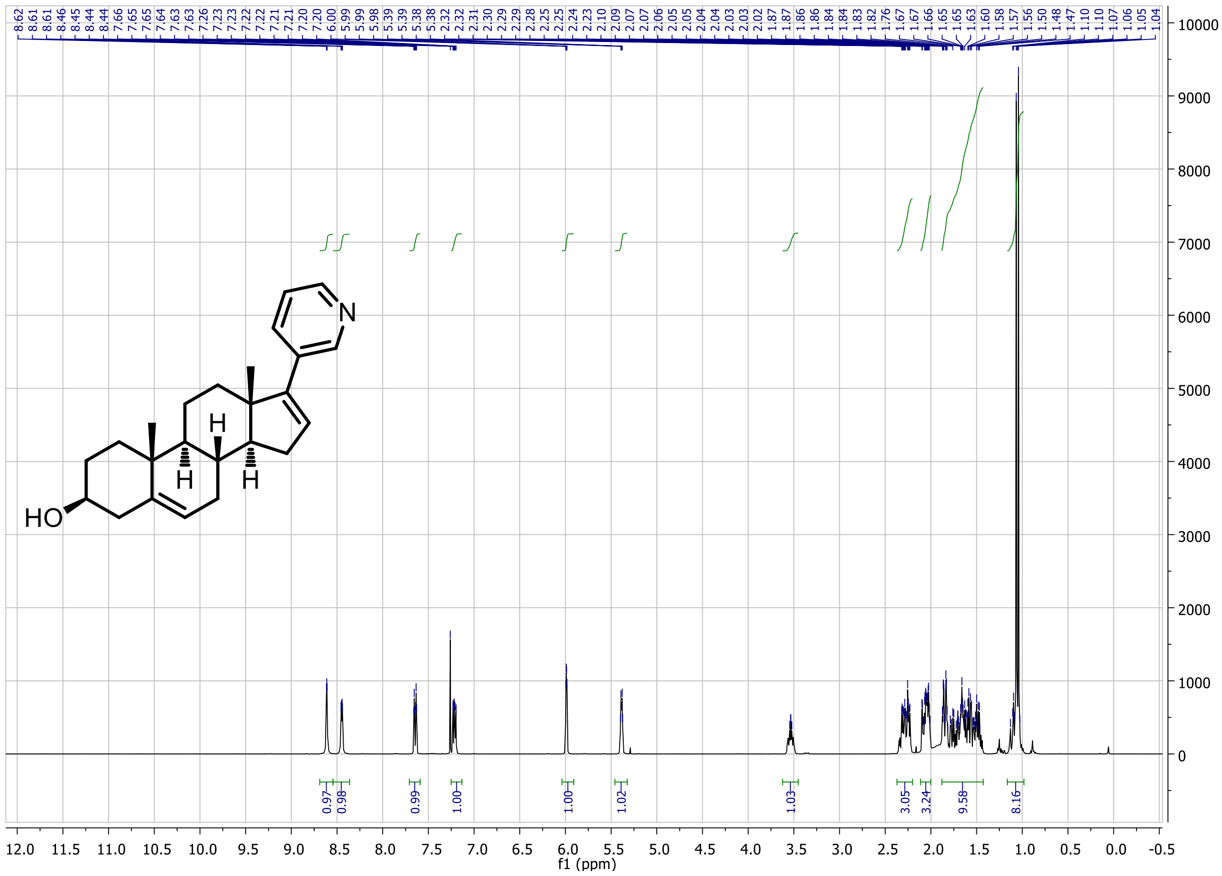
8.62 8.61 8.61 8.46 8.45 8.44 8.44 7.66 7.65 7.65 7.64 7.63 7.63 7.26 7.23 7.23 7.22 7.22 7.21 7.21 7.20 7.20 6.00 5.99 5.99 5.98 5.98 5.39 5.39 5.38 5.38 2.32 2.32 2.31 2.30 2.29 2.29 2.28 2.25 2.25 2.24 2.23 2.10 2.09 2.07 2.07 2.06 2.05 2.05 2.04 2.04 2.03 2.03 2.02 1.87 1.87 1.86 1.86 1.84 1.84 1.83 1.82 1.76 1.67 1.67 1.66 1.65 1.65 1.63 1.60 1.58 1.57 1.56 1.50 1.48 1.47 1.10 1.10 1.07 1.06 1.05 1.04
N
H
H
H
HO
0.97
0.98
0.99
1.00
1.00
1.02
1.03
3.05
3.24
9.58
8.16
12.0 11.5 11.0 10.5 10.0 9.5 9.0 8.5 8.0 7.5 7.0 6.5 6.0 5.5 5.0 4.5 4.0 3.5 3.0 2.5 2.0 1.5 1.0 0.5 0.0 -0.5
f1 (ppm)
10000 9000 8000 7000 6000 5000 4000 3000 2000 1000 0

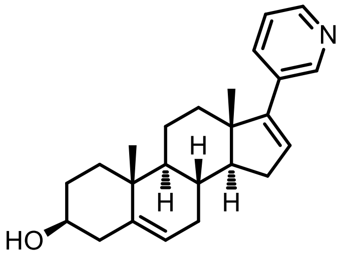
N
H
H
H
HO

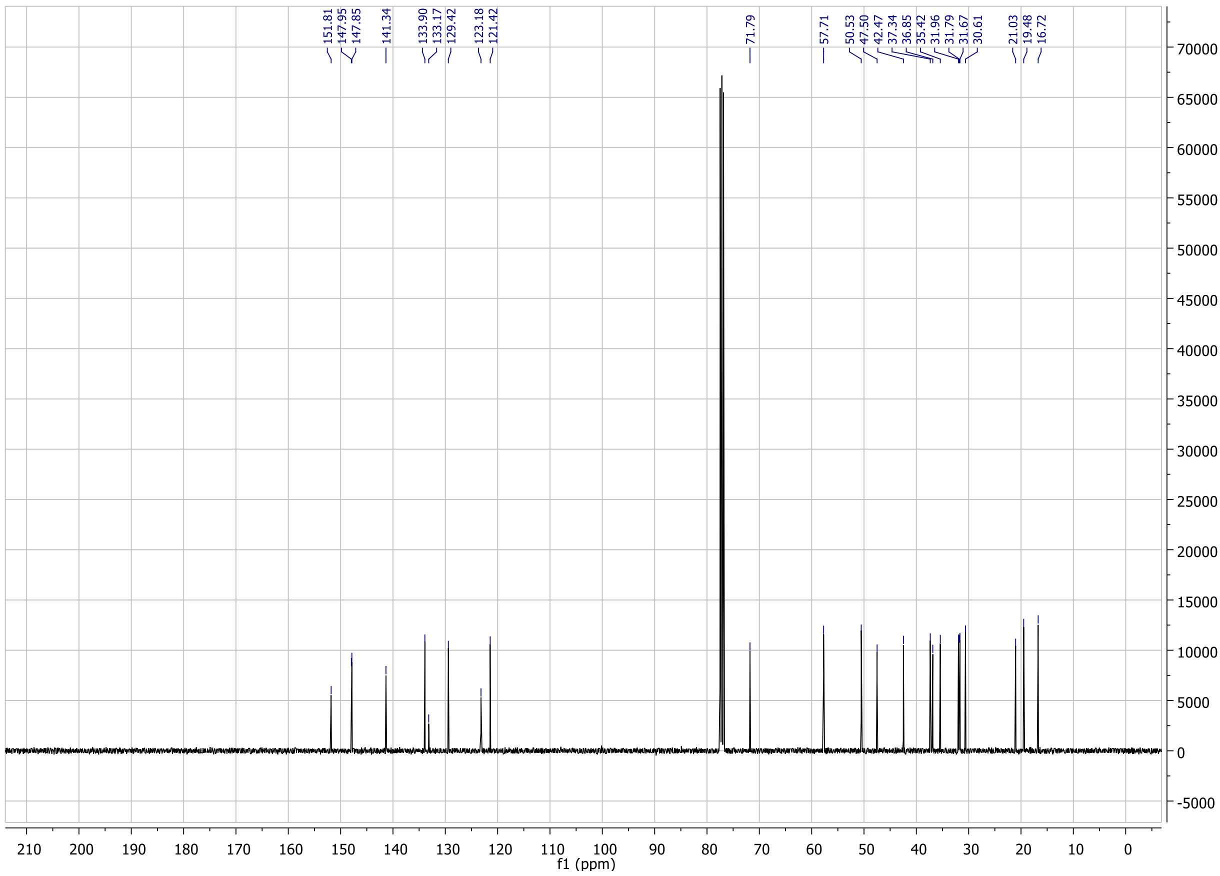
151.81
147.95
147.85
141.34
133.90
133.17
129.42
123.18
121.42
71.79
57.71
50.53
47.50
42.47
37.34
36.85
35.42
31.96
31.79
31.67
30.61
21.03
19.48
16.72
f1 (ppm)

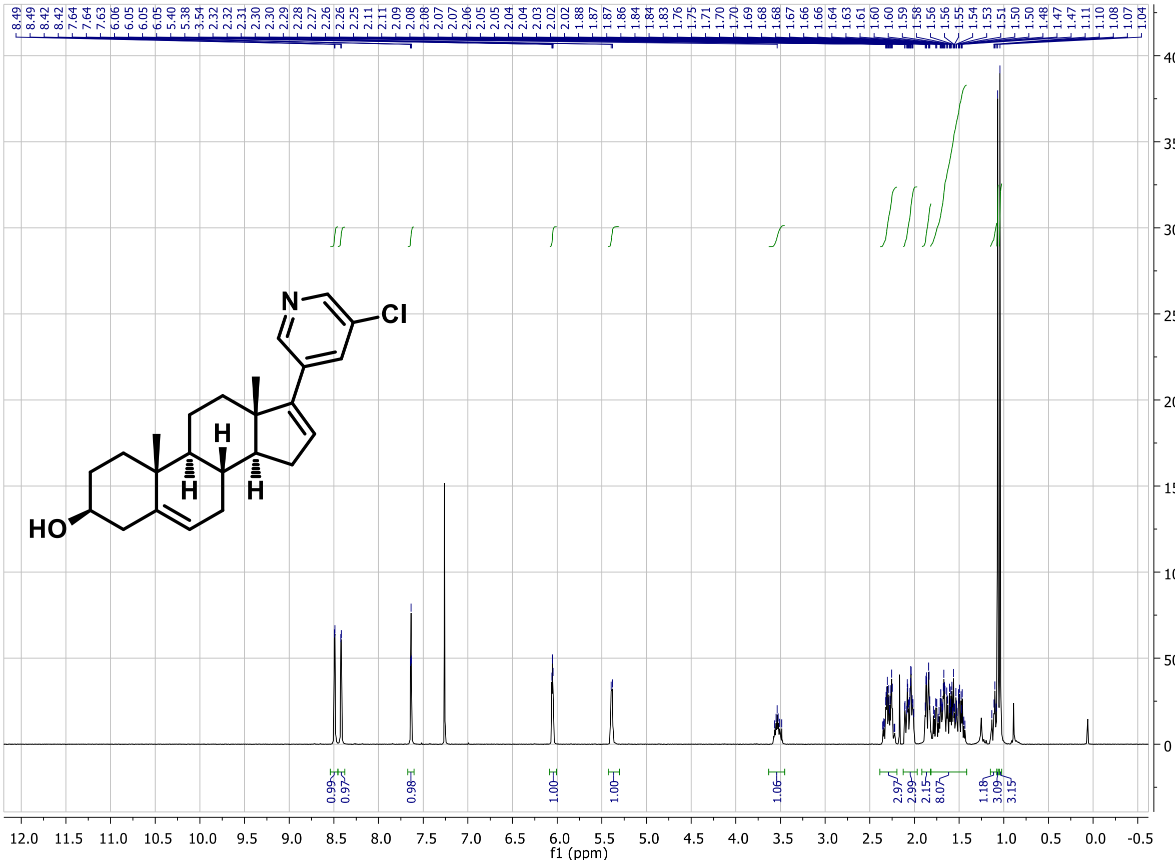
8.49 8.49 8.42 8.42 7.64 7.63 6.06 6.05 6.05 6.05 5.40 5.38 3.54 2.32 2.32 2.31 2.30 2.30 2.29 2.28 2.27 2.26 2.26 2.25 2.11 2.11 2.09 2.08 2.08 2.07 2.07 2.06 2.05 2.05 2.04 2.04 2.03 2.02 2.02 1.88 1.87 1.86 1.84 1.84 1.83 1.76 1.75 1.71 1.70 1.70 1.69 1.68 1.68 1.67 1.66 1.66 1.64 1.63 1.61 1.60 1.60 1.59 1.58 1.56 1.56 1.55 1.54 1.53 1.51 1.50 1.48 1.47 1.47 1.11 1.10 1.08 1.07 1.04
N
Cl
H
H
H
HO
0.99 0.97 0.98 1.00 1.00 1.06 2.97 2.99 2.15 8.07 1.18 3.09 3.15
4000 3500 3000 2500 2000 1500 1000 500 0
12.0 11.5 11.0 10.5 10.0 9.5 9.0 8.5 8.0 7.5 7.0 6.5 6.0 5.5 5.0 4.5 4.0 3.5 3.0 2.5 2.0 1.5 1.0 0.5 0.0 -0.5
f1 (ppm)

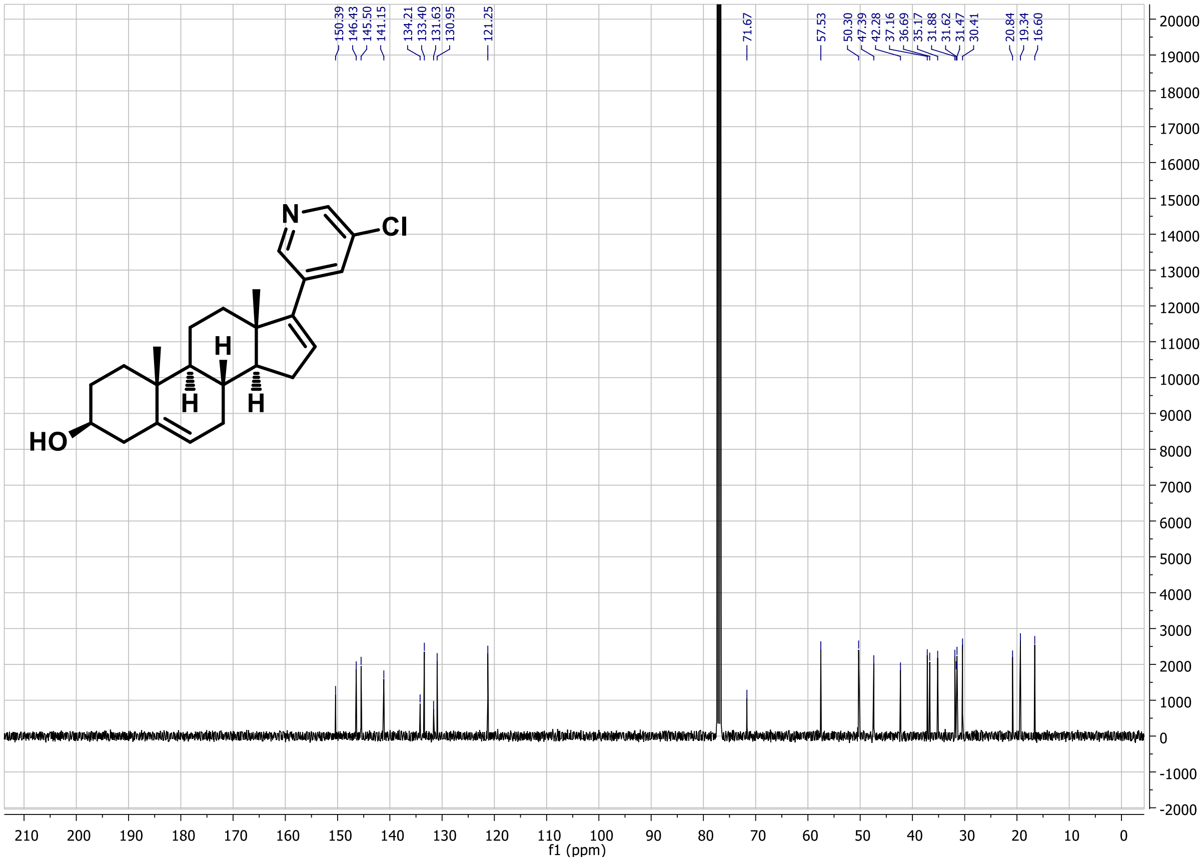
150.39 146.43 145.50 141.15 134.21 133.40 131.63 130.95 121.25 71.67 57.53 50.30 47.39 42.28 37.16 36.69 35.17 31.88 31.62 31.47 30.41 20.84 19.34 16.60
N
Cl
H
H
H
HO
20000 19000 18000 17000 16000 15000 14000 13000 12000 11000 10000 9000 8000 7000 6000 5000 4000 3000 2000 1000 0 -1000 -2000
210 200 190 180 170 160 150 140 130 120 110 100 90 80 70 60 50 40 30 20 10 0
f1 (ppm)

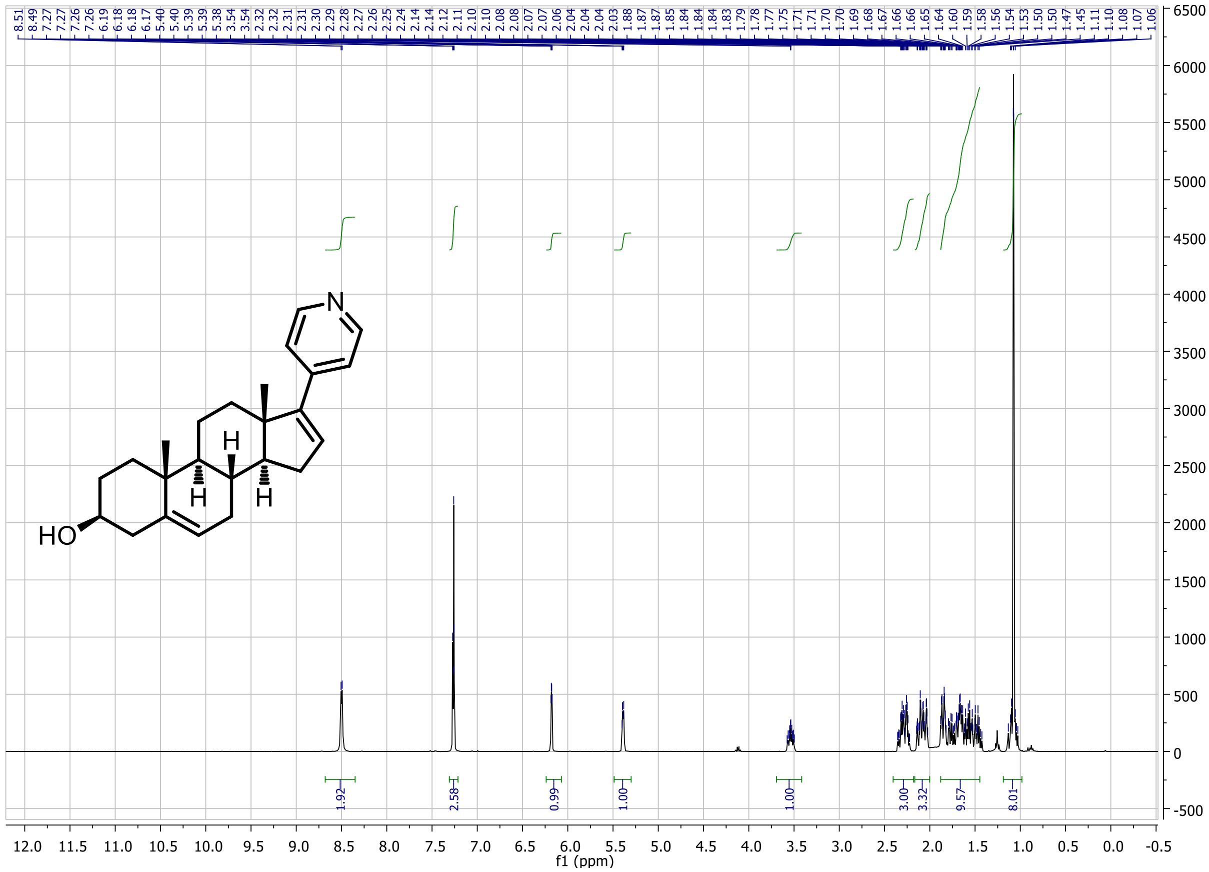
8.51 8.49 7.27 7.27 7.26 7.26 6.19 6.18 6.18 6.17 5.40 5.40 5.39 5.39 5.38 3.54 3.54 2.32 2.32 2.31 2.30 2.29 2.28 2.27 2.26 2.25 2.24 2.14 2.14 2.12 2.11 2.10 2.10 2.08 2.08 2.07 2.07 2.06 2.04 2.04 2.03 1.88 1.87 1.87 1.85 1.84 1.84 1.84 1.83 1.79 1.78 1.77 1.75 1.71 1.71 1.70 1.69 1.68 1.67 1.66 1.65 1.64 1.60 1.59 1.58 1.56 1.54 1.53 1.50 1.50 1.47 1.45 1.11 1.10 1.08 1.07 1.06
1.92
2.58
0.99
1.00
1.00
3.00
3.32
9.57
8.01
HO
H
H
H
N
f1 (ppm)

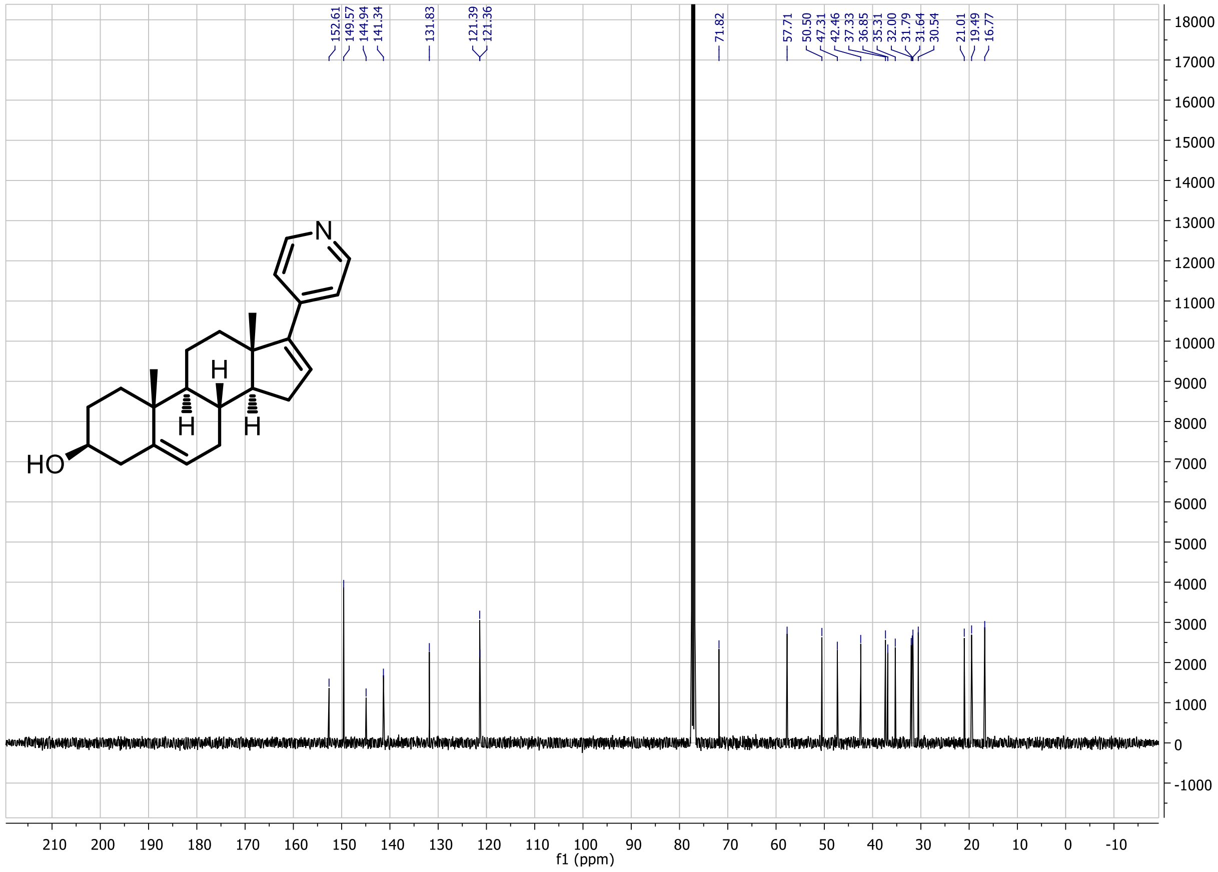
152.61 149.57 144.94 141.34 131.83 121.39 121.36 71.82 57.71 50.50 47.31 42.46 37.33 36.85 35.31 32.00 31.79 31.64 30.54 21.01 19.49 16.77
HO
H
H
H
N
f1 (ppm)

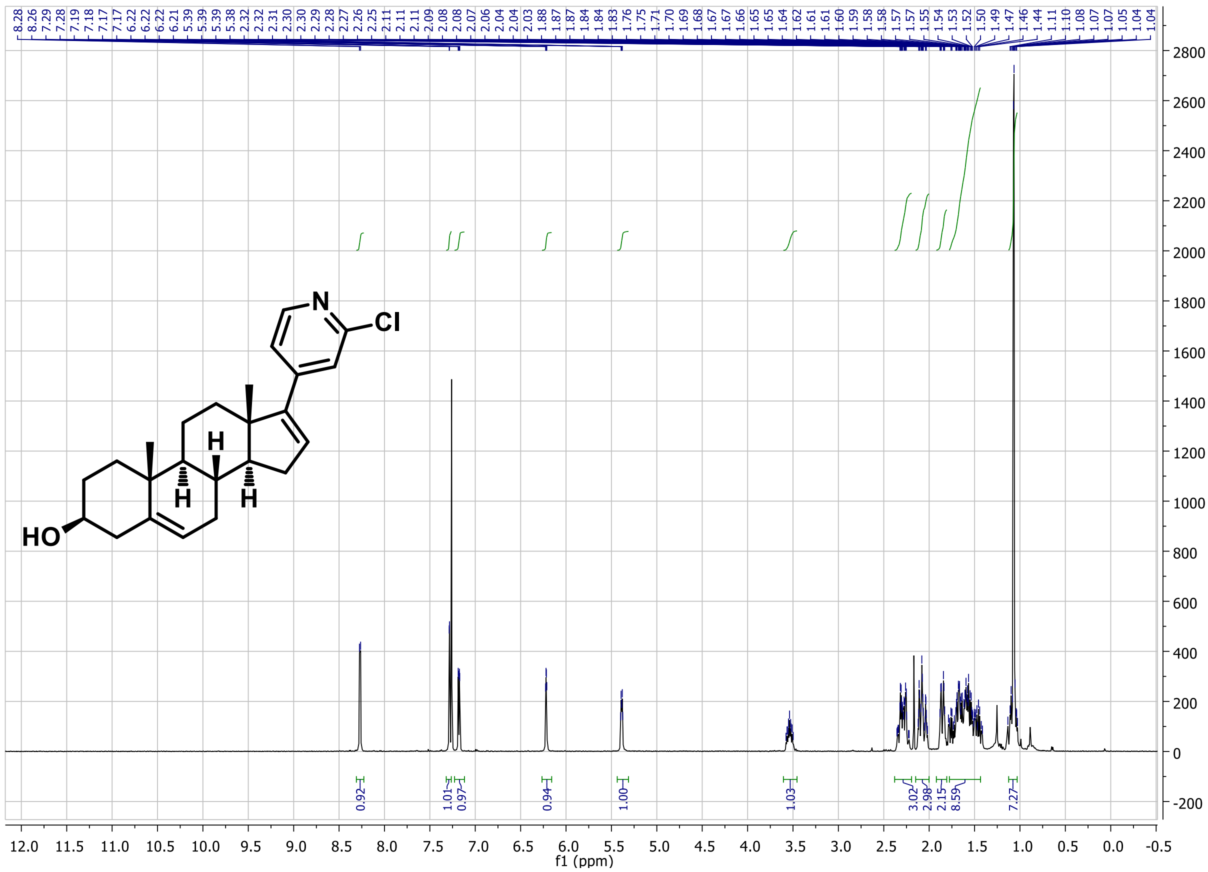

8.28 8.26 7.29 7.28 7.19 7.18 7.17 6.22 6.22 6.21 5.39 5.39 5.39 5.38 2.32 2.32 2.31 2.30 2.30 2.29 2.28 2.27 2.26 2.25 2.11 2.11 2.11 2.09 2.08 2.08 2.07 2.06 2.04 2.04 2.03 1.88 1.87 1.87 1.84 1.84 1.83 1.76 1.75 1.71 1.70 1.69 1.68 1.67 1.67 1.66 1.65 1.65 1.64 1.62 1.61 1.61 1.60 1.59 1.58 1.58 1.57 1.57 1.55 1.54 1.53 1.52 1.50 1.49 1.47 1.46 1.44 1.11 1.10 1.08 1.07 1.07 1.05 1.04 1.04
N
Cl
H
H
H
HO
0.92 1.01 0.97 0.94 1.00 1.03 3.02 2.98 2.15 8.59 7.27
f1 (ppm)

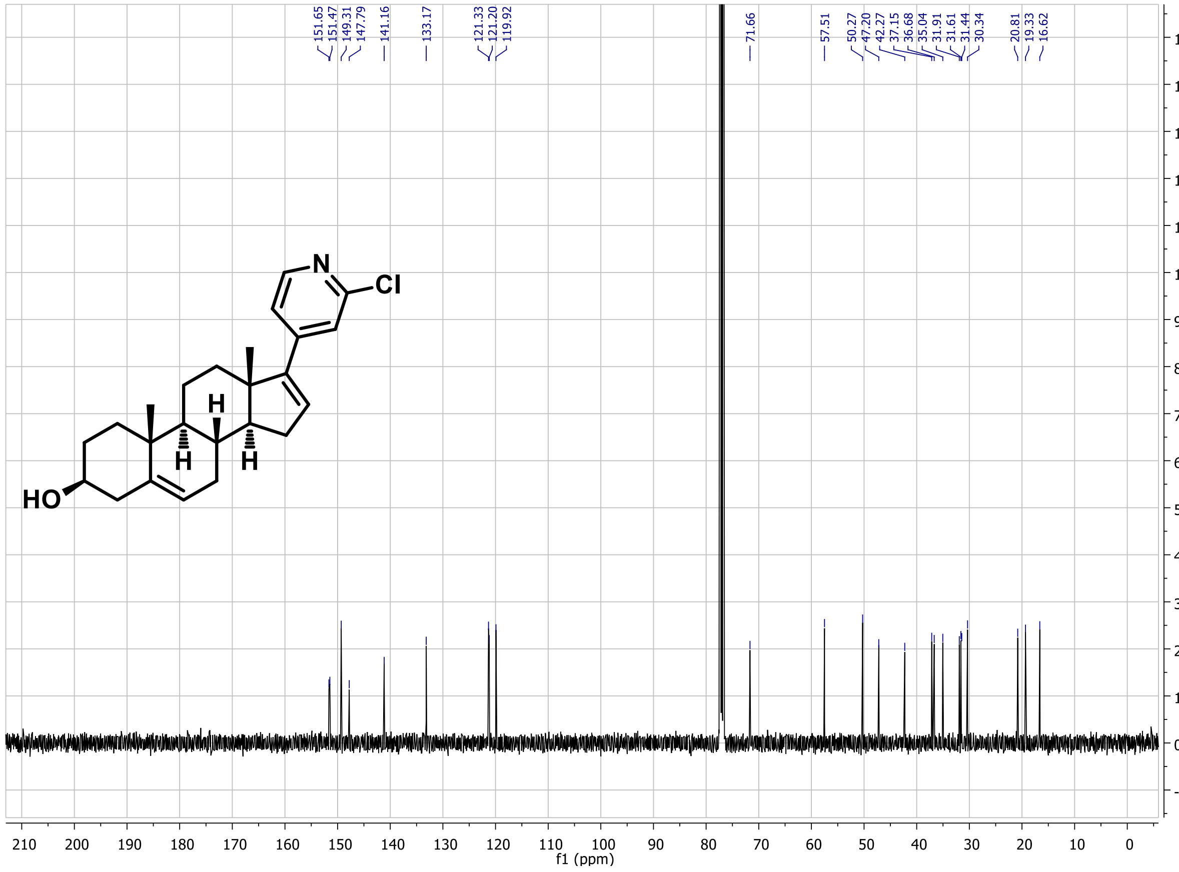

151.65 151.47 149.31 147.79 141.16 133.17 121.33 121.20 119.92 71.66 57.51 50.27 47.20 42.27 37.15 36.68 35.04 31.91 31.61 31.44 30.34 20.81 19.33 16.62
N
Cl
H
H
H
HO
f1 (ppm)

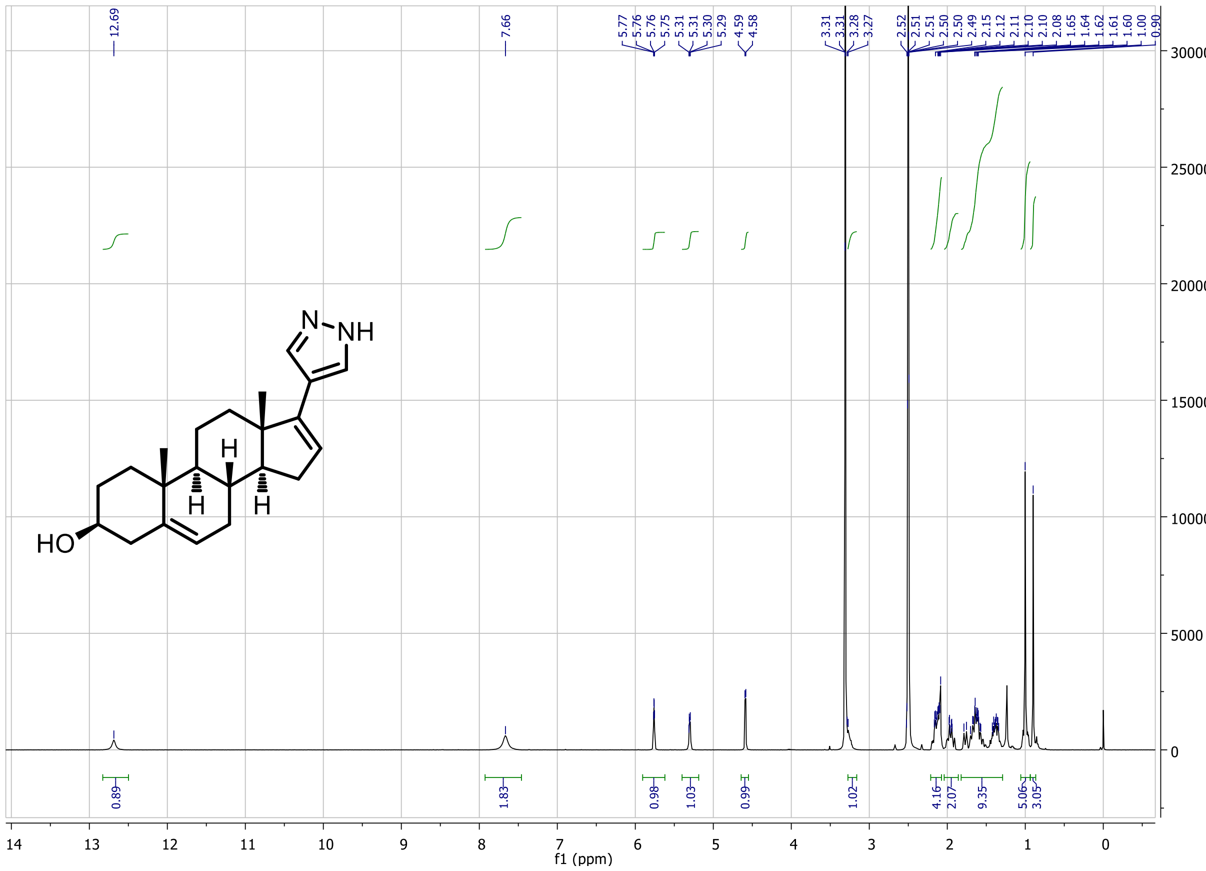

12.69
7.66
5.77
5.76
5.76
5.75
5.31
5.31
5.30
5.29
4.59
4.58
3.31
3.31
3.28
3.27
2.52
2.51
2.51
2.50
2.50
2.49
2.15
2.12
2.11
2.10
2.10
2.08
1.65
1.64
1.62
1.61
1.60
1.00
0.90
0.89
1.83
0.98
1.03
0.99
1.02
4.16
2.07
9.35
5.06
3.05
N
NH
H
H
H
HO
f1 (ppm)

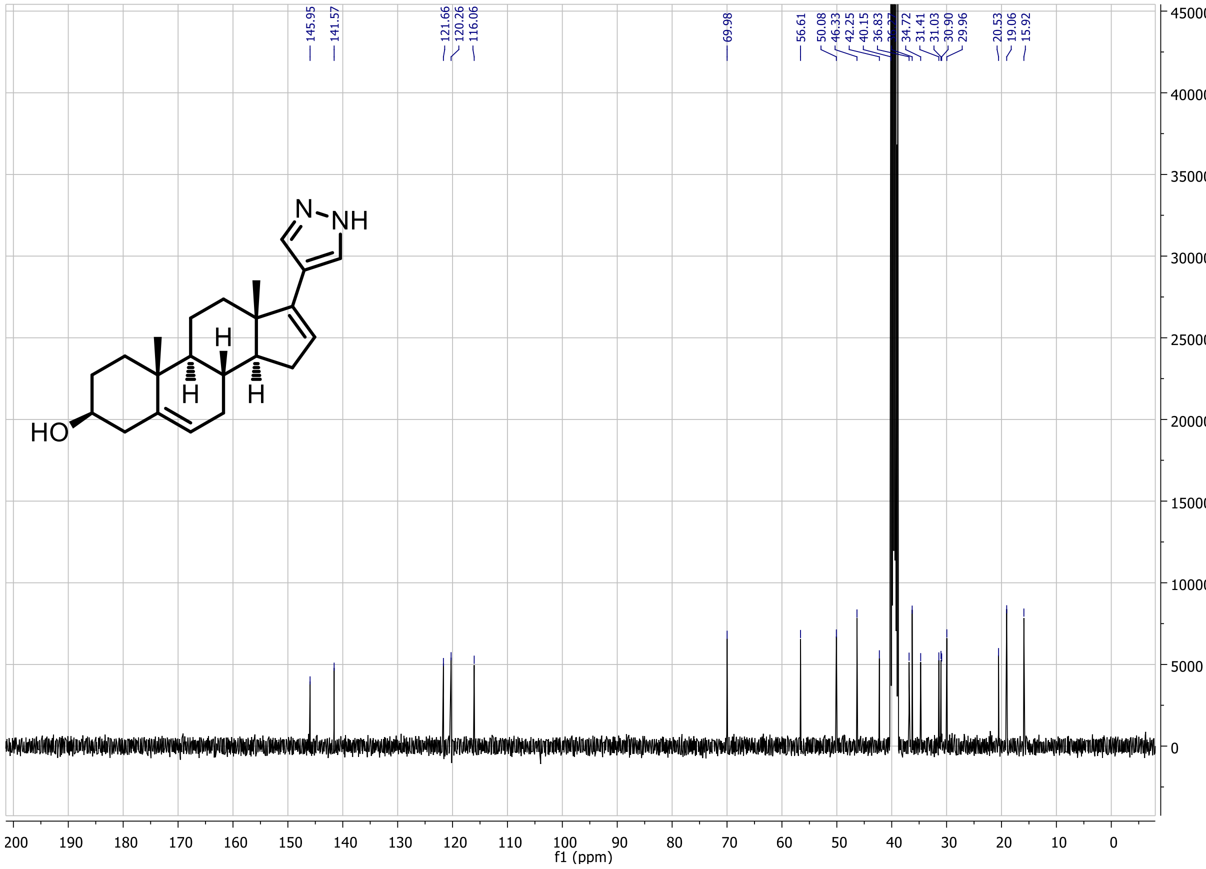

145.95
141.57
121.66
120.26
116.06
69.98
56.61
50.08
46.33
42.25
40.15
36.83
34.72
31.41
31.03
30.90
29.96
20.53
19.06
15.92
N
NH
H
H
H
HO
f1 (ppm)

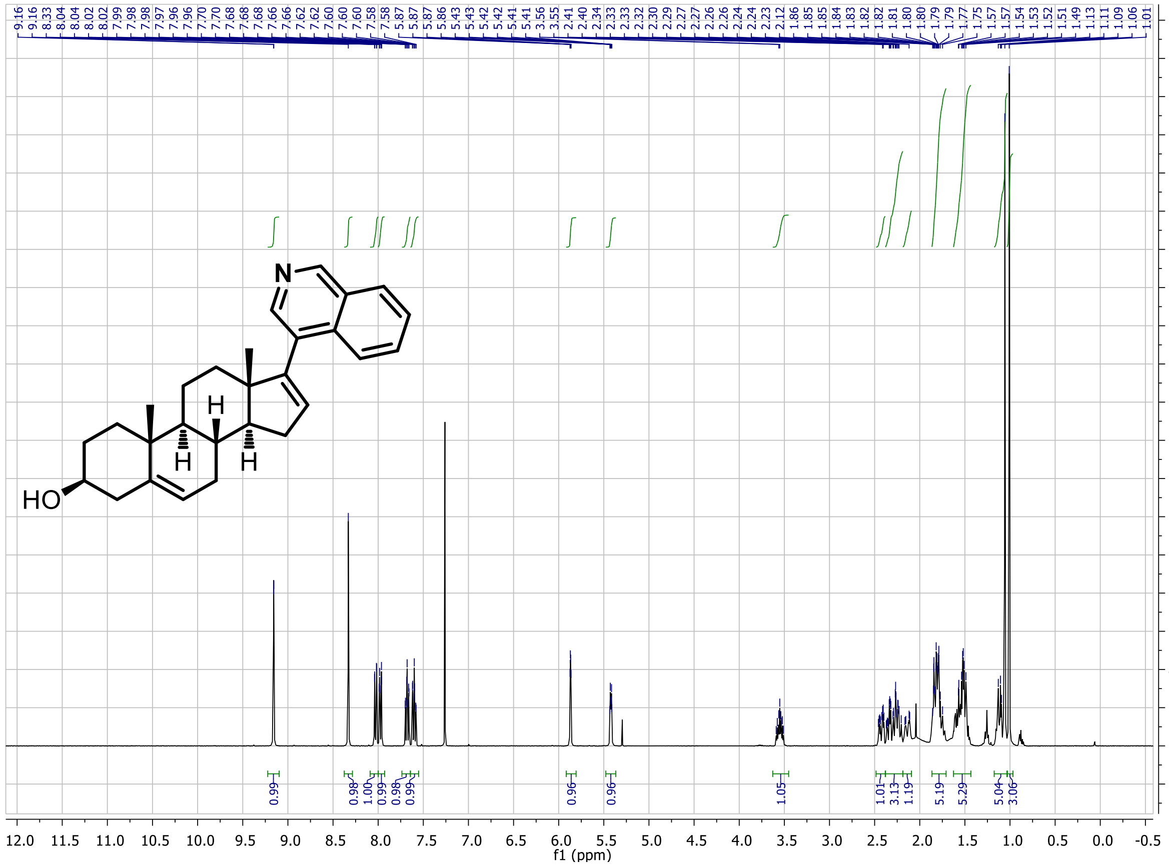

9.16
9.16
8.33
8.04
8.04
8.02
8.02
7.99
7.98
7.98
7.97
7.96
7.96
7.70
7.70
7.68
7.68
7.66
7.66
7.62
7.62
7.60
7.60
7.60
7.58
7.58
5.87
5.87
5.86
5.43
5.43
5.42
5.42
5.41
5.41
3.56
3.55
2.41
2.40
2.34
2.33
2.33
2.32
2.30
2.29
2.27
2.27
2.26
2.26
2.24
2.24
2.24
2.23
2.12
1.86
1.85
1.85
1.84
1.83
1.82
1.81
1.80
1.80
1.79
1.79
1.77
1.75
1.57
1.57
1.54
1.53
1.52
1.51
1.49
1.13
1.11
1.09
1.06
1.01
0.99
0.98
1.00
0.99
0.98
0.99
0.96
0.96
1.05
1.01
3.13
1.19
5.19
5.29
5.04
3.06
HO
N
H
H
H
f1 (ppm)

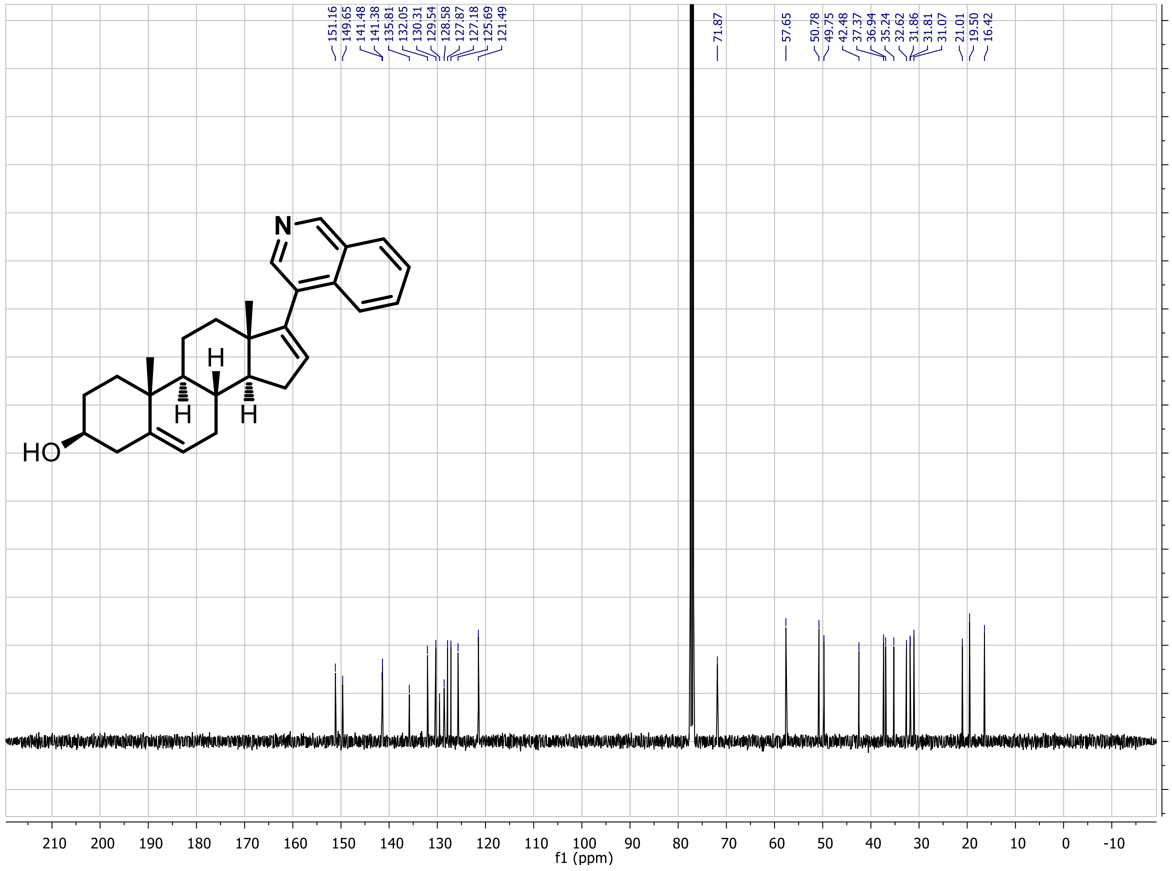

151.16
149.65
141.48
141.38
135.81
132.05
130.31
129.54
128.58
127.87
127.18
125.69
121.49
71.87
57.65
50.78
49.75
42.48
37.37
36.94
35.24
32.62
31.86
31.81
31.07
21.01
19.50
16.42
HO
N
H
H
H
f1 (ppm)

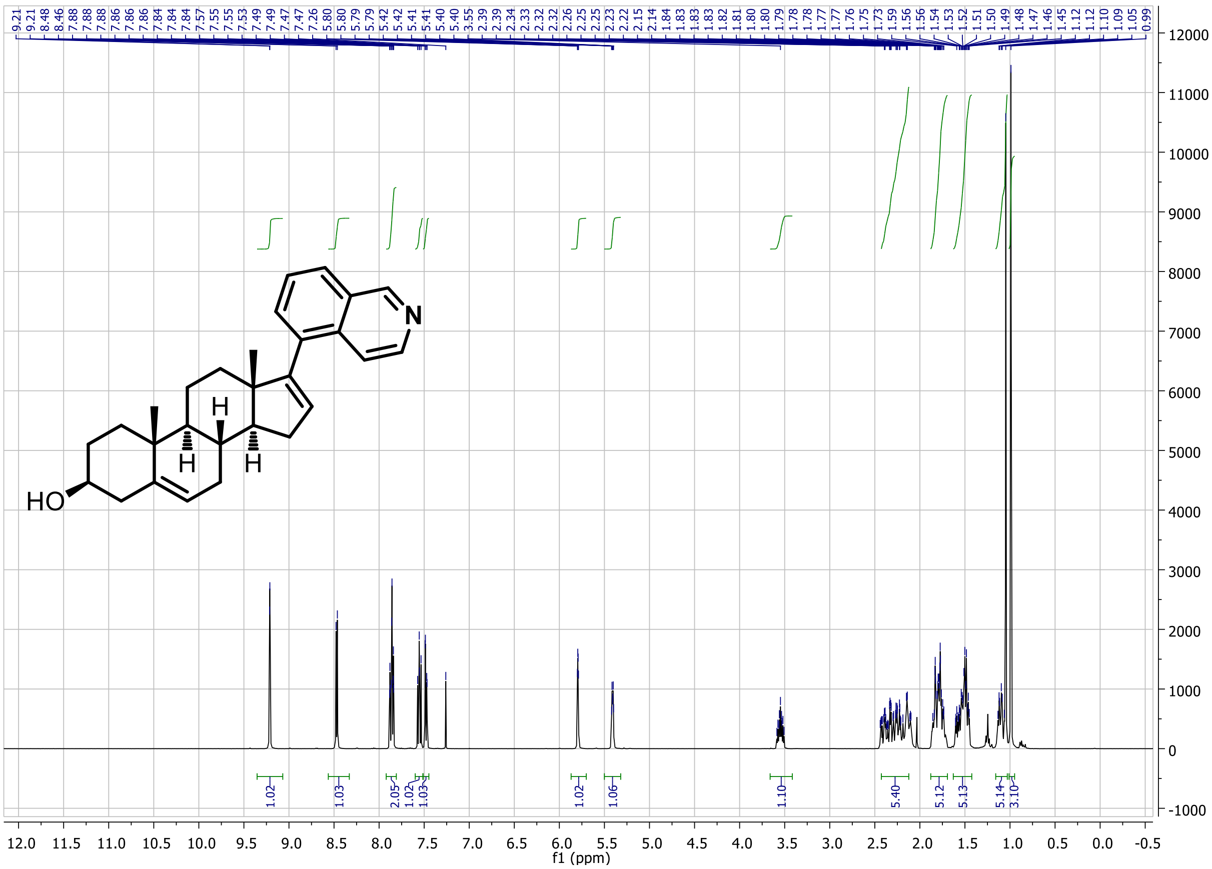
9.21 9.21 8.48 8.46 7.88 7.88 7.86 7.86 7.86 7.84 7.84 7.84 7.57 7.55 7.55 7.53 7.49 7.49 7.47 7.47 7.26 5.80 5.80 5.79 5.79 5.42 5.42 5.41 5.41 5.40 5.40 3.55 2.39 2.39 2.34 2.33 2.32 2.32 2.26 2.25 2.25 2.23 2.22 2.15 2.14 1.84 1.83 1.83 1.82 1.82 1.81 1.80 1.80 1.79 1.78 1.78 1.77 1.77 1.76 1.75 1.73 1.59 1.56 1.56 1.54 1.53 1.52 1.52 1.51 1.50 1.49 1.48 1.47 1.46 1.45 1.12 1.12 1.10 1.09 1.05 0.99
N
H
H
H
HO
1.02
1.03
2.05
1.02
1.03
1.02
1.06
1.10
5.40
5.12
5.13
5.14
3.10
f1 (ppm)
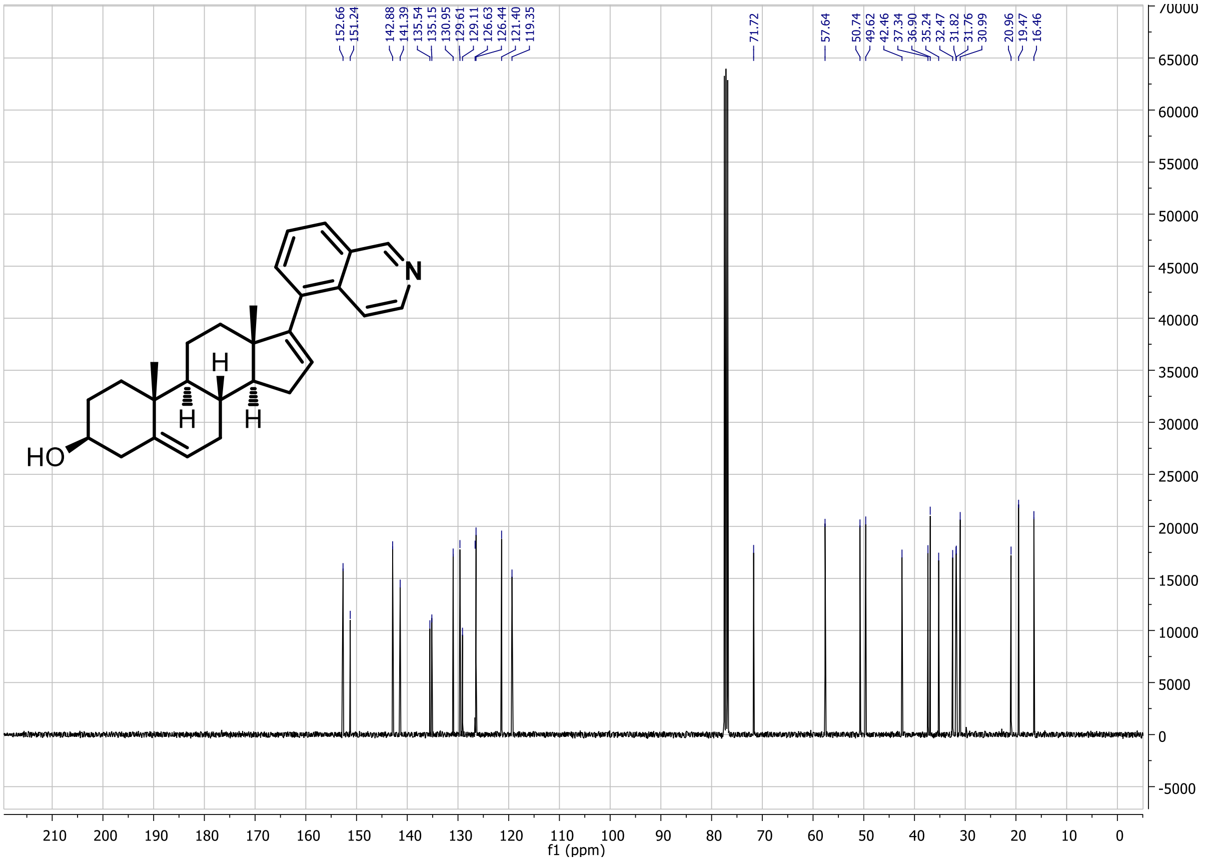
152.66 151.24 142.88 141.39 135.54 135.15 130.95 129.61 129.11 126.63 126.44 121.40 119.35
71.72
57.64 50.74 49.62 42.46 37.34 36.90 35.24 32.47 31.82 31.76 30.99 20.96 19.47 16.46
N
H
H
H
HO
f1 (ppm)

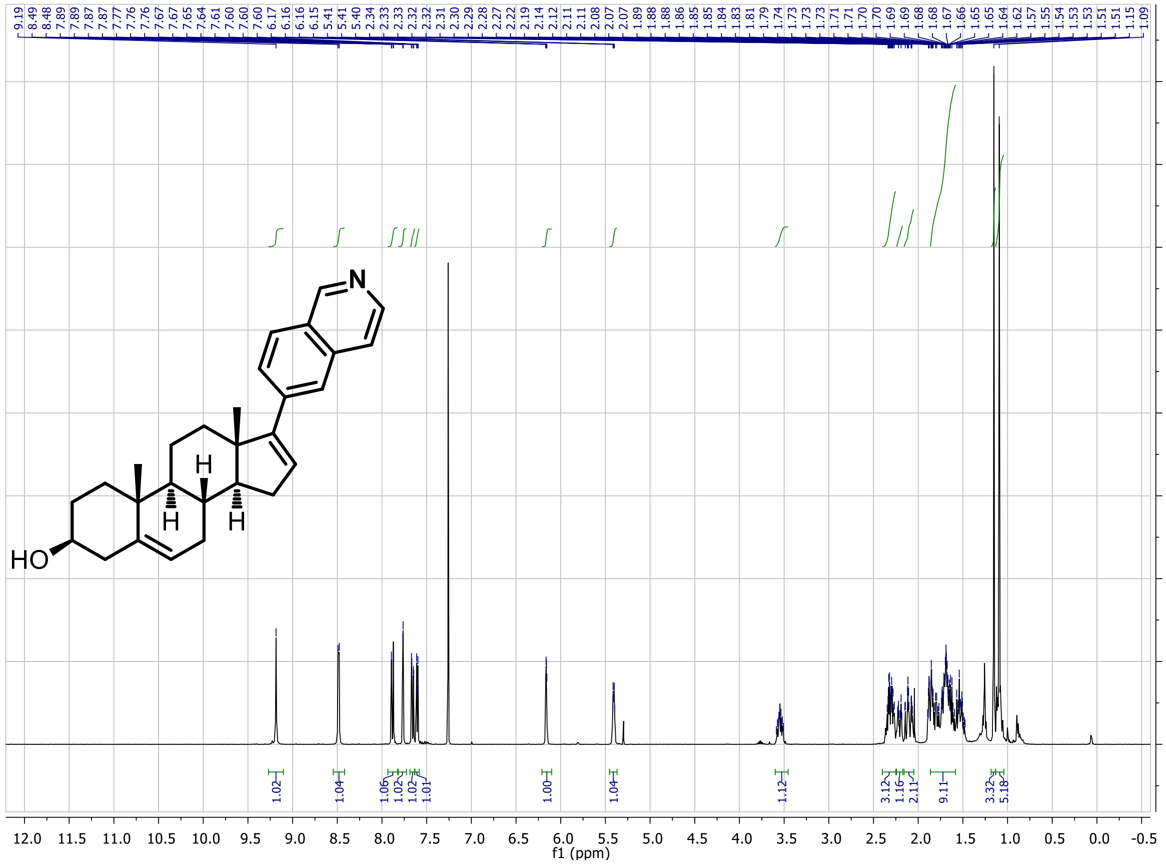
9.19 8.49 8.48 7.89 7.89 7.87 7.87 7.77 7.76 7.76 7.67 7.67 7.65 7.64 7.61 7.60 7.60 7.60 6.17 6.16 6.16 6.15 5.41 5.41 5.40 2.34 2.33 2.33 2.32 2.32 2.31 2.30 2.29 2.28 2.27 2.22 2.19 2.14 2.12 2.11 2.11 2.08 2.07 2.07 1.89 1.88 1.88 1.86 1.85 1.85 1.84 1.83 1.81 1.79 1.74 1.73 1.73 1.73 1.73 1.71 1.71 1.70 1.70 1.69 1.69 1.68 1.68 1.67 1.66 1.65 1.65 1.64 1.62 1.57 1.55 1.54 1.53 1.53 1.51 1.51 1.15 1.09
N
H
H
H
HO
1.02 1.04 1.06 1.02 1.02 1.01 1.00 1.04 1.12 3.12 1.16 2.11 9.11 3.32 5.18
f1 (ppm)

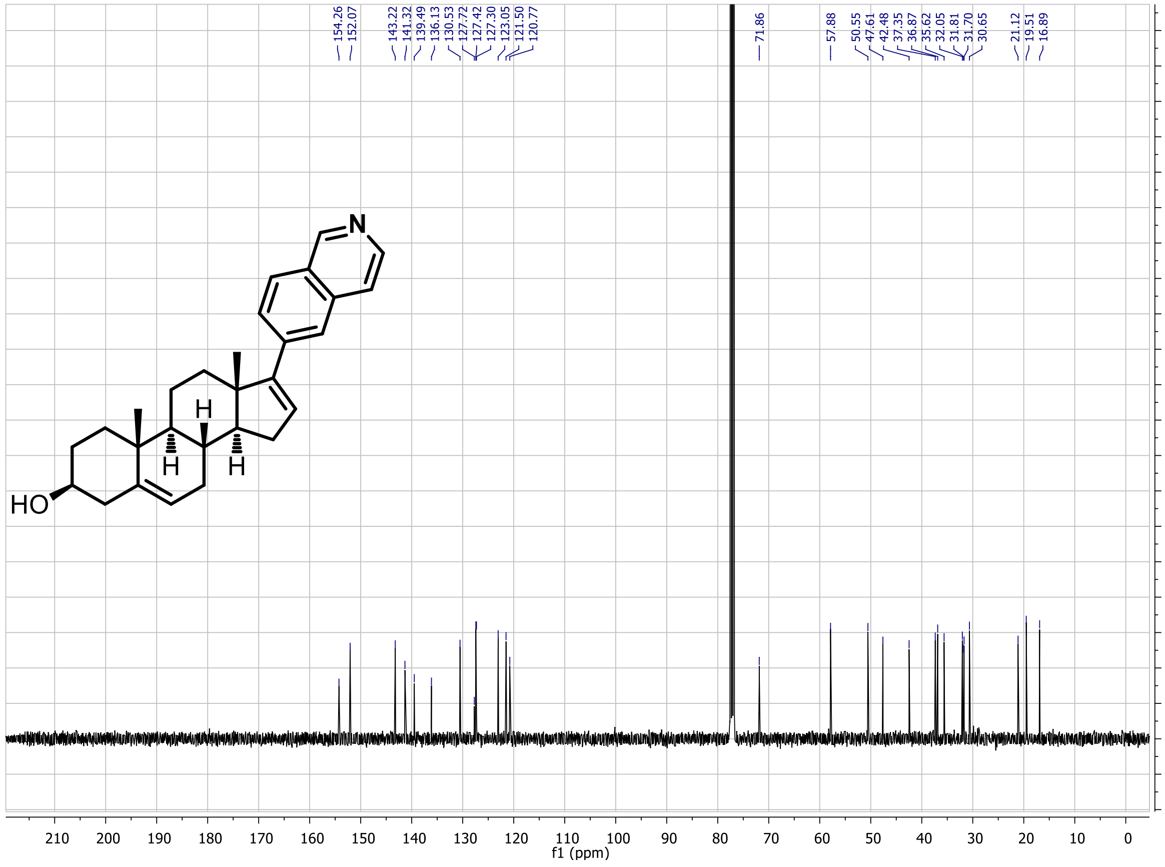
154.26 152.07 143.22 141.32 139.49 136.13 130.53 127.72 127.42 127.30 123.05 121.50 120.77 71.86 57.88 50.55 47.61 42.48 37.35 36.87 35.62 32.05 31.81 31.70 30.65 21.12 19.51 16.89
N
H
H
H
HO
f1 (ppm)

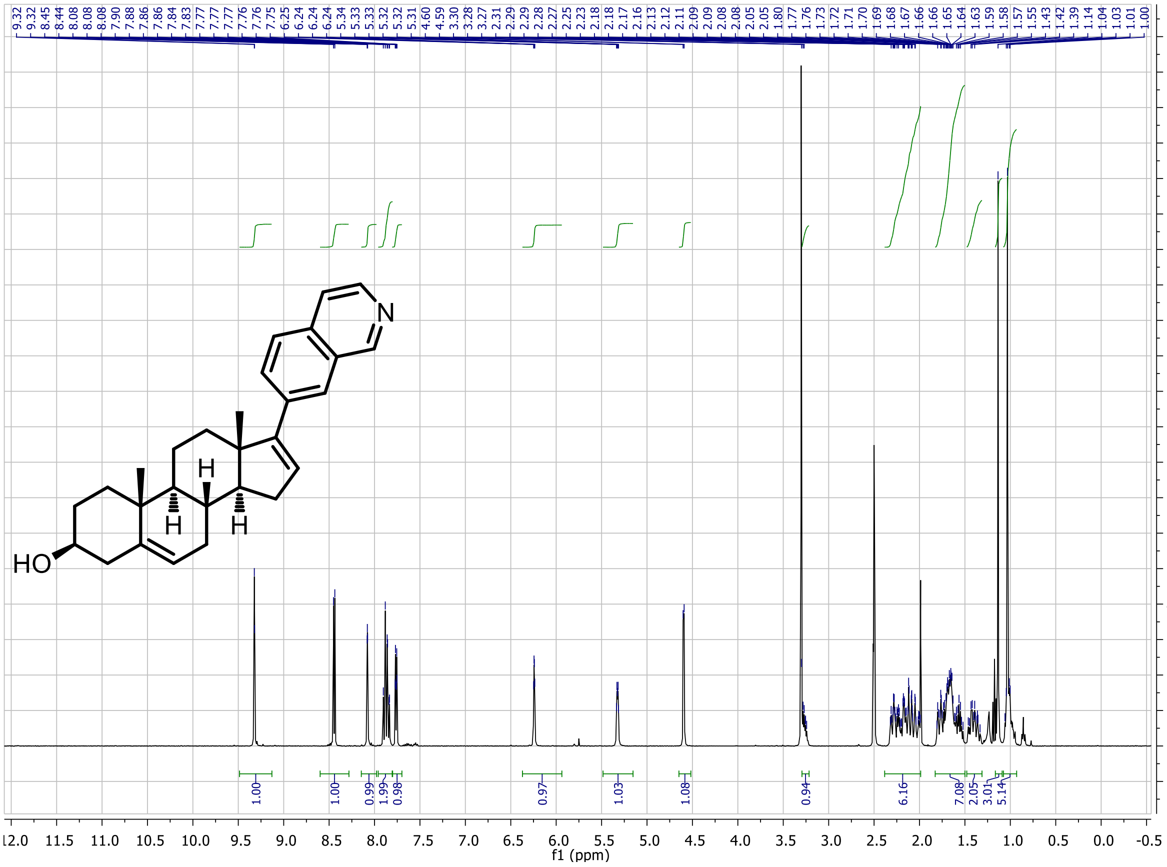

9.32 9.32 8.45 8.44 8.08 8.08 8.08 7.90 7.88 7.86 7.86 7.84 7.83 7.77 7.77 7.77 7.76 7.75 6.25 6.24 6.24 6.24 5.34 5.33 5.33 5.32 5.32 5.31 4.60 4.59 3.30 3.28 3.27 2.31 2.29 2.29 2.28 2.27 2.25 2.23 2.18 2.18 2.17 2.16 2.13 2.12 2.11 2.09 2.09 2.08 2.08 2.05 2.05 1.80 1.77 1.76 1.73 1.72 1.71 1.70 1.69 1.68 1.67 1.66 1.66 1.65 1.64 1.63 1.59 1.58 1.57 1.55 1.43 1.42 1.39 1.14 1.04 1.03 1.01 1.00
1.00 1.00 0.99 1.99 0.98 0.97 1.03 1.08 0.94 6.16 7.08 2.05 3.01 5.14
N
H
H
H
HO
f1 (ppm)

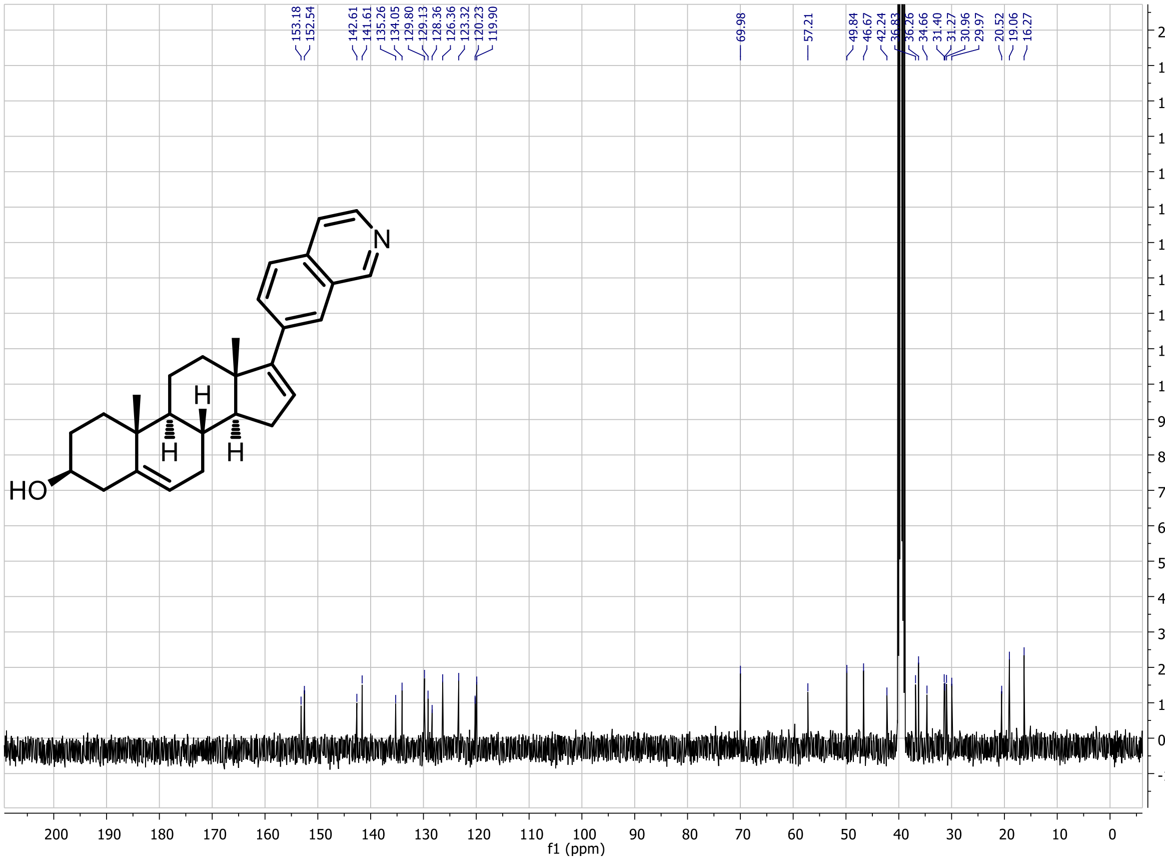

153.18 152.54 142.61 141.61 135.26 134.05 129.80 129.13 128.36 126.36 123.32 120.23 119.90 69.98 57.21 49.84 46.67 42.24 36.83 36.26 34.66 31.40 31.27 30.96 29.97 20.52 19.06 16.27
N
H
H
H
HO
f1 (ppm)

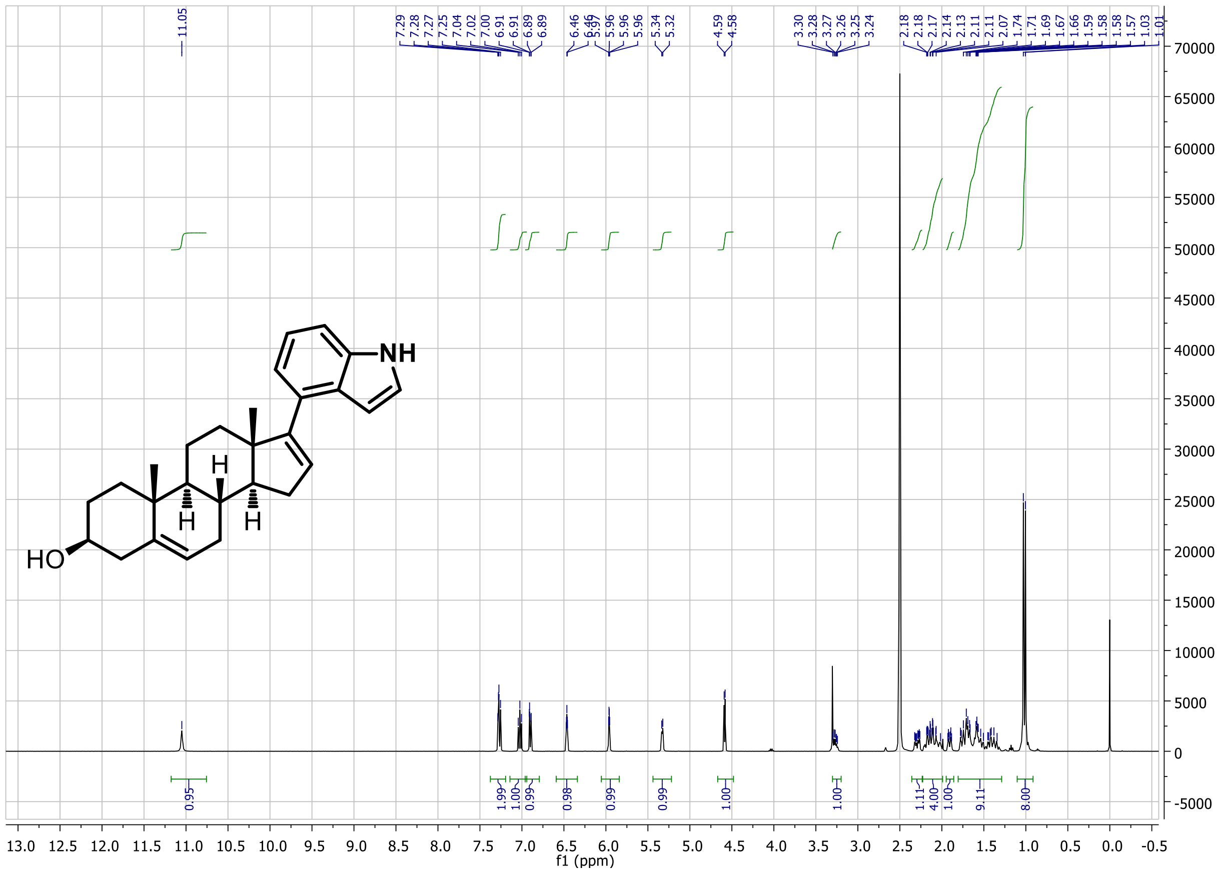
11.05
7.29
7.28
7.27
7.25
7.04
7.02
7.00
6.91
6.91
6.89
6.89
6.46
5.96
5.96
5.34
5.32
4.59
4.58
3.30
3.28
3.27
3.26
3.25
3.24
2.18
2.18
2.17
2.14
2.13
2.11
2.11
2.07
1.74
1.71
1.69
1.67
1.66
1.59
1.58
1.58
1.57
1.03
1.01
0.95
1.99
1.00
0.99
0.98
0.99
0.99
1.00
1.00
1.11
4.00
1.00
9.11
8.00
NH
H
H
H
HO
f1 (ppm)

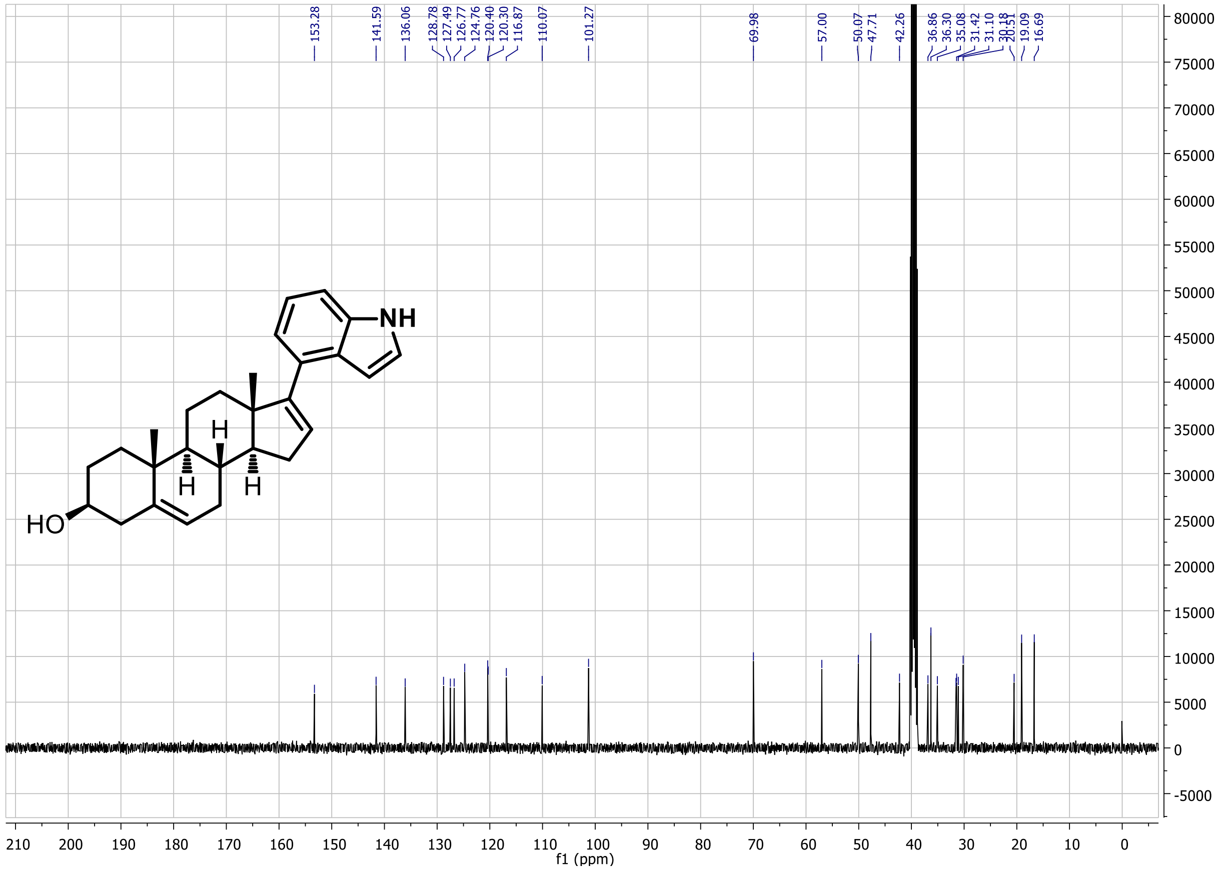
153.28
141.59
136.06
128.78
127.49
126.77
124.76
120.40
120.30
116.87
110.07
101.27
69.98
57.00
50.07
47.71
42.26
36.86
36.30
35.08
31.42
31.10
30.18
20.51
19.09
16.69
NH
H
H
H
HO
f1 (ppm)

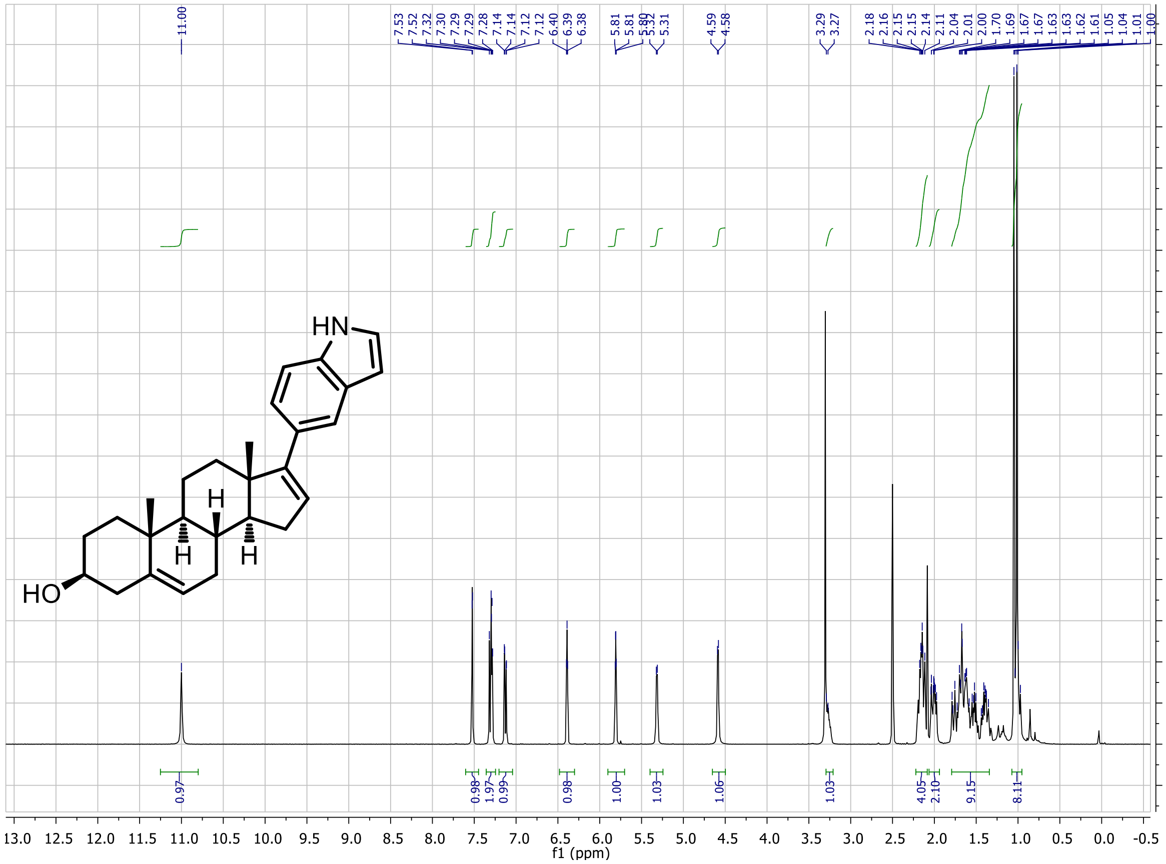

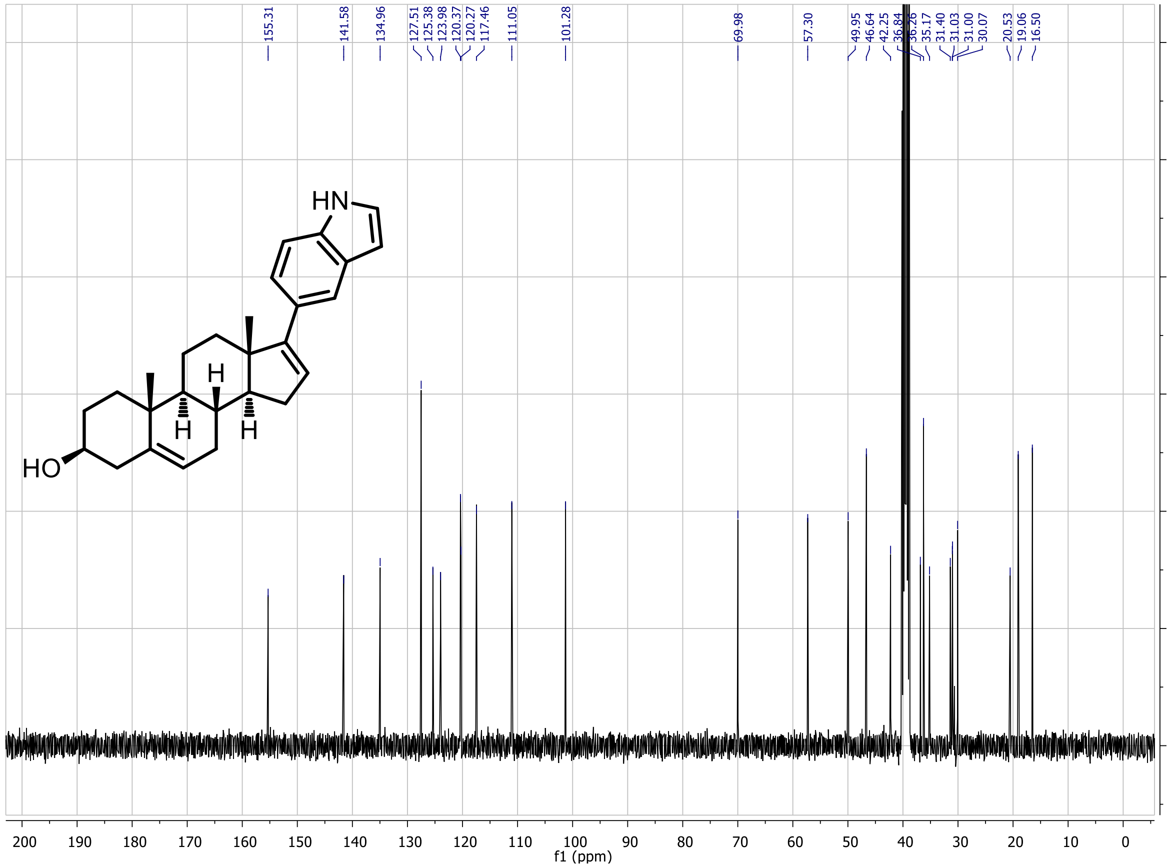

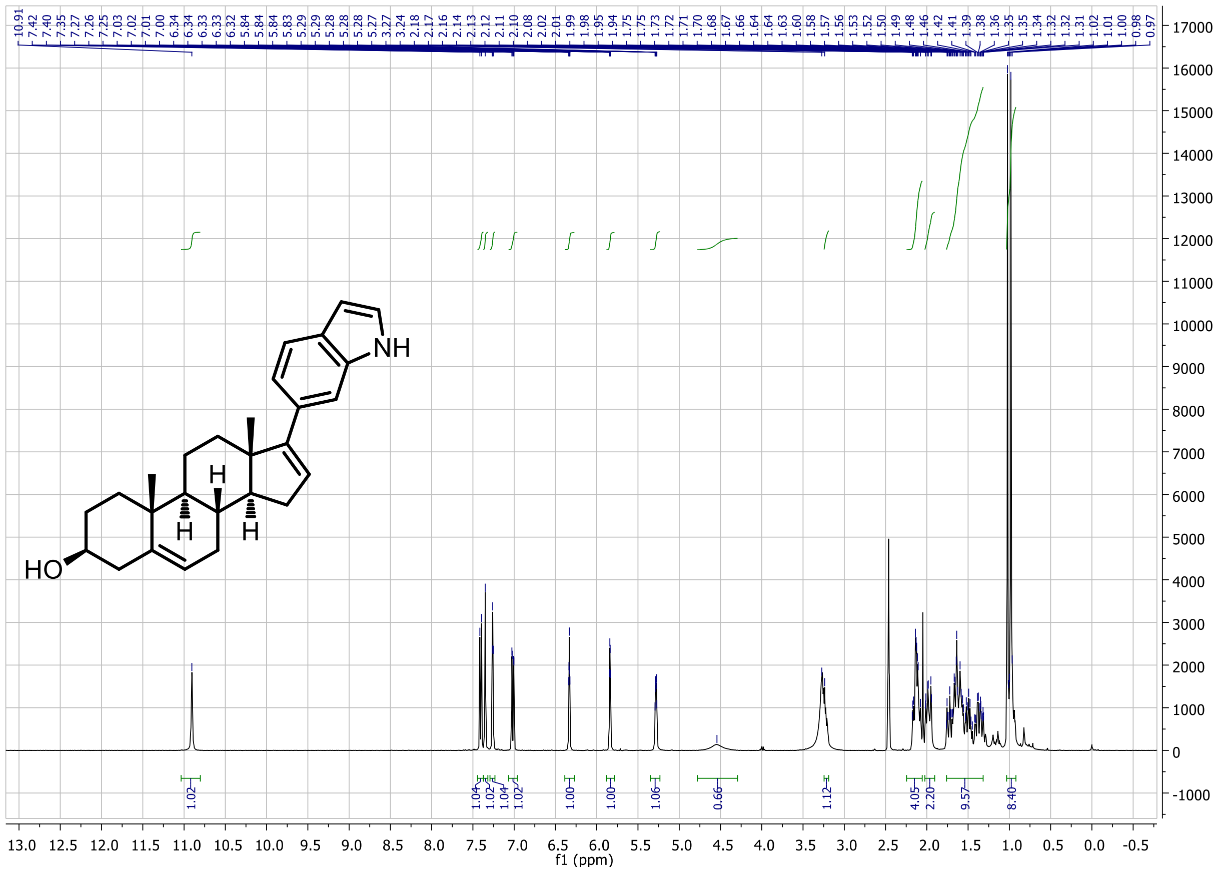

10.91 7.42 7.40 7.35 7.27 7.26 7.25 7.03 7.02 7.01 7.00 6.34 6.33 6.33 6.32 5.84 5.84 5.83 5.29 5.29 5.28 5.28 5.28 5.27 3.27 3.24 2.18 2.17 2.16 2.14 2.13 2.12 2.11 2.10 2.08 2.02 2.01 1.99 1.98 1.95 1.94 1.75 1.75 1.73 1.72 1.71 1.70 1.68 1.67 1.66 1.64 1.63 1.60 1.58 1.57 1.56 1.53 1.50 1.49 1.48 1.46 1.42 1.41 1.39 1.38 1.36 1.35 1.35 1.34 1.32 1.32 1.31 1.02 1.01 1.00 0.98 0.97
NH
H
H
H
HO
1.02
1.04
1.02
1.04
1.02
1.00
1.00
1.06
0.66
1.12
4.05
2.20
9.57
8.40
f1 (ppm)

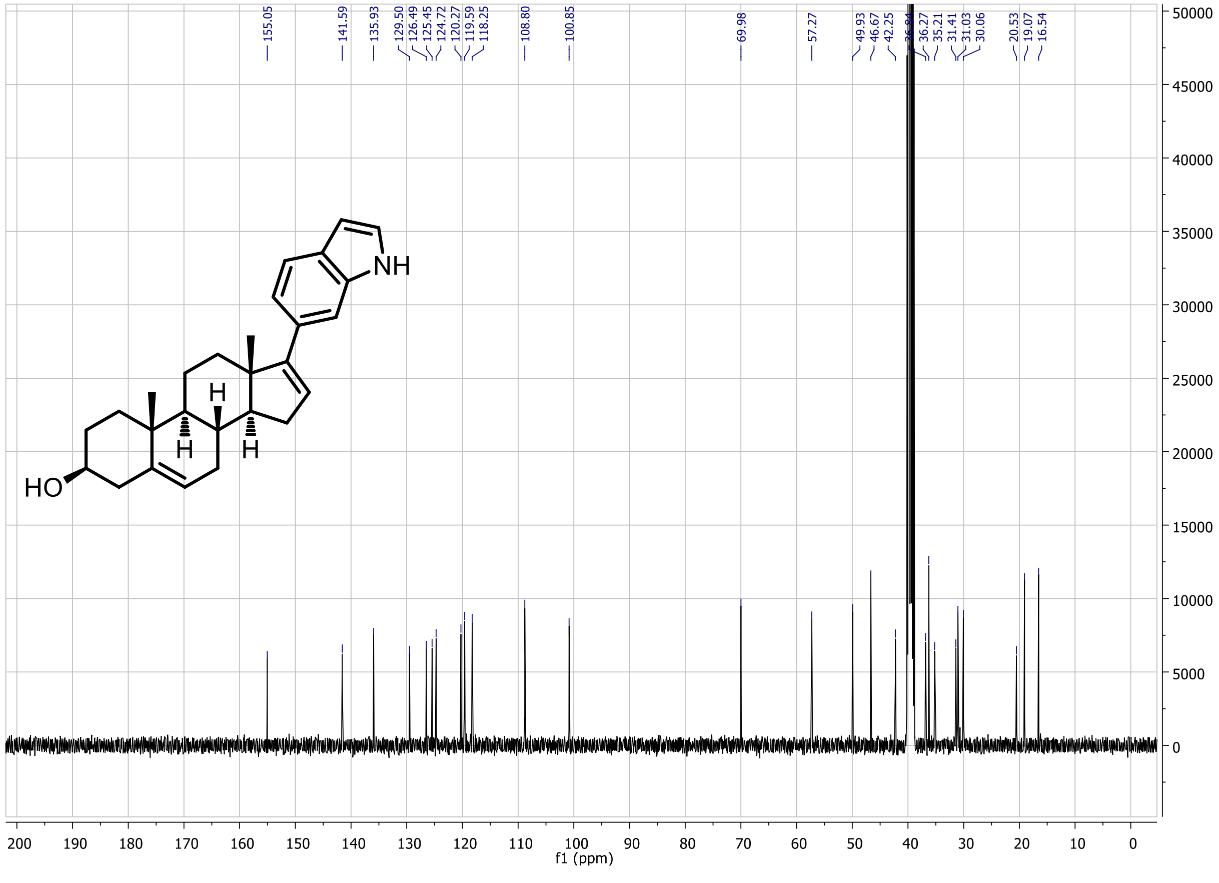

155.05
141.59
135.93
129.50
126.49
125.45
124.72
120.27
119.59
118.25
108.80
100.85
69.98
57.27
49.93
46.67
42.25
36.27
35.21
31.41
31.03
30.06
20.53
19.07
16.54
NH
H
H
H
HO
f1 (ppm)
